# SYmptom-Based STratification of DiabEtes Mellitus by Renal Function Decline (SYSTEM): A Retrospective Cohort Study and Modeling Assessment

**DOI:** 10.3389/fmed.2021.682090

**Published:** 2021-06-14

**Authors:** Kam Wa Chan, Tak Yee Chow, Kam Yan Yu, Yulong Xu, Nevin Lianwen Zhang, Vivian Taam Wong, Saimei Li, Sydney Chi Wai Tang

**Affiliations:** ^1^Department of Medicine, The University of Hong Kong, Hong Kong, China; ^2^Hong Kong Association for Integration of Chinese-Western Medicine, Hong Kong, China; ^3^School of Information Technology, Henan University of Traditional Chinese Medicine, Zhengzhou, China; ^4^Department of Computer Science and Engineering, The Hong Kong University of Science and Technology, Hong Kong, China; ^5^School of Chinese Medicine, The University of Hong Kong, Hong Kong, China; ^6^The First Affiliated Hospital, Guangzhou University of Chinese Medicine, Guangzhou, China

**Keywords:** diabetes, diabetic kidney disease, renal medicine, epidemiology, integrative medicine, symptom, diagnosis, traditional chinese medicine

## Abstract

**Background:** Previous UK Biobank studies showed that symptoms and physical measurements had excellent prediction on long-term clinical outcomes in general population. Symptoms and signs could intuitively and non-invasively predict and monitor disease progression, especially for telemedicine, but related research is limited in diabetes and renal medicine.

**Methods:** This retrospective cohort study aimed to evaluate the predictive power of a symptom-based stratification framework and individual symptoms for diabetes. Three hundred two adult diabetic patients were consecutively sampled from outpatient clinics in Hong Kong for prospective symptom assessment. Demographics and longitudinal measures of biochemical parameters were retrospectively extracted from linked medical records. The association between estimated glomerular filtration rate (GFR) (independent variable) and biochemistry, epidemiological factors, and individual symptoms was assessed by mixed regression analyses. A symptom-based stratification framework of diabetes using symptom clusters was formulated by Delphi consensus method. Akaike information criterion (AIC) and Bayesian information criterion (BIC) were compared between statistical models with different combinations of biochemical, epidemiological, and symptom variables.

**Results:** In the 4.2-year follow-up period, baseline presentation of edema (−1.8 ml/min/1.73m^2^, 95%CI: −2.5 to −1.2, *p* < *0.001*), epigastric bloating (−0.8 ml/min/1.73m^2^, 95%CI: −1.4 to −0.2, *p* = 0.014) and alternating dry and loose stool (−1.1 ml/min/1.73m^2^, 95%CI: −1.9 to −0.4, *p* = 0.004) were independently associated with faster annual GFR decline. Eleven symptom clusters were identified from literature, stratifying diabetes predominantly by gastrointestinal phenotypes. Using symptom clusters synchronized by Delphi consensus as the independent variable in statistical models reduced complexity and improved explanatory power when compared to using individual symptoms. Symptom-biologic-epidemiologic combined model had the lowest AIC (4,478 vs. 5,824 vs. 4,966 vs. 7,926) and BIC (4,597 vs. 5,870 vs. 5,065 vs. 8,026) compared to the symptom, symptom-epidemiologic and biologic-epidemiologic models, respectively. Patients co-presenting with a constellation of fatigue, malaise, dry mouth, and dry throat were independently associated with faster annual GFR decline (−1.1 ml/min/1.73m^2^, 95%CI: −1.9 to −0.2, *p* = 0.011).

**Conclusions:** Add-on symptom-based diagnosis improves the predictive power on renal function decline among diabetic patients based on key biochemical and epidemiological factors. Dynamic change of symptoms should be considered in clinical practice and research design.

## Introduction

The pathogenesis of diabetes is heterogeneous and there is a constant call for more personalized management (1, 2). Majority of existing studies stratified diabetes on the molecular level (1). Previous studies of UK Biobank suggested that phenomes, including symptoms and physical measures, had an excellent prediction of long-term clinical outcome in general population (3). Network medicine integrates phenome and genome to reclassify disease as the base of personalized medicine (4, 5). However, symptomatology is less explored in diabetes and kidney diseases.

**Figure 1:**
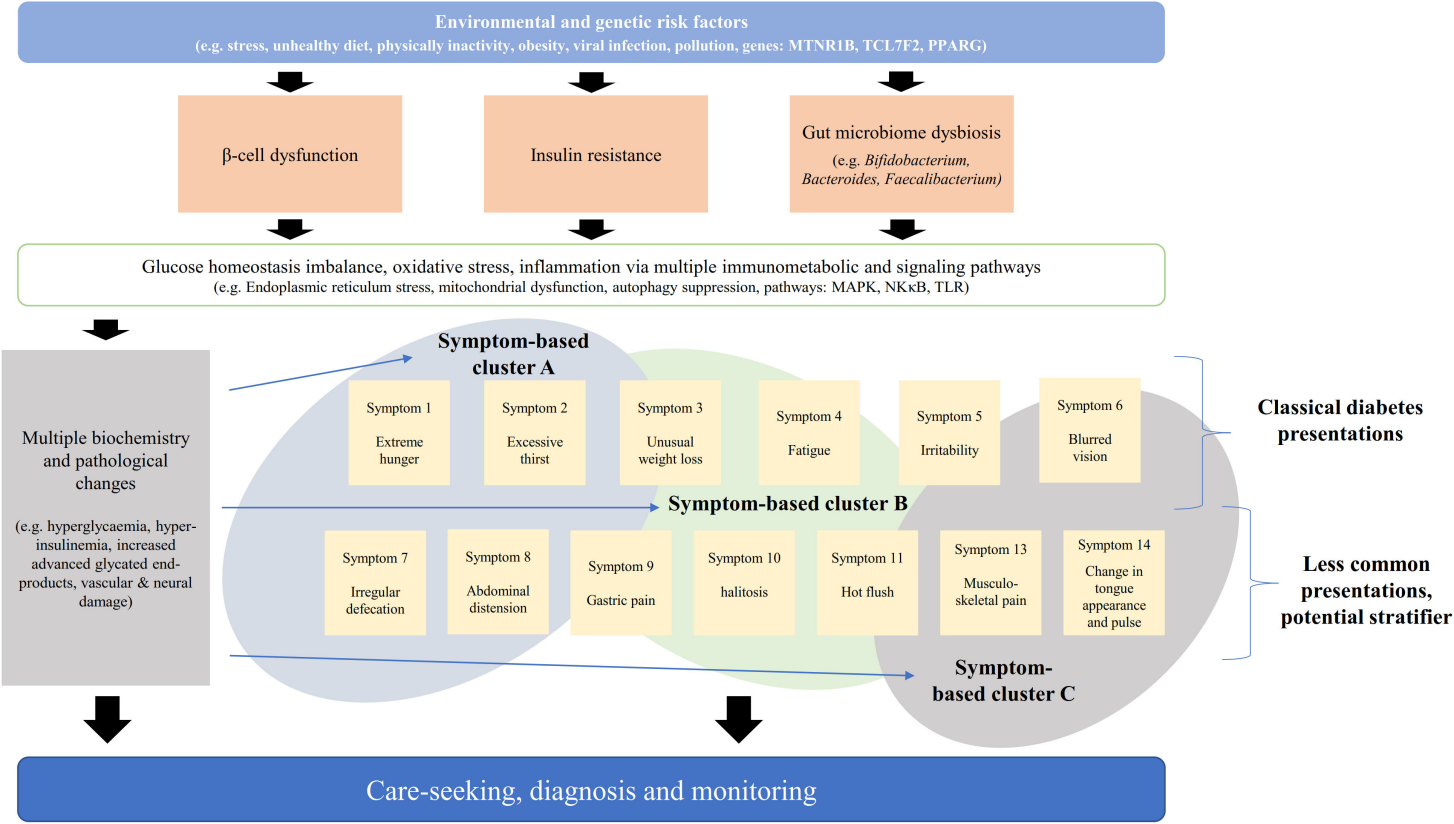
Schematic diagram of association between pathogenesis, biochemical parameters, and symptoms. The pathogenesis of diabetes is multifaceted and symptomatic presentation could reflect the whole-system response.

Symptoms and signs are often the center of patient's care (6). Symptom-based disease stratification has been utilized in diseases with considerable heterogeneity (7–9). Patient reported outcomes and symptom-based diagnosis are increasingly used in all levels of healthcare (6) including evaluation of clinical interventions (10–12), disease diagnosis, surveillance (13), and stratification (7, 14–17). Clustering of symptom is associated with specific cluster of etiology and pathogenesis (Figure 1) (7, 15, 16, 18). Different diseases of similar symptomatology share genetic associations and protein-protein interactions (9). Assessment of phenome-genomic association could also reveal more druggable targets (19, 20).

The SHIELD study showed that each of the 7 common diabetes symptoms is associated with onset of diabetes among non-diabetic population (21). Further expanding the scope of symptom analysis could identify symptoms that are specific (and therefore less commonly presented) to disease progression. However, untargeted mining of symptoms (e.g., by latent class analysis) requires an exponentially larger sample size to compensate the measurement errors arise from assessing multiple individual symptoms. Supervised and targeted analysis with qualitative inputs from clinical perspective (e.g., by Delphi consensus panel) could reduce the large number of symptoms to fewer symptom clusters for more efficient statistical modeling and clinical use. Similar dimension reduction strategy from genotype to phenotype has been used in deep learning methods to reduce sample size required to predict personalized drug response (22).

Chinese medicine (CM) has a long history in symptom-based disease management as biochemical and molecular investigations were not available at the time of theory development (23–25). Previous big data studies showed that add-on symptom-based CM treatment led to reduced risk of end-stage kidney disease and mortality among chronic kidney disease (CKD) patients with diabetes (26–28). CM subclassifies diseases based on different symptom combinations on top of conventional medicine diagnosis to formulate personalized regimens (12, 17, 29). In each symptom-based subtype (SS) of diabetes, related symptoms are weighed into core and supplementary criteria based on the importance to diagnosis (30). Although CM stratification system has the potential to refine diabetes classification, there is no synchronized framework. The benefit of add-on symptom-based stratification remains undetermined for diabetes.

Symptom could serve as an intuitive and non-invasive tool to predict and monitor clinical progression, especially for telemedicine. Previous study shows that retarding renal function decline is the priority among diabetic patients with no/early CKD (25). This study aimed to investigate the association between the presence of symptoms, SSs and renal function deterioration among diabetic patients, and to establish a synchronized symptom-based framework for stratifying diabetes based on the quality of different statistical models in predicting renal function.

## Methods

### Study Design

**Figure 2:**
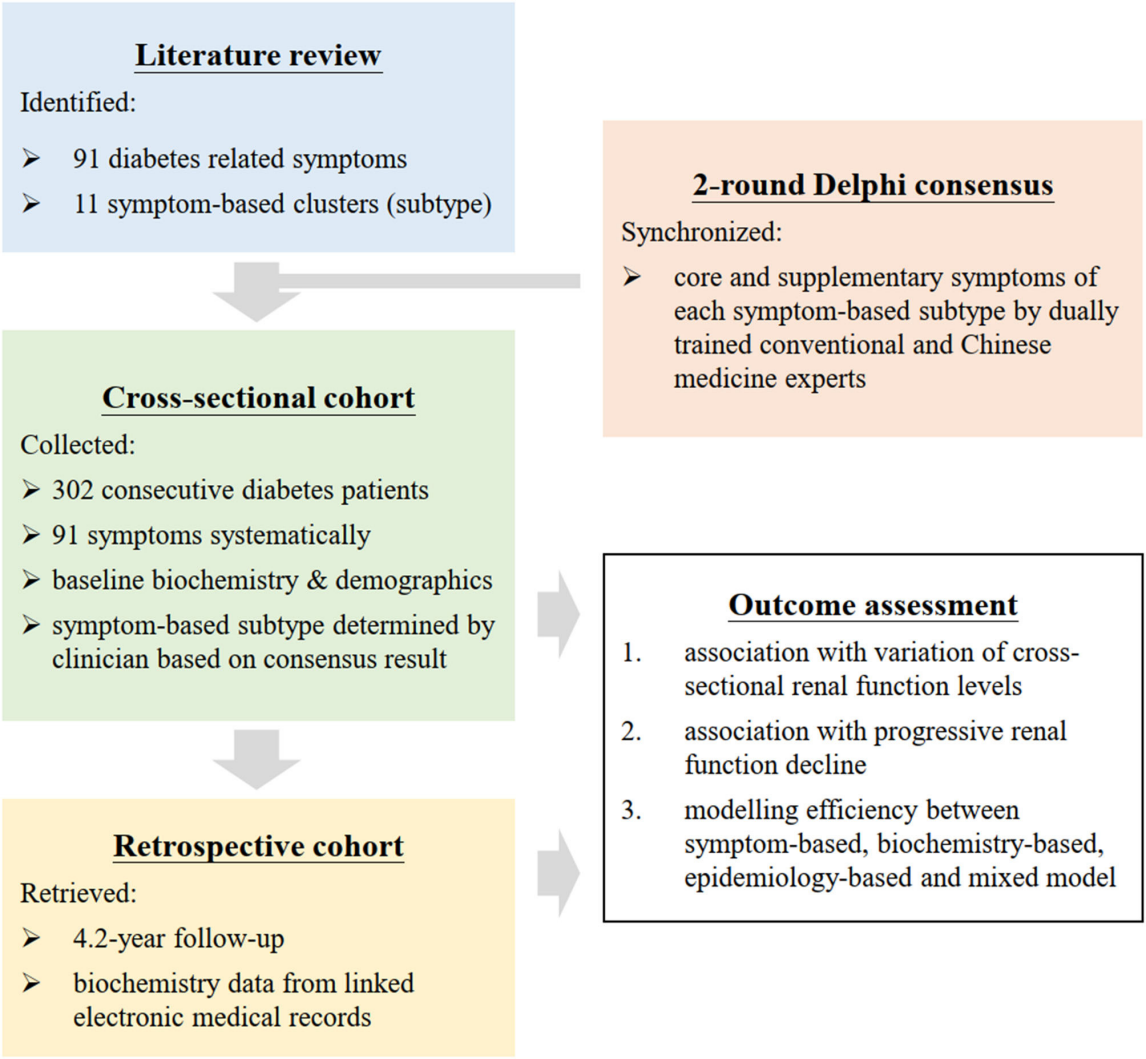
Flow diagram of research. Symptoms of diabetes were analyzed as individual symptom and symptom clusters. Presence of 91 symptoms was recorded with field tested questionnaire by 2 synchronized physicians. Symptom clusters was constructed by extraction from literature followed by a consensus panel. The explanatory power and complexity of different models with combinations of symptoms, biochemistry, and demographics were compared by Akaike information criterion and Bayesian information criterion used in machine learning.

Retrospective cohort study on the cross-sectional and longitudinal association between renal function and risk factors (Figure 2). Delphi technique (31–33) was used as the consensus methodology to construct a symptom-based classification framework for diabetes that comprised of symptom clusters. Explanatory power and complexity of statistical models with different combinations of biochemical, epidemiological, and symptom variables were compared by information criteria used in machine learning.

### Participants

Three hundred two diabetes subjects aged 18 or above on antidiabetic medication were recruited consecutively from general outpatient clinics of public hospitals in Hong Kong through a 30-min health service programme from July 2014 to December 2019. Although there is no consensus on sample size calculation for comparing model quality, modeling bias is shown to be small and independent to sampling design empirically when sample size is over 100 (34).

Ten conventional medicine and CM dually trained experts in internal medicine and classical CM theory (Supplementary Material 6) were purposively sampled to join the Delphi consensus panel to diversify the schools of theory on symptom-based diabetes diagnosis.

### Data Collection

Ninety-one symptoms, signs and behavioral factors (Supplementary Material 5) were extracted from existing literature. The presence of symptoms were prospectively collected face-to-face by physicians based on a standardized, structured, and field-tested questionnaire through the service program (35). Presence of symptoms was quantified by the days of presence within 2 weeks of consultation. Diagnosis of the 11 SSs was based on the presence of symptoms with reference to the symptom-based diagnosis framework derived from the expert consensus described below. To ensure data consistency, only 2 trained and synchronized physicians collected the data.

Biochemistry and demographics (listed in Data Analysis) of patients were retrieved from linked electronic medical record including a comprehensive and standardized annual multidisciplinary Risk Assessment and Management Programme (36). The Programme captures patients' demographics assessed by trained nurse, and the continuous change of key biochemical parameters. All outcome assessors were blinded from the analysis plan and biochemistry data to avoid bias. Longitudinal data was retrieved from the electronic medical system until 1 December 2020 by 2 researchers (K.W.C., K.Y.Y.) independently with a standardized form.

### Delphi Consensus Process and Questionnaire

Consensus methods are commonly used in constructing agreeable frameworks of existing practice in medicine (31). Delphi technique, which involve iterative anonymous rating, has advantage in handling dominant opinion and geographical constraint (32). The consensus panel was formed based on their academic capacity, peer reputation, geographical coverage, and school of thoughts to maximize the coverage of opinion while being specific to diabetes. A panel of 10 was recruited to maximize participation and consensus efficiency (32, 37). The consensus result was used to reduce the dimension of symptom analysis from individual symptoms to symptom clusters.

The Delphi consensus was divided into 2 rounds. A questionnaire with Likert scale of 1–9 was designed as the medium for rating (Supplementary Material 7). Since CM has a rich history of using symptom-based diagnosis (23, 24), we sought to expand the scope of symptom analysis by extracting CM literature. Ninety-one baseline symptoms were extracted from all 11 documented SSs from key symptom-based diabetes guideline published by academy, academic society and government regulatory bodies (Supplementary Material 2) (38–41), and standardized by a national standard of medical terminology (42). The baseline questionnaire contained 300 symptoms and could be completed in 30 min in field tests.

In the first round, experts rated anonymously the importance of the symptoms in diagnosing each SS and were invited to supplement necessary symptoms. Consensus was pre-defined as two-third majority rating 1–3 (agreed as insignificant) or 7–9 (agreed as significant). Symptoms reached consensus were then removed from the questionnaire. The result in the first round was disseminated back to and confirmed by the consensus panel. Dissonant symptoms were circulated again in the second round along with the additional symptoms collected in the first round.

Symptoms reached consensus in either rounds were defined as core criteria for the diagnosis of the corresponding SS. Sensitivity analyses with different consensus definition (average score of 6.7) and handling of missing values (last-observation-carry-forward and extreme values) were conducted. Symptoms reached consensus in sensitivity analyses were defined as supplementary criteria. Results were confirmed by panel members.

### Data Analysis

Estimated GFR was calculated by the CKD-EPI equation (43). Education (no formal/primary/secondary/tertiary), type of diabetes (type 1/type 2/gestational), obesity (optimal weight/underweight/over-weight/moderately to severely obese), and smoking (non-/ex-/current smoker) and alcohol (non-/ex-/social/current drinker) history were further stratified. Univariable and backward stepwise multivariable regression models on the correlation between GFR and binary presence of individual symptoms were evaluated. Missing values were replaced by multiple imputation with 20 sets of data using age as auxiliary variable for the cross-sectional analysis.

Annual rate of GFR change over the follow-up period (in weeks) with repeated measures was analyzed by mixed models with unstructured covariance and adjusted for (1) baseline levels of hemoglobin A1c (HbA1c), systolic blood pressure, low-density lipoprotein, log-transformed urine albumin-to-creatinine ratio (UACR), and GFR, (2) baseline presence of symptoms or SSs, (3) gender, type of diabetes, smoking history, alcohol consumption, and obesity, (4) interaction between baseline GFR and visit, (5) and interaction between presence of symptoms or SSs and visit. The included biochemical parameters and epidemiological factors are known predictors of renal function decline. Baseline presence of symptoms was used as the explanatory variable over continuous change of symptoms as the analyses were focused on prediction instead of continuous association. The slope of change was analyzed with a linear growth model with sensitivity analysis of using repeated measures of biochemical parameters and blood pressures as covariates instead of baseline values, as previous studies showed that the majority of GFR decline is linear (44).

Five multivariable regression models were built using GFR as the dependent variable to assess the power of explaining the variance in GFR, namely (1) Individual Symptom-based Model, (2) Traditional CM Model (with SSs), (3) Traditional CM-Epidemiology Model (with SSs and epidemiological variables), (4) Conventional Medicine Model (with biochemical and epidemiological variables), and (5) Integrative Chinese and Conventional Medicine Model (with SSs, biochemical and epidemiological variables) (Table 1). Both cross-sectional regression models and longitudinal mixed models were evaluated.

**Table 1:** Performance of regression models on longitudinal measurements of glomerular filtration rate.

**Model**	**Independent variable**	**Akaike information criterion**	**Bayesian information criterion**
Individual symptom-based	Binary presence of 7 symptoms^†^	10687.58	10782.25
Traditional Chinese Medicine	Binary presence of 3 symptom-based subtypes^**∧**^	5823.801	5870.134
Biochemistry and physical measures	Levels of baseline glomerular filtration rate, hemoglobin A1c, systolic blood pressure, low-density lipoprotein, log-transformed urine albumin-to-creatinine ratio	9197.525	9238.786
Epidemiology	Age, gender, type of diabetes, obesity status, smoking history, alcohol consumption^‡^	9107.248	9183.933
**Combined model**			
Traditional Chinese Medicine—Epidemiology	Binary presence of 3 symptom-based subtypes + epidemiological variables	4965.917	5064.612
Conventional medicine	Biochemistry and physical measures + epidemiological variables	7925.645	8025.706
Integrative Chinese and conventional medicine	Binary presence of 3 symptom-based subtypes + biochemistry and physical measures + epidemiological variables	4478.052	4597.48

*Backward stepwise mixed linear growth model was used to estimate the slope of glomerular filtration rate (GFR) change. Integrative Chinese and Conventional Medicine model has the lowest Akaike information criterion (AIC) and Bayesian information criterion (BIC), indicating highest quality in predicting the slope of GFR change*.

† *7 symptoms: edema, epigastric bloating, cold extremities, thin tongue, deep pulse, unsmooth pulse; ∧3 symptom-based subtypes: SS4, dampness-heat in stomach and intestine; SS8, qi and yin deficiency; SS9, liver and kidney deficiency; SS10, yin and yang deficiency;*

‡ *Epidemiological variables: age, gender (male/female), type of diabetes (type 1/type 2/gestational diabetes), duration of diabetes, obesity (optimal weight/under-weight/over-weight/moderately to severely obese), smoking history (non-/ex-/current smoker), and alcohol consumption (non-/ex-/social/current drinker)*.

Quality of the statistical models was assessed by Akaike information criterion (AIC) and Bayesian information criterion (BIC). AIC and BIC assessed the quality of statistical models in predicting GFR with different discount on model complexity, and are commonly used as model selection criteria in machine learning (45). Lower AIC and BIC refers to model that explain the variance better with less complexity, and is regarded as a better model. GFR was selected as the dependent variable for the models as diabetic kidney disease (DKD) is the most prevalent and costly complication of diabetes with limited known progression risk factors (44). Prevention of renal function decline has been suggested as the top priority among diabetic patients in our previous focus group interview series (25). The efficiency of diagnostic models in predicting rate of renal function has clinical significance (46). STATA 15.1 was used for analysis.

## Results

### Demographics

Majority of the 302 patients had type 2 diabetes, were non-smoker, centrally obese, non-insulin user and complicated with dyslipidemia, stage 2 CKD, and microalbuminuria (3.0 ± 1.1 mg/mmol) (Table 2). The mean age, GFR and HbA1c were 59.7 ± 0.7 years, 77.3 ± 1.5 ml/min/1.73m^2^ and 7.7 ± 0.1%, respectively. Most presented symptoms and signs were susceptible to infections (80.8%), nocturnal polyuria (67.4%), forgetfulness (65.6%), past frequent intake of high fat diet (59.6%), malar flush (58.3%), malaise (56.3%) knee buckling (54.6%), lumbago (54.0%), dry skin (52.3%), and fatigue (49.7%) (Supplementary Material 6). Eight patients' record were irretrievable due to missing personal identifier. Median follow-up period was 220 weeks (4.2 years) [IQR: 212–234]. Median change of GFR was −2.3 ml/min/1.73m^2^ [IQR: −8.7 to 1.4] over the period. 286 (94.7%) and 280 (92.7%) patients had annual GFR measurements until the 3rd and 4th year of follow-up.

**Table 2:** Patient demographics of validation cohort.

**Demographics**
Age—yrs	59.7 ± 0.7
Male ratio—*n*/total (%)	175/302 (58.0)
Education level—*n*/total (%)
No formal education	4/171 (2.3)
Primary education	34/171 (19.9)
Secondary education	93/171 (54.4)
Tertiary education	40/171 (23.4)
Hemoglobin A1C—%	7.7 ± 0.1
**Type of diabetes—*****n*****/total (%)**
Type 1	14/294 (4.8)
Type 2	279/294 (94.9)
Gestational	1/294 (0.3)
History of diabetes—yrs	11.6 ± 0.5
With family history—*n*/total (%)	212/283 (74.9)
**Smoking history—*****n*****/total (%)**
Non-smoker	224/302 (74.2)
Ex-smoker	53/302 (17.6)
Current smoker	25/302 (8.3)
**Alcohol consumption—*****n*****/total (%)**
Non-drinker	160/269 (59.5)
Ex-drinker	25/269 (9.3)
Social drinker	72/269 (26.8)
Current drinker	12/269 (4.5)
**Obesity—*****n*****/total (%)**
Optimal weight	69/298 (23.2)
Under-weight	5/298 (1.7)
Over-weight	130/298 (43.6)
Moderately or severely obese	94/298 (31.5)
Central obesity—*n*/total (%)	230/297 (77.4)
Dyslipidemia—*n*/total (%)	271/299 (90.6)
Insulin use—*n*/total (%)	91/302 (30.1)
**Biochemical parameters and physical measurements**
Systolic Blood pressure—mmHg	137.8 ± 1.1
^‡^Estimated glomerular filtration rate—ml/min/1.73m^2^	77.3 ± 1.5
^†^Urine albumin-to-creatinine ratio—mg/mmol	3.0 ± 1.1
Low-density lipoprotein—mmol/L	2.3 ± 0.0
^*^Symptom-based subtype (SS)—***n*****/total (%)**
SS1: (*phlegm-dampness-heat stasis*)	27/290 (9.3)
SS2: (*dampness-heat encumbering spleen*)	54/290 (18.6)
SS3: (*stagnated heat in liver and stomach*)	62/290 (21.4)
SS4: (*dampness-heat in stomach and intestine*)	27/290 (9.3)
SS5: (*spleen deficiency with stomach heat*)	45/289 (15.6)
SS6: (*heat in upper body and cold in lower body*)	8/289 (2.8)
SS7: (*yin deficiency with excessive heat*)	101/287 (35.2)
SS8: (*qi and yin deficiency*)	92/272 (33.8)
SS9: (*liver and kidney deficiency*)	182/268 (67.9)
SS10: (*yin and yang deficiency*)	23/158 (14.4)
SS11: (*blood stasis blocking collaterals*)	106/146 (72.6)

*Presented in mean ± SE,*

† *geometric mean,*

‡ *estimated by CKD-EPI equation*.

* *Presence of SS is not mutually exclusion from each SS. Detail defining symptoms are listed in Table 5*.

### Cross-Sectional Analysis of Individual Symptoms

The association between GFR and individual symptoms are summarized in Table 3. The frequency of nocturnal polyuria, defined as the reported average frequency of urination during sleep, demonstrated a strong inverse dose-dependent response with GFR (Supplementary Material 3) in the univariable analysis. In the multivariable symptom-based regression model, frequent urination (−9.1 ml/min/1.73m^2^, 95%CI: −15.7 to −2.5, *p* = 0.007), nocturnal polyuria (−12.1 ml/min/1.73m^2^, 95%CI: −17.7 to −6.5, *p* < *0.001*), darkened complexion (−9.7 ml/min/1.73m^2^, 95%CI: −17.6 to −1.8, *p* = 0.017), dark lips (−10.7 ml/min/1.73m^2^, 95%CI: −16.5 to −4.8, *p* < *0.001*) and dull purple color on lower limb (−13.5 ml/min/1.73m^2^, 95%CI: −21.8 to −5.1, *p* = 0.002) are associated with reduced renal function after adjusting the presence of other symptoms. Further adjustment with biochemical parameters and demographics showed that edema (−7.1 ml/min/1.73m^2^, 95%CI: −12.1 to −2.2, *p* = 0.005) and crenated tongue (−4.2 ml/min/1.73m^2^, 95%CI: −8.2 to −0.3, *p* = 0.037) are independently associated with reduced GFR.

**Table 3:** Cross-sectional association between glomerular filtration rate and symptoms at baseline.

	**Adjusted model^a^**			**Unadjusted multivariable model**		
**Estimated GFR (ml/min/1.73m^**2**^)**	**Regression coefficient**	***P*-value**	**95% confidence interval**	**Regression coefficient**	***P*-value**	**95% confidence interval**
**Key biochemistry and physical measurement**						
Baseline hemoglobin A1c (%)	−1.575	0.040	−3.081 to −0.069	
Log–urine albumin–to-creatinine ratio (mg/mmol)	−2.683	<0.001	−3.907 to −1.458	
**Key epidemiological factors**						
Age (yr)	−0.981	<0.001	−1.176 to −0.787	
Gender—female	34.078	<0.001	29.764 to 38.392	
**Presence of symptom or symptom–based subtype**						
Edema	−7.127	0.005	−12.069 to −2.184	
Crenated tongue	−4.234	0.037	−8.211 to −0.257	
Heat intolerance	5.281	0.011	1.220 to 9.341	9.650	0.001	4.039 to 15.261
Feverish				15.395	<0.001	7.537 to 23.253
Frequent urination				−9.099	0.007	−15.685 to −2.514
Nocturnal polyuria				−12.086	<0.001	−17.712 to −6.459
Darkened complexion				−9.706	0.017	−17.642 to −1.771
Dark lips				−10.691	<0.001	−16.534 to −4.846
Dull-purple color on lower limb				−13.447	0.002	−21.763 to −5.131

a *Adjusted for age, gender, type of diabetes, smoking history, alcohol consumption, obesity status, baseline levels of hemoglobin A1c, systolic blood pressure, low-density lipoprotein, log-transformed urine albumin-to-creatinine ratio. Statistically significant factors (p < 0.05) are shown*.

### Consensus of Diagnostic Framework Based on Symptom Clusters

The 11 SSs extracted from literature described diabetes patients that co-present with: (1) SS1: lower gastrointestinal symptoms and obesity; (2) SS2: lower gastrointestinal symptoms and malaise; (3) SS3: upper gastrointestinal symptoms that related to emotion; (4) SS4: both upper and lower gastrointestinal symptoms; (5) SS5: upper gastrointestinal symptoms and fatigue/malaise; (6) SS6: upper gastrointestinal symptoms and cold extremities; (7) SS7: general dry and hot sensation; (8) SS8: dry sensation and fatigue/malaise; (9) SS9: waist and knee musculoskeletal and otorhinolaryngological symptoms; (10) SS10: general weakness, waist and knee musculoskeletal symptoms, nocturia/frothy urine, and hyposexuality; and (11) SS11: signs of poor circulation (Supplementary Material 1).

Invitation was sent to 11 experts. One refused due to work engagement and 10 experts joined the consensus panel (Supplementary Material 6). After 2 rounds of consensus, 90 symptoms reached consensus in total for the 11 SSs (Table 4). The inter-rater Cohen's Kappa between consensus definition of two-third majority and average score of 6.7 was 0.90 overall (0.89 and 0.90 for the first and second round of consensus, respectively). Sensitivity analyses showed that 39 symptoms reached consensus with consensus definition of 6.7 average score or after imputation of missing values. They were listed as supplementary criteria that add likelihood for the classification of the corresponding SS (Table 4).

**Table 4:** Delphi consensus result.

**Symptom-based subgroup^**∧**^^**±**^**	**Consensus in 1^**st**^ round (*n* = 10)**	**Consensus in 2^**nd**^ round (*n* = 8)^**Δ**^**	**Consensus in sensitivity analysis**
**SS1:** Co-present with lower gastro-intestinal symptoms and obese	Past frequent intake of high fat diet (80%), dry stool or dyssynergic defecation with loose stool (70%), yellowish fur on tongue (70%), greasy fur on tongue (80%)	Slippery pulse (75%)	Sticky stool^*^^a^, Obese^a^^,^^b^^,^^c^, malaise^b^^,^^c^, Sticky feeling in mouth^a^^,^^c^
**SS2:** Co-present with lower gastro-intestinal symptoms and malaise	Malaise (70%), abdominal distention (70%), sustained satiety (70%), red tongue (70%), yellowish fur on tongue (70%), greasy fur on tongue (80%)	Dyssynergic defecation (75%)	Slippery pulse^a^^,^^b^^,^^c^
**SS3:** Co-present with upper gastro-intestinal symptoms and emotion related	Burning sensation in stomach (70%), epigastric pain (70%), bitter taste (70%), red tongue (80%), yellowish fur on tongue (80%), string pulse (70%)	Epigastric bloating (75%), vexation (75%), dry mouth (75%)	Epigastric fullness^a^^,^^b^^,^^c^, dry stool^a^^,^^b^^,^^c^, flank distention^a^^,^^c^,, flank distress^a^^,^^c^
**SS4:** Co-present with both upper and lower gastro-intestinal symptoms	Abdominal bloating (70%), halitosis (70%), red tongue (80%), yellowish fur on tongue (80%), greasy fur on tongue (80%), slippery pulse (70%)	Dry stool or dyssynergic defecation with loose stool (75%)	Epigastric bloating^a^, abdominal fullness^a^, abdominal distention^a^
**SS5:** Co-present with upper gastro-intestinal symptoms and fatigue/malaise	Fatigue (70%), malaise (70%), epigastric bloating (70%), epigastric fullness (70%), epigastric distention (70%), alternating dry, and loose stool (80%)	^*^Yellow greasy fur and pale red tongue (87.5)	Burning sensation in stomach^a^, gastric discomfort^a^, Greasy fur^c^
**SS6:** Co-present with upper gastro-intestinal symptoms and cold extremities	Epigastric bloating (70%), epigastric fullness (70%), loose stool (70%)	Cold extremities (75%), yellowish fur on tongue (75%)	Burning sensation in stomach^a^, red tongue^a^^,^^b^^,^^c^, bitter taste^a^^,^^c^
**SS7:** Co-present with general dry and hot sensation	Vexation (70%), malar flush (70%), dry mouth (70%), frequent thirsty (90%), dry throat (70%), dry stool and constipation (70%), red tongue (70%), scanty fur on tongue (70%), dry fur (70%), rapid pulse (80%), thready pulse (90%)	None	Irascibility^a^, yellowish urine^a^, burning sensation on chest, palms and soles^a^, yellowish fur^a^^,^^b^^,^^c^, dry tongue^a^^,^^c^ nocturnal sweating^^*^a, c^
**SS8:** Co-present with dry sensation and fatigue/malaise	Fatigue (80%), malaise (100%), dry mouth (70%), dry throat (70%), thready pulse (80%)	None	Weak pulse^a^^,^^b^^,^^c^
**SS9:** Co-present with waist and knee musculoskeletal and otorhino-laryngological symptoms	Dry mouth (70%), lumbago (80%), lumbar weakness (80%), knee pain (80%), knee buckling (70%), scanty fur on tongue (70%), thready pulse (70%)	None	Blurred vision^a^, red tongue^a^^,^^b^^,^^c^, tinnitus^b^^,^^c^
**SS10:** Co-present with general weakness, waist, and knee musculoskeletal symptoms, nocturia/frothy urine, and hyposexuality	Pale complexion (70%), shortness of breath (70%), fatigue (80%), malaise (90%), dry mouth (70%), cold sensation around lumbar region (70%), knee pain (70%), cold sensation on knees (80%), high susceptibility to infectious disease (70%), nocturnal polyuria (70%), frothy urine (70%), frequent urination with small volume (70%), loose stool (70%), dark lips (70%), dull tongue (70%), swollen tongue (80%), crenated tongue (80%), weak pulse (80%), weak *chi*-pulse (80%)	Lumbago (100%), cold extremities (75%), deep pulse (75%)	Limb oedema^a^, sallow complexion^a^, hyposexuality^a^^,^^c^, white fur on tongue^a^, thready pulse^a^, impotence^a^^,^^c^
**SS11:** Co-present with signs of poor circulation	Limb numbness (70%), ecchymosis on tongue (70%), sublingual varicosities (70%), unsmooth pulse (70%)	Dysmenorrhea^*^ (66.7%)	Dull-purple color on lower limb^a^^,^^b^^,^^c^, xerosis^b^^,^^c^, dark lips^a^^,^^b^^,^^c^, dull tongue^a^, menstrual clot^c^

∧ *SS1, Phlegm-dampness-heat stasis; SS2, dampness-heat encumbering spleen; SS3, Stagnated heat in liver and stomach; SS4, dampness-heat in stomach and intestine; SS5, spleen deficiency with stomach heat; SS6, heat in upper body and cold in lower body; SS7, Yin deficiency with excessive heat; SS8, qi and yin deficiency; SS9, liver and kidney deficiency; SS10, yin and yang deficiency; SS11, blood stasis blocking collaterals;*

* *Supplemented by experts in first round of consensus;*

a *through consensus definition of 6.7;*

b *through last-observation-carry-forward for missing values imputation;*

c *through extreme values for missing values imputation*.

Δ *2 panel members dropped out in the 2^nd^ round due to retirement and geographical relocation, and heavy work engagement*.

### Longitudinal Analysis of Individual Symptoms and Symptom-Based Subtypes

The AIC and BIC of the 5 models are presented in Table 1. The Integrative Chinese and Conventional Medicine Model had the lowest AIC (4,478 vs. 5,824 vs. 4,966 vs. 7,926) and BIC (4,597 vs. 5,870 vs. 5,065 vs. 8,026) compared to the Traditional CM, Traditional CM-Epidemiology, and Conventional Medicine models, respectively. Using SS as the explanatory variable in statistical models resulted in better model quality with lower AIC and BIC when compared to the models using individual symptoms.

**Figure 3:**
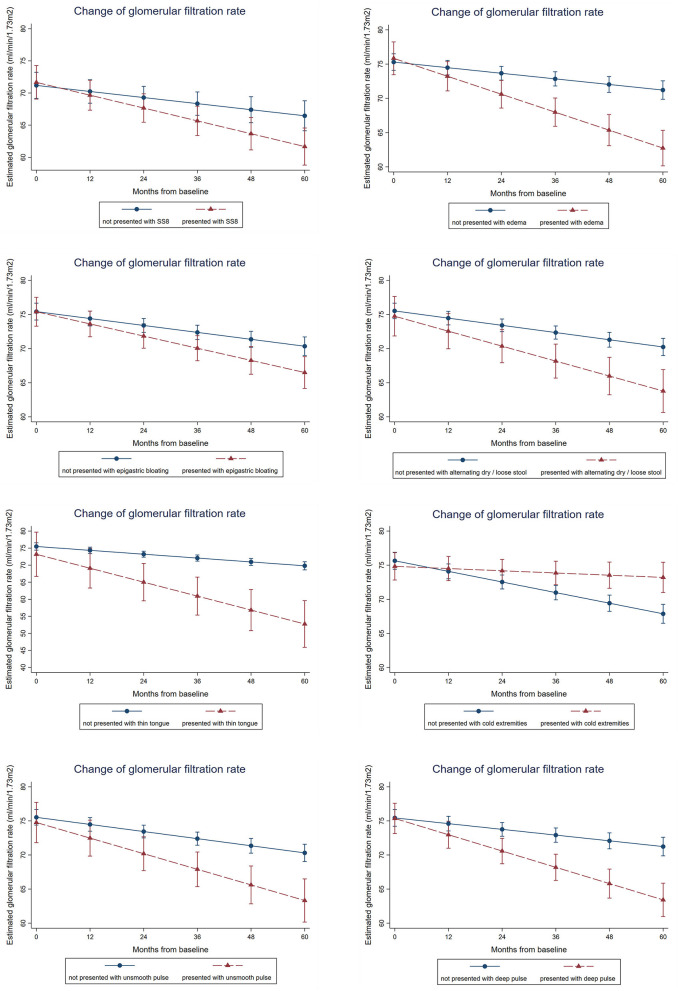
Association between presence of symptoms, symptom clusters and renal function decline over time. Mixed linear growth model on longitudinal repeated measures was used to estimate the slope of GFR change. After adjusting age, gender, type of diabetes, smoking history, alcohol consumption, obesity status, the baseline hemoglobin A1c, systolic blood pressure, low-density lipoprotein, and log-transformed urine albumin-to-creatinine ratio, patients presented with symptom-based subtype 8 (SS8) is associated with 1.1 ml/min/1.73m^2^ more annual GFR drop, whereas patients presented with edema, epigastric bloating, alternating dry and loose stool, thin tongue, deep pulse, or unsmooth pulse is associated with 1.8, 0.8, 1.1, 3.0, 1.5, or 1.2 ml/min/1.73m^2^ more annual GFR drop. Result is robust in sensitivity analyses using repeated measures of biochemical parameters and blood pressures as covariates for adjustment.

From the multivariable regression model of Integrative Chinese and Conventional Medicine, the presence of SS8 (fatigue, malaise, dry mouth, dry throat, weak pulse) is associated −1.1 ml/min/1.73m^2^, 95%CI: −1.9 to −0.2, *p* = 0.011 more annual GFR drop after adjusting key biochemical parameters and demographics. In the symptom-based multivariable model using individual symptoms instead of SSs, edema (−1.8 ml/min/1.73m^2^, 95%CI: −2.5 to −1.2, *p* < *0.001*), epigastric bloating (−0.8 ml/min/1.73m^2^, 95%CI: −1.4 to −0.2, *p* = 0.014), alternating dry and loose stool (−1.1 ml/min/1.73m^2^, 95%CI: −1.9 to −0.4, *p* = 0.004), thin tongue (−3.0 ml/min/1.73m^2^, 95%CI: −4.5 to −1.4, *p* < *0.001*), deep pulse (−1.5 ml/min/1.73m^2^, 95%CI: −2.2 to −0.9, *p* < *0.001*), and unsmooth pulse (−1.2 ml/min/1.73m^2^, 95%CI: −2.0 to −0.5, *p* = 0.002) were significantly and independently associated with faster GFR decline (Figure 3, Table 5). Result is robust in sensitivity analyses using repeated measures of biochemical parameters and blood pressures as covariates for adjustment.

**Table 5:** Longitudinal association between slope of glomerular filtration rate change, epidemiological factors, symptoms, and biochemical parameters by follow-up repeated measures.

	**Model with individual symptoms**			**Model with symptom-based subtype**		
**Estimated GFR (ml/min/1.73m^**2**^)**	**Regression coefficient^**a**^**	***p*-value^**a**^**	**95% confidence interval**	**Regression coefficient^**a**^**	***p*-value^**a**^**	**95% confidence interval**
**Key biochemistry and physical measurement**						
Baseline GFR (ml/min/1.73m^2^)	0.916	<0.001	0.853 to 0.978	0.853	<0.001	0.762 to 0.945
Log-urine albumin-to-creatinine ratio (mg/mmol)	−1.347	<0.001	−1.947 to −0.748	−3.081	<0.001	−3.999 to −2.162
**Association between presence of symptom or symptom-based subtype and monthly GFR measures**						
Edema	−0.150	<0.001	−0.204 to −0.096			
Epigastric bloating	−0.064	0.013	−0.115 to −0.013			
Cold extremities	0.103	<0.001	0.053 to 0.152			
Alternating dry and loose stool	−0.095	0.004	−0.158 to −0.031			
Thin tongue	−0.246	<0.001	−0.375 to −0.118			
Deep pulse	−0.129	<0.001	−0.181 to −0.076			
Unsmooth pulse	−0.104	0.001	−0.168 to −0.040			
Symptom-based subtype 4				0.116	0.042	0.004 to 0.228
Symptom-based subtype 8				−0.088	0.011	−0.155 to −0.020
Symptom–based subtype 10				0.121	0.014	0.024–0.218

*Mixed linear growth model on longitudinal repeated measures was used to estimate the slope of GFR change*.

a *Monthly difference (ml/min/1.73m^2^) and p–value comparing slope of GFR change between patients with or without the baseline presence of the symptoms and symptom-based subtypes. After adjusting age, gender, type of diabetes, smoking history, alcohol consumption, obesity status, the baseline control of blood glucose (hemoglobin A1c), blood pressure (systolic blood pressure), lipids (low–density lipoprotein), and urine albumin (log-transformed urine albumin-to-creatinine ratio), patients presented with symptom-based subtype 8 is associated with 1.1 ml/min/1.73m^2^ more annual GFR drop, whereas patients presented with edema, epigastric bloating, alternating dry and loose stool, thin tongue, deep pulse or unsmooth pulse is associated with 1.8, 0.8, 1.1, 3.0, 1.5, or 1.2 ml/min/1.73m^2^ more annual GFR drop*.

## Discussion

This is the first attempt to compare modeling efficiency of renal function in diabetes with different combinations of biochemical parameters, demographics, and symptoms. We synchronized the symptom-based stratification framework through expert consensus and identified individual symptoms which better predicts longitudinal renal function decline. The demographics of our cohort was comparable to that from general outpatient clinics in Hong Kong and other studies globally with considerable generalizability (47). Patients had a moderate level of HbA1c and the symptoms presented were likely residual to glycemic control which require attention.

### Current Evidence of Symptom Clustering and Limitation

Symptoms developed from clustering of pathophysiology (9, 13, 20) and have been used in disease monitoring, or as a treatment response predictor (7, 14). Distinctive clustering of frequent urination, excessive thirst, extreme hunger and unusual weight loss has characterized diabetes since 500 B.C.. Increased fatigue, irritability and blurry vision were supplemented since then. These symptoms have also been demonstrated to correlate with diabetes onset among non-diabetic population in the SHIELD study (21).

Independent associations have been established between symptoms and CKD. For instance, dermatological changes (e.g., pruritus, xerosis) were shown to correlate with uricemia (48). DKD was also defined as a composite of clinical presentations (edema, heart failure), epidemiology (long standing diabetes, hypertension), histopathology (nodular glomerulosclerosis, thickened basement membranes), and biochemistry (increased urine albumin) by Paul Kimmelstiel in 1936 (49). Symptoms that associate with the renal progression could further stratify patients for personalized management.

Currently, there is no standardized symptom-based stratification framework for diabetes (50). We identified discrepancies in the listing of commonly presented SSs and the related symptoms across different guidelines. Our newly synchronized framework is more concise. For instance, the defining symptoms of SS1 were simplified from 31 possible symptoms to 9 key symptoms (Supplementary Material 1). This facilitates the comparison of utility between individual symptoms and symptom clusters.

### Symptoms Are Associated With Poorer Renal Function

Infection susceptibility, forgetfulness, lumbago, knee buckling, and malar flush were commonly presented in our cohort. Classical presentation of frequent thirst and excessive hunger was less frequent, likely because the patients were already on hypoglycaemic agents that alleviated these symptoms.

From the regression analysis without adjustment of biochemical and epidemiological factors, a strong inverse dose-dependent relationship between GFR and nocturnal polyuria was observed, indicating a quantifiable assessment on nocturnal polyuria in clinical practice. Frequent urination, nocturnal polyuria, darkened complexion, dark lips, and dull purple color on lower limb are associated with lower GFR, which is commonly observed in practice.

After adjusting biochemical parameters and demographics including age, gender, HbA1c, and UACR, a set of different symptoms (edema, crenated tongue) were associated with poorer renal function independently. This is due to the collinearity between symptoms and biochemical parameters (e.g., frequent urination and UACR).

Using symptoms from the unadjusted symptom-based model can estimate renal function in resources limited setting or in mass population screening without requiring laboratory investigations. Symptoms from the adjusted model, meanwhile, can increase the explanatory power on top of known risk factors and indicated pathophysiology independent to the control of glucose, blood pressure, lipid, and albuminuria.

### Symptom Clusters Predict 4-Year Renal Function Decline Independently

Model quality assessment showed that using symptom clusters provides better quality in modeling renal function when compared to using individual symptoms. This is due to the natural collinearity of symptoms in which the local dependence between symptoms can be better analyzed by grouping symptoms with shared pathophysiology and predictive power on renal function (51).

From the 4-year follow-up cohort, symptom clusters provide additional explanatory power on top of biochemical and epidemiological factors in stratifying the decline of renal function. Early diabetes presented with SS8, which included fatigue, malaise, dry mouth and dry throat, were associated with 1.1 ml/min/1.73m^2^ faster renal function decline annually.

### Cohering Gastrointestinal Involvement and Potential Mechanism

The presence of epigastric bloating, and alternating dry and loose stool are associated with faster GFR decline after adjusting blood glucose, blood pressure, lipid and urine albumin. From the 11 SSs that documented in CM literature, diabetes was stratified into three main groups (Figure 1): (1) SS1–SS6: with gastrointestinal presentation, (2) SS7, SS8: with classical diabetes presentations, and (3) SS9–SS11: with neurological and vascular presentations. The symptom-based stratification of diabetes depends heavily (6 of 11 SSs) on gastrointestinal presentations.

The symptom analysis and expert consensus converged to suggest an important role of gastrointestinal involvement in the renal progression of diabetes. Previous studies showed that gut microbiome is involved in the inflammatory and immune response in metabolic diseases, CKD and related phenotypical changes (52, 53). We previously demonstrated *in vivo* that toll-like receptor signaling pathway regulate DKD progression (54–56). Activation of the pathway via Paneth cells in small intestine due to microbiota dysbiosis can be a possible mechanism warrants further study (57). Endotoxin (e.g., lipopolysaccharide) translocation to systemic circulation due to disruption of gut barrier can also accelerate CKD progression (53).

### Challenges in Symptom Standardization and Recent Advancement

Symptom-based research offers a new perspective in assessing correlations in pathogenesis among diseases, accelerating the discovery of secondary application of existing drugs (29). In this study, edema is a strongly independent cross-sectional and longitudinal predictor of reduced renal function. Further multi-level omics analysis stratifying the presence of edema with matching GFR and other confounders could reveal novel mechanisms in renal function decline among early diabetic patients.

Key challenge in symptom-based research is the standardization of subjective symptoms (9). Although the unharmonized definition of symptoms is likely to introduce random measurement error instead of systematic bias, and is unlikely to increase type I error in assessing model quality, a large sample size is needed to demonstrate statistical significance. In this study, thin tongue, deep pulse, and unsmooth pulse were strongly associated with faster renal function decline. Although these symptoms have long been suggested as signs of reduced kidney function and poor circulation in CM theory, standardized, and digitalized measurement is lacking until the past decade (58–60). Recent advancement in phenotype-oriented network in multi-omics data provided a platform to link up phenotype, genotype, omics, and drug targets for the integration of clinical presentation, genetics, and epigenetics (5, 9, 50, 61, 62). These advancements enhance the clinical translation and generalizability of symptom-based diagnosis research to the other parts of the world. Nevertheless, the description of pulse would require substantial work on standardization before being widely utilized.

### Limitations

Our cohort was conveniently sampled from public general outpatient clinics. Although the sampling is not random, selection bias was minimized by consecutive sampling. Majority of the diabetes patients in Hong Kong have follow-ups in public general outpatient clinics. The demographics of our cohort matched the territory-wide demographics (47). Besides, there is no standardized questionnaire for the assessment of symptoms internationally. We developed a data entry form for this purpose with field tests, and only recorded the presence of symptoms to minimize the error due to physicians' judgment. Error in symptom assessment is likely random error instead of systematic bias as the assessors were blinded from data analysis plan and biochemistry data. The measurement error is unlikely to increase type I error in assessing model quality. Furthermore, only a small group of experts with outstanding and diversified academic and clinical track record and was purposively sampled to form the consensus panel for better participation and coordination, which common for Delphi technique (32). Further large-scale untargeted mining by latent class analysis can identify the natural clustering of symptoms. In the analysis, the prescription was not analyzed as the control of blood glucose, pressure, lipid, and urine albumin were reflected by the change of HbA1c, systolic blood pressure, low-density lipoprotein, and UACR. Lastly, this study was designed to compare the quality of model performance and was not powered to evaluate the longitudinal predictiveness of each SS. The SSs that did not reach statistical significance are subject to type II error. Further large-scale correlation study is needed to precisely assess the clinical and statistical significance of each SS.

## Conclusion

Our findings showed that symptoms, in particular gastrointestinal symptoms, are strongly and independently associated with renal function decline among diabetic patients. Add-on symptom-based stratification improved the classification of diabetes in predicting glomerular filtration rate decline. Our study proved the concept and clinical value of using symptoms and symptom clusters to predict disease progression either alone or in combination with key biochemical and epidemiological factors. Further prediction algorithms of mixed molecular-symptom diagnosis are warranted. Dynamic change of symptoms should be considered in clinical practice and research design.

## Existing Evidence

Previous UK Biobank studies showed that phenomes, including symptoms and physical measurements, had excellent prediction on long-term clinical outcomes in general population. Symptom-based research in diabetes and renal medicine is limited when compared to other disciplines.

## Added Value

- In this 4-year cohort, edema, and gastrointestinal symptoms were independently associated with faster renal function decline among diabetic patients after adjusting epidemiological factors and control of blood glucose, blood pressure, lipid, and albuminuria.
- Using symptom clusters instead of individual symptoms as the independent variable in statistical models reduced computational dimension and improved model quality (increased explanatory power and less complexity).
- Clustering analysis revealed that patients presenting with a constellation of fatigue, malaise, dry mouth and dry throat were independently associated with faster renal function decline.

## Implication

Add-on symptom-based diagnosis improves the predictive power on renal function decline among diabetic patients in addition to key biochemistry and epidemiological factors. Further prediction algorithms of mixed molecular-symptom diagnosis are warranted. Dynamic change of symptoms should be considered in clinical practice and research design.

## Data Availability Statement

The raw data supporting the conclusions of this article will be made available by the authors, without undue reservation.

## Ethics Statement

The studies involving human participants were reviewed and approved by Institutional Review Board of the University of Hong Kong/Hospital Authority Hong Kong West Cluster. Written informed consent for participation was not required for this study in accordance with the national legislation and the institutional requirements.

## Author Contributions

KC, SL, and ST conceived the study. KC, SL, and VW coordinated the Delphi interview. KC collected the data. KC, TC, and VW coordinated the cross-sectional study. TC collected the symptom data. KY retrieved longitudinal follow-up biochemistry and epidemiology data. KC, YX, and NZ performed the analysis. KC and ST drafted the manuscript. All authors involved in the interpretation of data and manuscript revision.

## Conflict of Interest

The authors declare that the research was conducted in the absence of any commercial or financial relationships that could be construed as a potential conflict of interest.

## Abbreviations

AIC: Akaike information criterion
BIC: Bayesian information criterion
CI: confidence interval
CKD: chronic kidney disease
CM: Chinese medicine
DKD: diabetic kidney disease
GFR: glomerular filtration rate
HbA1c: hemoglobin A1c
SS: symptom-based subtype
UACR: urine albumin-to-creatinine ratio.
