# Supplementary material for: SYmptom-Based STratification of DiabEtes Mellitus by Renal Function Decline (SYSTEM): A Retrospective Cohort Study and Modeling Assessment
Source: Front Med (Lausanne). 2021 Jun 14;8:682090. doi: 10.3389/fmed.2021.682090 (PMC8236588; doi:10.3389/fmed.2021.682090)

**SYmptom-based STratification of diabEtes Mellitus by renal function decline (SYSTEM):** **a retrospective cohort study and modelling assessment**

**Supplementary File**

|  |  | **Page** |
| --- | --- | --- |
| S1 | Baseline documented diabetes-related symptoms from literature | 2 |
| S2 | Clinical and research guidelines on Chinese medicine for diabetes | 5 |
| S3 | Correlation between frequency of nocturnal polyuria and renal function | 6 |
| S4 | Regression analysis of symptom subtypes and renal function decline | 7 |
| S5 | List of symptoms and frequency | 9 |
| S6 | Expertise, academic and clinical background of the experts of Delphi consensus panel | 12 |
| S7 | Delphi consensus questionnaire (in Chinese) | 14 |

**S1 Baseline documented diabetes-related symptoms from literature**

| **Symptom-based subgroup^^±^** | **Documented diabetes-related symptoms from literature** |
| --- | --- |
| **SS1:**  Co-present with lower gastro-intestinal symptoms and obese | Obese, past frequent intake of high fat diet, malaise, heavy head, syncope, abdominal distension, feverish, vexation, production of yellow sputum, dry mouth, sticky feeling in mouth, bitter taste, frequent thirsty and like cold drinks, dry stool or dyssynergic defecation with loose stool, yellowish urine, red tongue, yellowish fur on tongue, greasy fur on tongue, slippery pulse, rapid pulse, string pulse, symptoms exaggerated after taking oily food or in humid environment |
| **SS2:**  Co-present with lower gastro-intestinal symptoms and malaise | Obese, malaise, heavy head, abdominal distention, sustained satiety, nausea, vomiting, anorexia, reduced appetite, chest distress, vexation, dyssynergic defecation, yellowish urine, red tongue, yellowish fur on tongue, greasy fur on tongue, slippery pulse, rapid pulse |
| **SS3:**  Co-present with upper gastro-intestinal symptoms and emotion related | Obese, epigastric bloating, abdominal bloating, epigastric fullness, abdominal fullness, burning sensation in stomach, epigastric pain, chest distention, flank distention, chest distress, flank distress, feverish, vexation, malar flush, dry mouth, bitter taste, dry stool, yellowish urine, red tongue, yellowish fur on tongue, string pulse, rapid pulse |
| **SS4:**  Co-present with both upper and lower gastro-intestinal symptoms | Epigastric bloating, abdominal bloating, epigastric fullness, abdominal fullness, epigastric distention, abdominal distention, nausea, vomiting, frequent hungry, sore throat, gingival bleeding, dry mouth, bitter taste, halitosis, frequent thirsty and like cold drinks, dry stool or dyssynergic defecation with loose stool, purulent stool, rectal tenesmus, red tongue, ecchymosis on tongue, sublingual varicosities, yellowish fur on tongue, greasy fur on tongue, slippery pulse, rapid pulse |
| **SS5:**  Co-present with upper gastro-intestinal symptoms and fatigue/malaise | Weight loss, fatigue, malaise, syncope, epigastric bloating, epigastric fullness, epigastric distention, nausea, anorexia, gastric discomfort, burning sensation in stomach, gastric pain, hiccup, reduced appetite, defecating undigested food, feverish, vexation, insomnia, palpitation, sputum production, frequent thirsty, alternating dry or loose stool, borborygmi, pale tongue, swollen tongue, sublingual varicosities, white fur on tongue, greasy tongue, string pulse, slippery pulse, weak pulse |
| **SS6:**  Co-present with upper gastro-intestinal symptoms and cold extremities | Cold extremities, burning sensation in stomach, epigastric bloating, epigastric fullness, nausea, vomiting, borborygmi, loose stool, vexation, bitter taste, red tongue, sublingual varicosities, yellowish fur on tongue, curdy and greasy fur on tongue root, string pulse, slippery pulse |
| **SS7:**  Co-present with general dry and hot sensation | Syncope, dazzle, frequent hungry, feverish sensation on chest, palms and soles, vexation, heat intolerance, afternoon feverish, malar flush, irascibility, palpitation, vivid dreams, dry mouth, frequent thirsty, bitter taste, dry throat, frequent thirsty like cold drinks, spontaneous sweating, dry stool and constipation, yellowish urine, dry tongue, red tongue, scanty fur on tongue, yellowish fur on tongue, dry fur on tongue, weak pulse, string pulse, rapid pulse, thready pulse, slippery pulse |
| **SS8:**  Co-present with dry sensation and fatigue/malaise | Weight loss, sallow complexion, oedema, syncope, dazzle, palpitation, shortness of breath, fatigue, malaise, frequent hungry, reduced appetite, abdominal distention, chest distress, loose stool, feverish sensation on chest, palms and soles, vexation, malar flush, vivid dreams, dry mouth, dry throat, dry cough, spontaneous sweating, nocturnal sweating, lumbago, lumbar weakness, knee pain, knee buckling, spermatorrhea, premature ejaculation, red tongue, less fur on tongue, exfoliative fur on tongue, weak pulse, thready pulse, rapid pulse |
| **SS9:**  Co-present with waist and knee musculoskeletal and otorhino-laryngological symptoms | Dry skin, itchy skin/pruritus, flank pain, syncope, dazzle, tinnitus, feverish sensation on chest, palms and soles, feverish, malar flush, dry mouth, dry throat, vivid dreams, spermatorrhea, lumbago, lumbar weakness, knee pain, knee buckling, blurred vision, reduced night vision, floaters in vision, frequent urination, frothy urine, red tongue, scanty fur on tongue, thready pulse, rapid pulse |
| **SS10:**  Co-present with general weakness, waist and knee musculoskeletal symptoms, nocturia/frothy urine and hyposexuality | Puffy face, limb oedema, darkened complexion, pale complexion, sallow complexion, scorched dry auricle, syncope, dazzle, tinnitus, chest distress, abdominal distention, palpitation, shortness of breath, fatigue, malaise, feverish sensation on chest, palms and soles, vexation, dry mouth, dry throat, anorexia, reduced appetite, lumbago, cold sensation around lumbar region, knee pain, cold sensation on knees, cold extremities, impotence, spermatorrhea, premature ejaculation, hyposexuality, spontaneous sweating, high susceptibility to infectious disease, nocturnal polyuria, frothy urine, frequent urination with small volume, alternating dry or loose stool, loose stool, morning diarrhea, dark lips, dull tongue, swollen tongue, crenated tongue, white fur on tongue, dry tongue, deep pulse, thready pulse, weak pulse, weak *chi*-pulse |
| **SS11**  Co-present with signs of poor circulation | Limb numbness, limb pain, dull purple color on lower limbs, xerosis, stroke, sluggish speech, chest distress, forgetfulness, palpitation, vexation, blurred vision, dark lips, dull tongue, ecchymosis on tongue, sublingual varicosities, thin fur on tongue, white fur on tongue, string pulse, unsmooth pulse, deep pulse |

**S2 Clinical and research guidelines on Chinese medicine for diabetes**

| **Guideline** | **Issuer** | **Listed symptom-based clustering subtypes^** | | | | | | | | | | |
| --- | --- | --- | --- | --- | --- | --- | --- | --- | --- | --- | --- | --- |
|  |  | **SS1** | **SS2** | **SS3** | **SS4** | **SS5** | **SS6** | **SS7** | **SS8** | **SS9** | **SS10** | **SS11** |
| Guideline for Traditional Chinese Medicine Diabetes Prevention and Treatment | China Association of Chinese Medicine |  |  |  |  |  |  |  |  |  |  |  |
| Type 2 Diabetes Clinical Practice Guideline | National Administration of Traditional Chinese Medicine |  |  |  |  |  |  |  |  |  |  |  |
| Guiding Principle of Clinical Research on New Drugs of Chinese Medicine | State Food and Drug Administration^&^ |  |  |  |  |  |  |  |  |  |  |  |
| Type 2 Diabetes Mellitus Chinese Medicine Clinical Practice Guideline | China Academy of Chinese Medical Sciences |  |  |  |  |  |  |  |  |  |  |  |

^SS1=*Phlegm-dampness-heat stasis*; SS2=*dampness-heat encumbering spleen*; SS3= *Stagnated heat in liver and stomach*; SS4= *dampness-heat in stomach and intestine*; SS5=*spleen deficiency with stomach heat*; SS6=*heat in upper body and cold in lower body*; SS7=*Yin deficiency with excessive heat*; SS8=*qi and yin deficiency*; SS9=*liver and kidney deficiency*; SS10=*yin and yang deficiency*; SS11=*blood stasis blocking collaterals*; ^&^Renamed as National Medical Products Administration

**S3 Correlation between frequency of nocturnal polyuria and renal function**

| **Presence of nocturnal polyuria** | **Regression coefficient of GFR** | **p-value** | **95% confidence interval** | | |
| --- | --- | --- | --- | --- | --- |
| 1 | -10.740 | 0.002 | -17.655 | to | -3.824 |
| 2 | -13.965 | 0.001 | -22.247 | to | -5.682 |
| 3 | -17.566 | <0.001 | -26.844 | to | -8.288 |
| 4 | -36.959 | <0.001 | -51.482 | to | -22.436 |
| 5 | -34.704 | 0.045 | -68.576 | to | -0.833 |
| 6 | -55.205 | 0.023 | -102.859 | to | -7.551 |
| 7 | -8.706 | 0.538 | -36.504 | to | 19.092 |
| 8 | -71.301 | 0.003 | -118.955 | to | -23.647 |

Univariable analysis on the correlation between presence of nocturnal polyuria (recorded as the reported average frequency of urination during sleep) and estimated glomerular filtration rate (GFR) (ml/min/1.73m^2^). The inverse dose-dependent association remained robust in multivariable model adjusting other symptoms.

**S4 Regression analysis of symptom subtypes and renal function decline**

|  | | **Univariable analysis** | | **^3^ Multi-variable analysis** | |
| --- | --- | --- | --- | --- | --- |
|  |  | **Annual slope of GFR change (95% CI)** | **^1^ Difference in GFR slope**  **(95% CI, *p*)** | **Annual slope of GFR change (95% CI)** | **Difference in GFR slope**  **(95% CI, *p*)** |
| **SS1** | No | -1.313 (-1.637 to -0.990) | 0.611 (-0.338 to 1.560, p=0.207) |  | |
|  | Yes | -0.703 (-1.610 to 0.205) |  |  |  |
| **SS2** | No | -1.430 (-1.771 to -1.088) | 0.775 (0.072 to 1.477, p= 0.031) ^2^ |  | |
|  | Yes | -0.655 (-1.292 to -0.018) |  |  |  |
| **SS3** | No | -1.374 (-1.717 to -1.032) | 0.529 (-0.169 to 1.228, p=0.138) |  | |
|  | Yes | -0.845 (-1.477 to -0.214) |  |  |  |
| **SS4** | No | -1.380 (-1.698 to -1.062) | 1.436 (0.342 to 2.529, p=0.010) ^2^ | -1.487 (-1.924 to -1.050) | 1.393 (0.050 to 2.736, p=0.042) |
|  | Yes | 0.056 (-1.003 to 1.114) |  | -0.094 (-1.382 to 1.194) |  |
| **SS5** | No | -1.384 (-1.717 to -1.053) | 0.784 (-0.000 to 1.569, p=0.050) |  | |
|  | Yes | -0.601 (-1.332 to 0.131) |  |  |  |
| **SS6** | No | -1.330 (-1.643 to -1.018) | 2.459 (0.883 to 4.034, p=0.002) |  | |
|  | Yes | 1.128 (-0.425 to 2.681) |  |  |  |
| **SS7** | No | -1.579 (-1.958 to -1.200) | 0.846 (0.260 to 1.432, p=0.005) ^2^ |  | |
|  | Yes | -0.733 (-1.211 to -0.255) |  |  |  |
| **SS8** | No | -1.000 (-1.386 to -0.612) | -0.897 (-1.529 to -0.266, p=0.005) ^2^ | -0.945 (-1.466 to -0.424) | -1.051 (-1.858 to -0.245, p=0.011) |
|  | Yes | -1.897 (-2.428 to -1.365) |  | -1.997 (-2.646 to -1.348) |  |
| **SS9** | No | -1.552 (-2.095 to -1.008) | 0.394 (-0.247 to 1.035, p= 0.228) |  | |
|  | Yes | -1.158 (-1.547 to -0.769) |  |  |  |
| **SS10** | No | -1.547 (-1.998 to -1.097) | 1.564 (0.399 to 2.730, p=0.009) ^2^ | -1.540 (-1.986 to -1.094) | 1.455 (0.295 to 2.614, p=0.014) |
|  | Yes | 0.017 (-1.079 to 1.113) |  | -0.086 (-1.175 to 1.003) |  |
| **SS11** | No | -0.836 (-1.679 to 0.008) | -0.913 (-1.869 to 0.043, p=0.061) |  | |
|  | Yes | -1.749 (-2.272 to -1.226) |  |  |  |

Mixed linear growth model was used to estimate the slope of GFR change. ^1^ Difference (ml/min/1.73m^2^) and *p*-value comparing slope of GFR change between patients with or without the presence of symptom-based subtype 1 to 11. ^2^ robust in sensitivity analysis adjusting longitudinal repeated measures of biomarkers. ^3^ Backward stepwise regression model was used in the multivariable analysis. The presence of symptom-based subtype (SS) 4, 8 and 10 remained as independent variables with statistical significance in the multivariable analysis. Patients presented with symptom-based subtype 8 is associated with -1.051 ml/min/1.73m^2^ more annual GFR drop after adjusting age, gender, type of diabetes, smoking history, alcohol consumption, obesity, the baseline control of blood glucose (hemoglobin A1c), blood pressure (systolic blood pressure), lipids (low density lipoprotein), urine albumin (log-transformed urine albumin-to-creatinine ratio) and the presence of other SSs.

**S5 List of symptoms and frequency**

| **Symptom / sign** | **Frequency n/total (%)** |
| --- | --- |
| Edema | 60/302 (19.9) |
| Feverish | 39/302 (12.9) |
| Feverish sensation on chest, palms and soles | 24/302 (8.0) |
| Heat intolerance | 99/302 (32.8) |
| Vexation | 11/302 (3.6) |
| Spontaneous sweating | 53/301 (17.6) |
| Nocturnal sweating | 51/302 (16.9) |
| Shortness of breath | 106/302 (35.1) |
| Fatigue | 150/302 (49.7) |
| Malaise | 170/302 (56.3) |
| Heavy head | 43/302 (14.2) |
| Syncope | 55/302 (18.2) |
| Gingival bleeding | 64/302 (21.2) |
| Dry cough | 92/302 (30.5) |
| Reduced appetite | 59/302 (19.5) |
| Frequent hungry | 89/302 (29.5) |
| Past frequent intake of high fat diet | 180/302 (59.6) |
| Epigastric bloating | 73/302 (24.2) |
| Epigastric pain | 35/302 (11.6) |
| Anorexia | 31/302 (10.3) |
| Nausea | 9/302 (3.0) |
| Vomiting | 38/302 (12.6) |
| Abdominal distension | 60/302 (20.0) |
| Chest distress | 63/302 (20.9) |
| Angina pectoris | 35/292 (12.0) |
| Palpitation | 72/302 (23.8) |
| Limb numbness | 121/302 (40.1) |
| Lumbago | 163/302 (54.0) |
| Knee buckling | 165/302 (54.6) |
| Cold extremities | 90/302 (29.8) |
| Dry skin | 158/302 (52.3) |
| Itchy skin | 143/302 (47.4) |
| Tinnitus | 75/302 (24.8) |
| Forgetfulness | 198/302 (65.6) |
| Irritable | 111/302 (36.8) |
| Impetuous | 114/302 (37.8) |
| Symptoms exaggerated after taking oily food or in humid condition | 97/302 (32.1) |
| Dry stool | 40/301 (13.3) |
| Loose stool | 88/302 (29.1) |
| Alternating dry or loose stool | 48/302 (15.9) |
| Rectal tenesmus | 24/301 (8.0) |
| Dyssynergic defecation | 63/302 (20.9) |
| Defecating undigested food | 50/302 (16.6) |
| Yellowish urine | 84/302 (27.8) |
| Frothy urine | 13/302 (4.3) |
| Frequent urination | 61/301 (20.3) |
| Nocturnal polyuria | 203/301 (67.4) |
| Blurred vision | 141/302 (46.7) |
| Reduced night vision | 102/302 (33.8) |
| Floaters in vision | 92/302 (30.5) |
| Dry eye | 112/302 (37.1) |
| Insomnia | 103/302 (34.1) |
| Vivid dream | 111/302 (36.8) |
| Susceptible to infections | 244/302 (80.8) |
| Dry mouth | 127/302 (42.1) |
| Bitter taste | 78/302 (25.8) |
| Halitosis | 122/302 (40.4) |
| Sticky mouth | 71/302 (23.5) |
| Sore throat | 28/302 (9.3) |
| Dry throat | 83/302 (27.5) |
| Sputum production | 82/302 (27.2) |
| Yellowish sputum | 33/302 (10.9) |
| Darkened complexion | 36/302 (11.9) |
| Pale complexion | 24/301 (8.0) |
| Sallow complexion | 101/302 (33.4) |
| Red complexion | 69/301 (22.9) |
| Malar flush | 176/302 (58.3) |
| Puffy face | 48/302 (15.9) |
| Scorched auricle | 25/302 (8.3) |
| Dull purple color on lower limbs | 35/302 (11.6) |
| Xerosis | 49/302 (16.2) |
| Sluggish speech | 3/302 (1) |
| Red tongue | 179/302 (59.3) |
| Pale tongue | 47/302 (15.6) |
| Dull tongue | 50/302 (16.6) |
| Purple tongue | 4/302 (1.3) |
| Ecchymosis on tongue margins | 68/302 (22.5) |
| Swollen tongue | 93/302 (30.8) |
| Thin tongue | 7/302 (2.3) |
| Fissured tongue | 96/302 (31.8) |
| Crenated tongue | 141/302 (46.7) |
| Dry tongue | 86/302 (28.5) |
| Sublingual varicosities | 81/302 (26.8) |
| Dark lips | 83/302 (27.5) |
| Yellowish fur on tongue | 86/302 (28.5) |
| White fur on tongue | 145/302 (48.0) |
| Dark fur on tongue | 5/302 (1.7) |
| Exfoliative fur on tongue | 15/302 (5.0) |
| Scanty fur on tongue | 111/301 (36.9) |
| Thin fur on tongue | 130/302 (43.1) |
| Dry fur on tongue | 80/302 (26.5) |
| Thick fur on tongue | 34/302 (11.3) |
| Greasy fur on tongue | 108/301 (35.9) |
| Curdy and greasy fur on tongue root | 21/302 (7.0) |
| Moisten tongue | 18/302 (6.0) |
| Watery tongue | 24/302 (8.0) |
| Slippery pulse | 174/302 (57.6) |
| Unsmooth pulse | 41/302 (13.6) |
| Weak pulse | 101/302 (33.4) |

**S6 Expertise, academic and clinical background of the experts of Delphi consensus panel**

|  | **Expertise** | **Region^#^** | **Affiliation** | **Academic qualification** | **Clinical qualification** |
| --- | --- | --- | --- | --- | --- |
| XIONG Manqi | Endocrinology (diabetes) | GZ/HK | University / Hospital | Chair professor, PhD supervisor, director of research centre, president of academic societies | Over 50 years of practice, department head, consultant physician, academic lead |
| LI Saimei | Endocrinology (diabetes) | GZ | University / Hospital | Professor, PhD / Post-doctoral supervisor, director of research centre, president of academic societies | Over 30 years of practice, department head, consultant physician, academic lead |
| FAN Guanjie | Endocrinology (diabetes) | GZ | Hospital / University | Professor, PhD supervisor, vice president of academic societies | Over 30 years of practice, department head, consultant physician, academic lead |
| ZHU Zhangzhi | Endocrinology (diabetes) | GZ | University / Hospital | Professor, PhD supervisor, director of research centre, president of academic societies | Over 30 years of practice, department head, consultant physician |
| LIU Min | Endocrinology (diabetes) | GZ | University / Hospital | Professor, PhD supervisor, vice president of academic societies | Over 30 years of practice, deputy department head, consultant physician |
| ZHANG Juan | Endocrinology (diabetes) | SD/HK | University | Professor, postgraduate supervisor | Over 30 years of practice, deputy department head, consultant physician |
| BIAN Zhaoxiang | Internal medicine, research methods | BJ/NJ/  HK/GZ | University | Chair professor, PhD supervisor, director of research centre, president of academic societies | Over 30 years of practice, department head, consultant physician, academic lead |
| XU Daji | Internal medicine  (nephrology) | GZ/HK | University | Professor, postgraduate supervisor | Over 30 years of practice, consultant physician, Academic Successor of National Chinese Medicine Physicians |
| XUE Yiming | Internal medicine, Theories of Various Schools of Thoughts | NJ/HK | University | Professor, PhD supervisor, director of research centre, president of academic societies | Over 40 years of practice, department head, consultant physician, academic lead |
| SU Jing | Internal medicine, Classics of Chinese medicine | BJ/HK | University / Hospital | Professor, PhD supervisor | Over 30 years of practice, consultant physician, Academic Successor of National Chinese Medicine Physicians |

**^#^** BJ=Beijing; GZ=Guangzhou; HK=Hong Kong; NJ=Nanjing; SD=Shandong

**S7 Delphi consensus questionnaire (in Chinese)**

**Round 1**


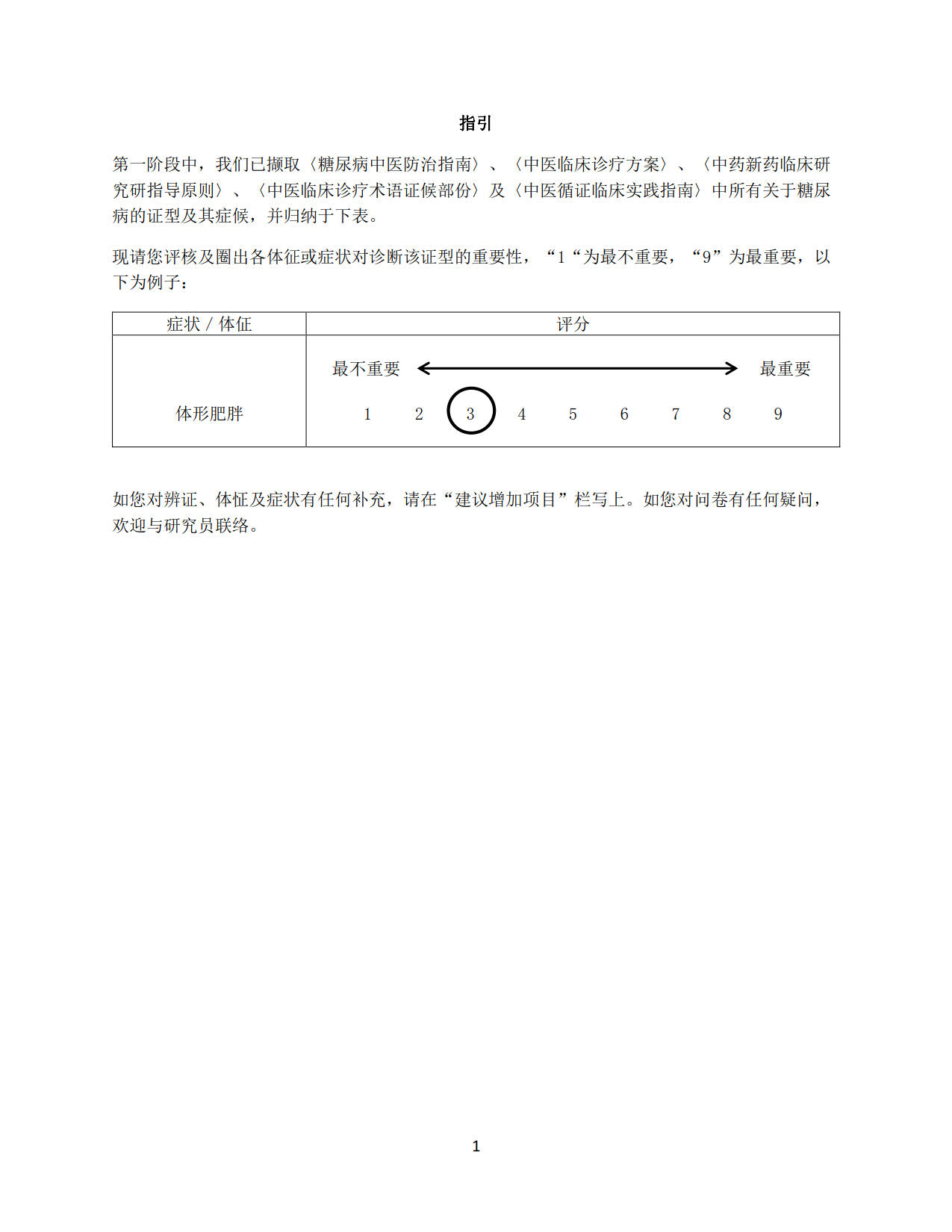

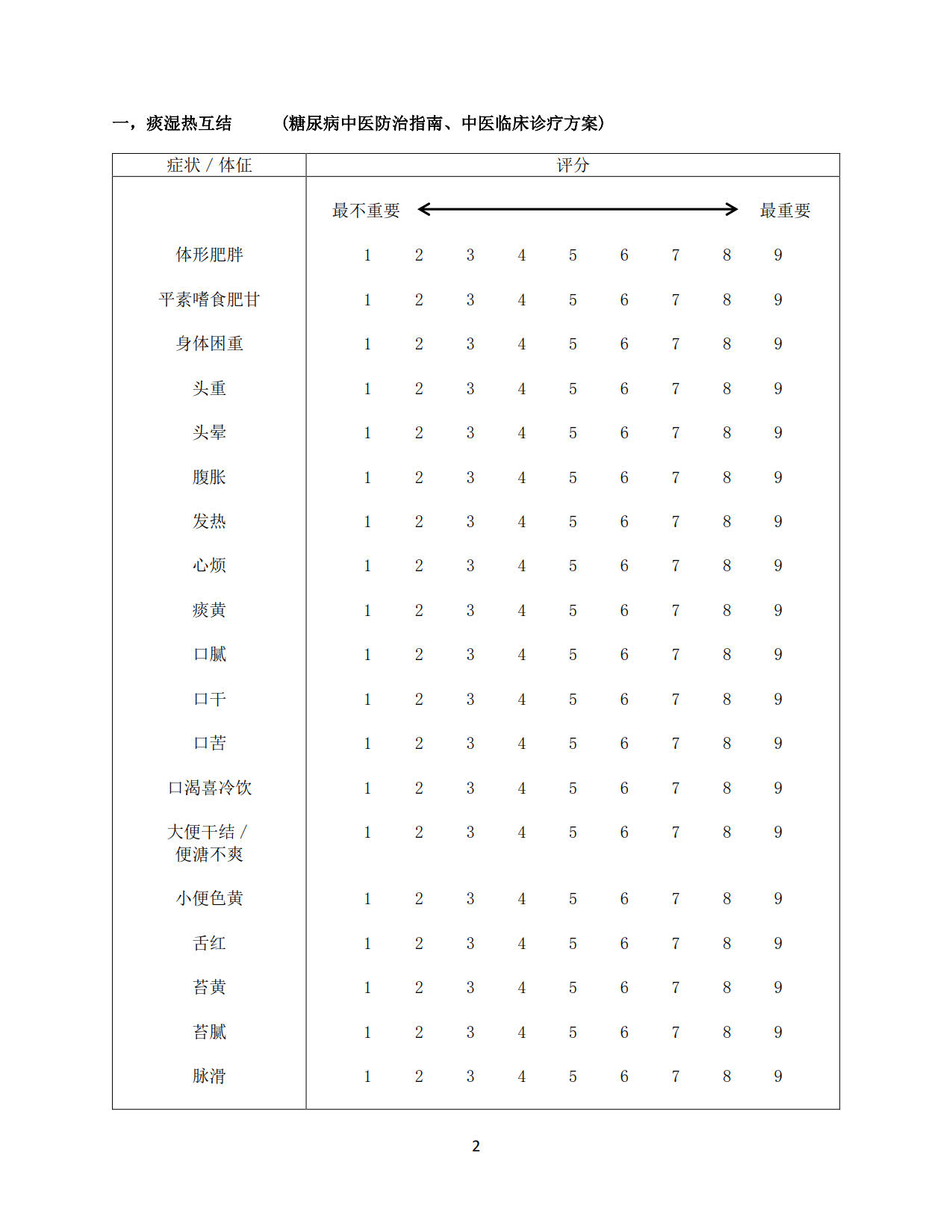

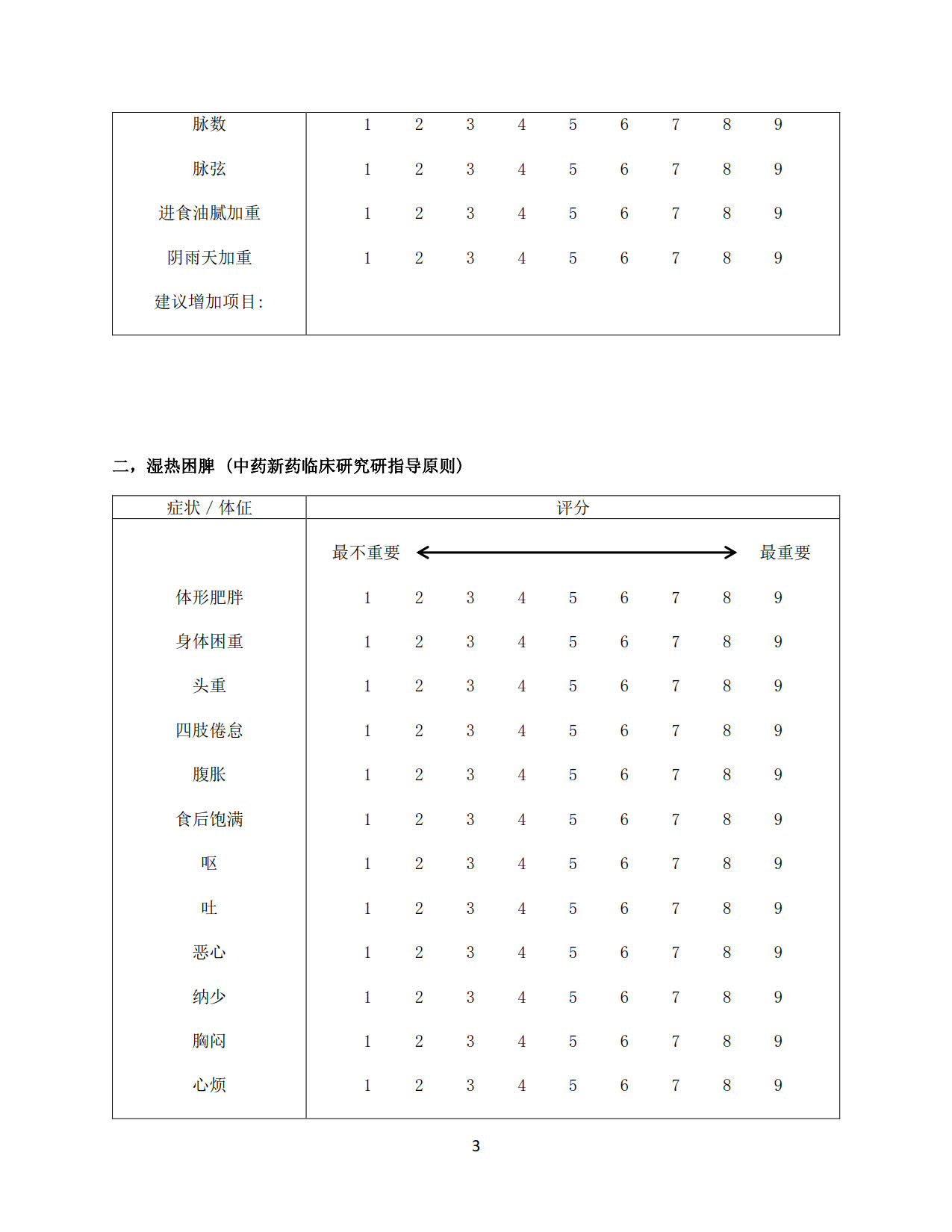

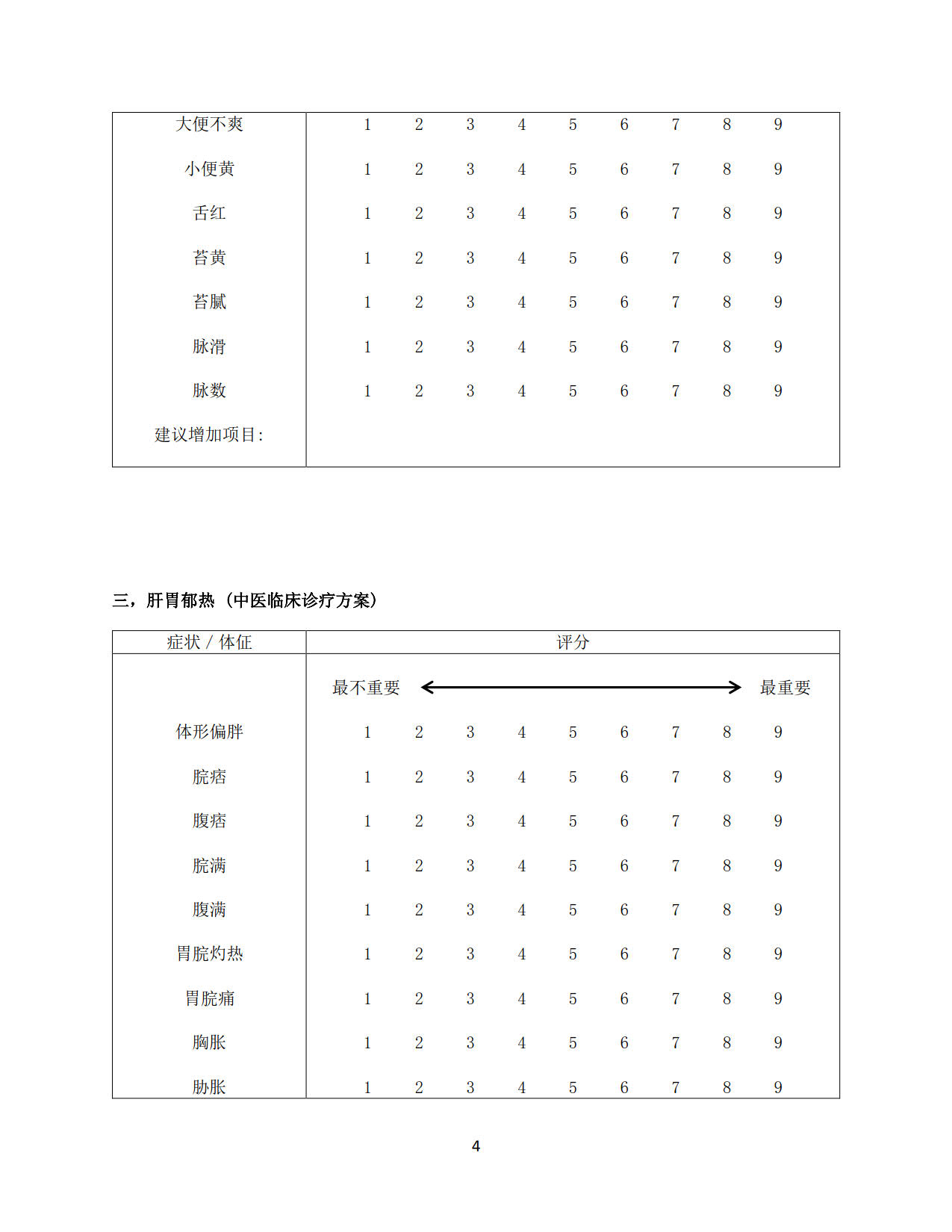

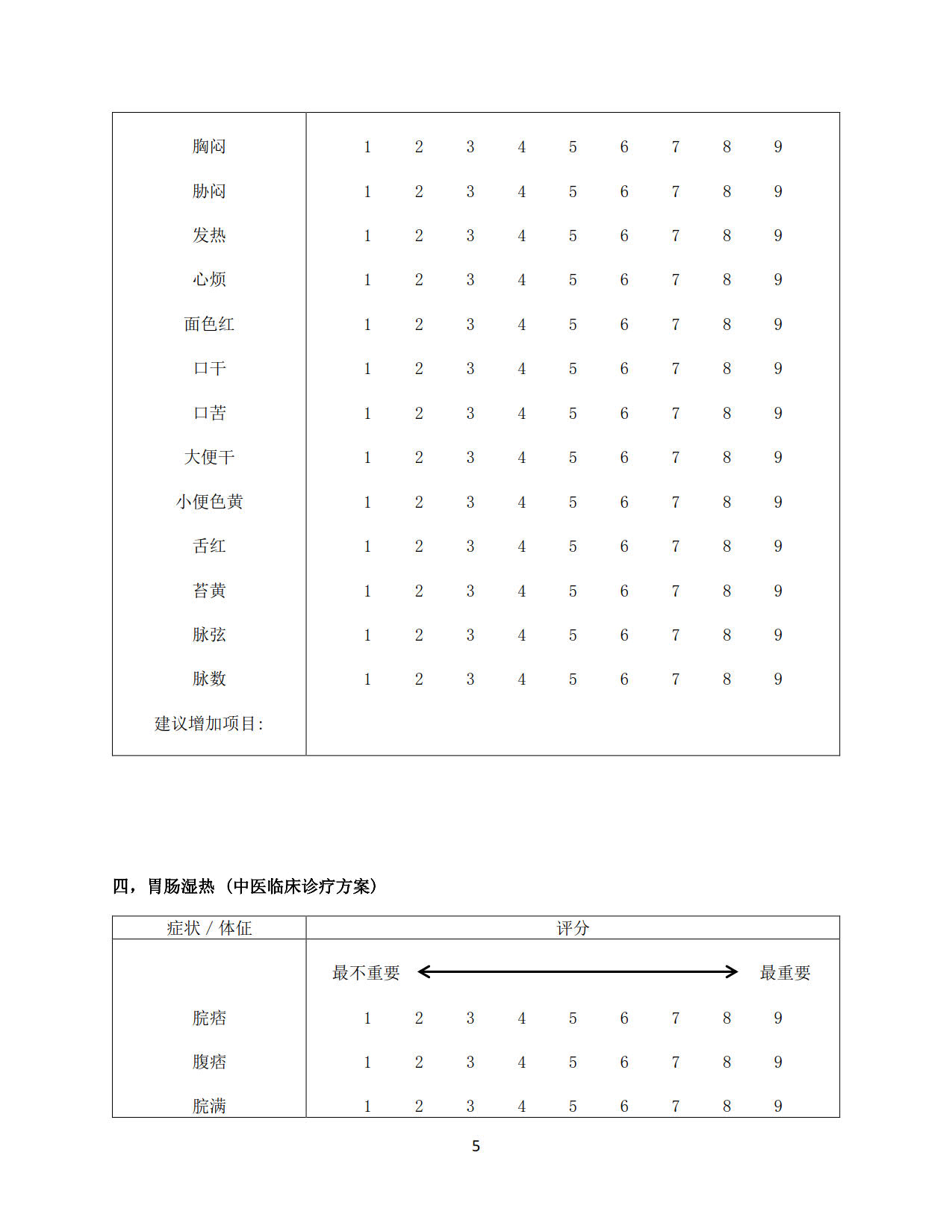

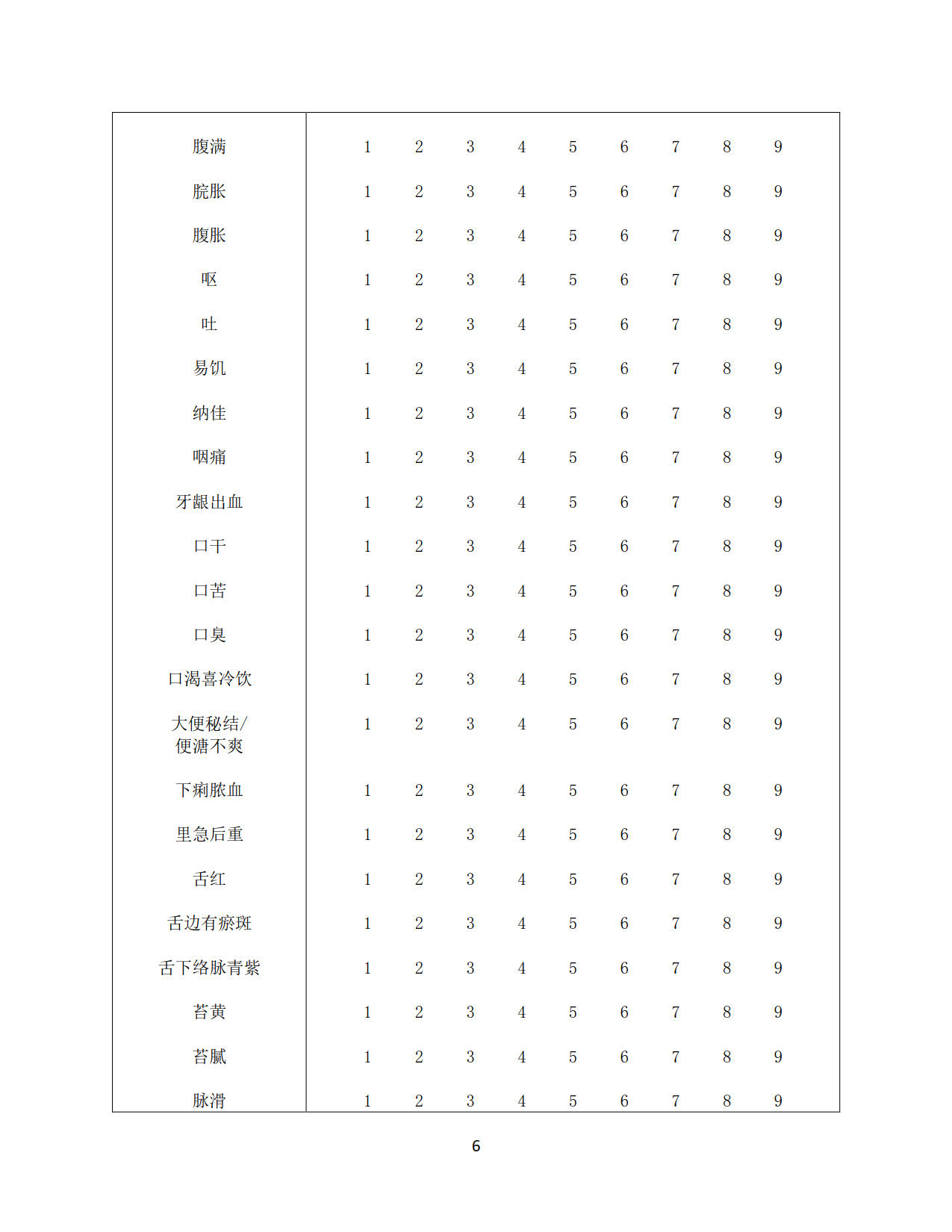

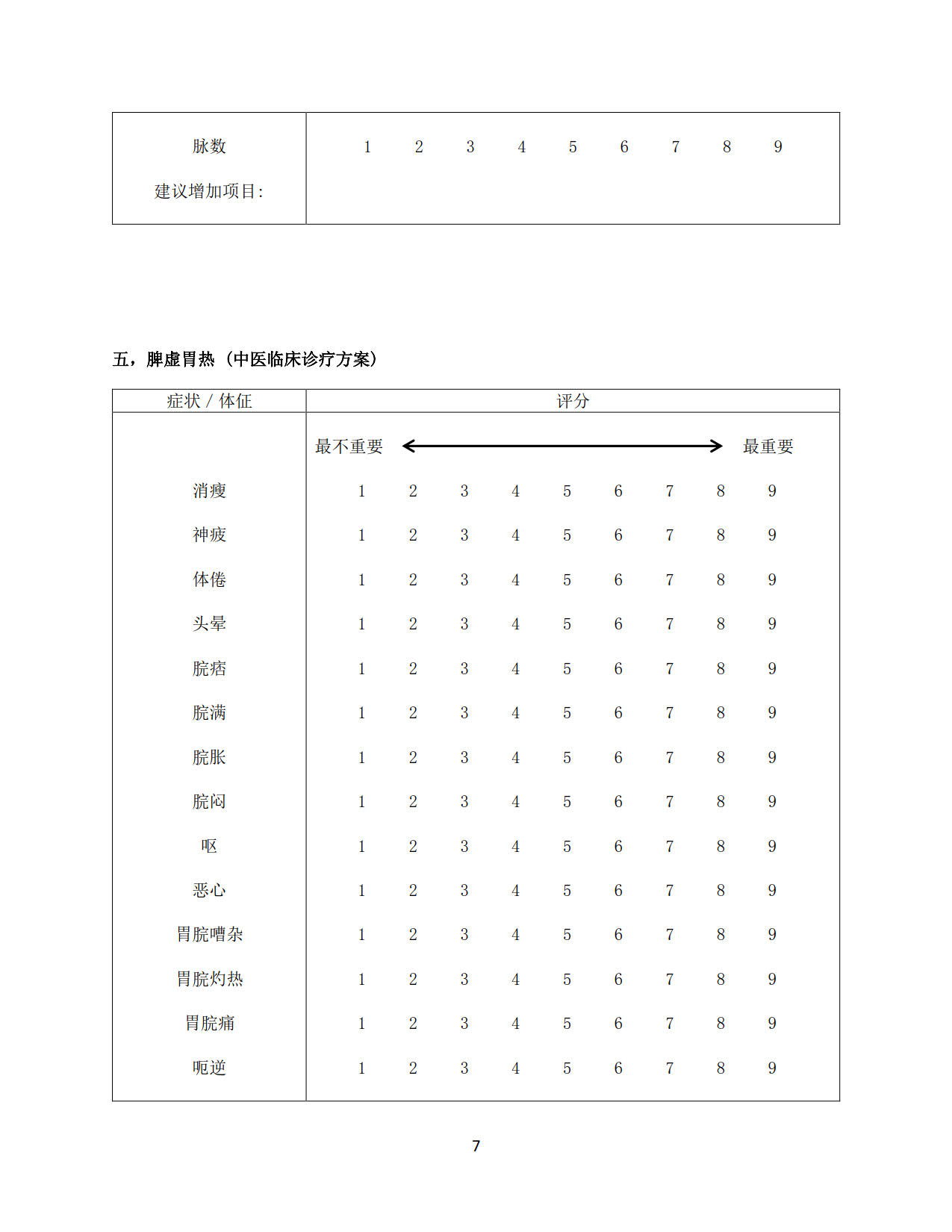

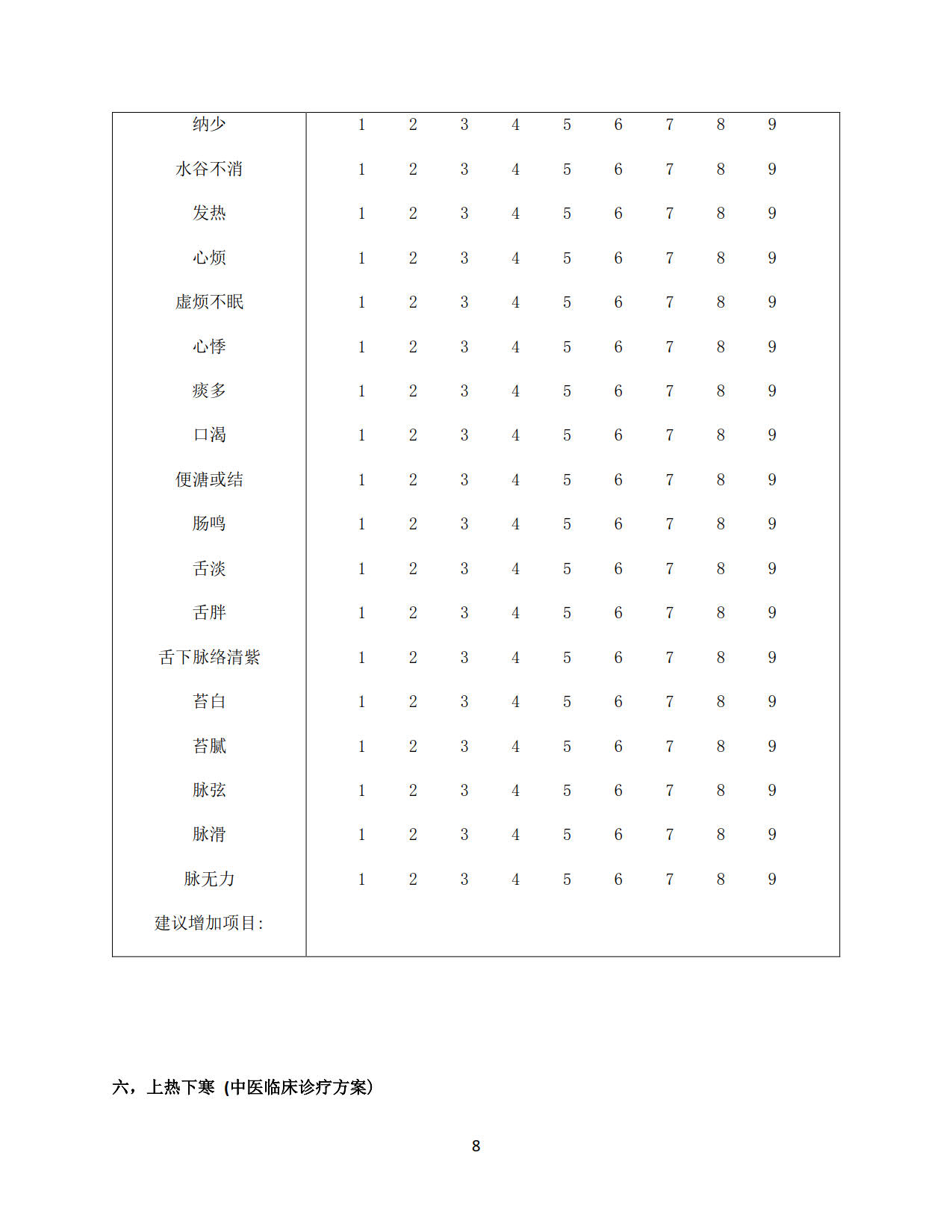

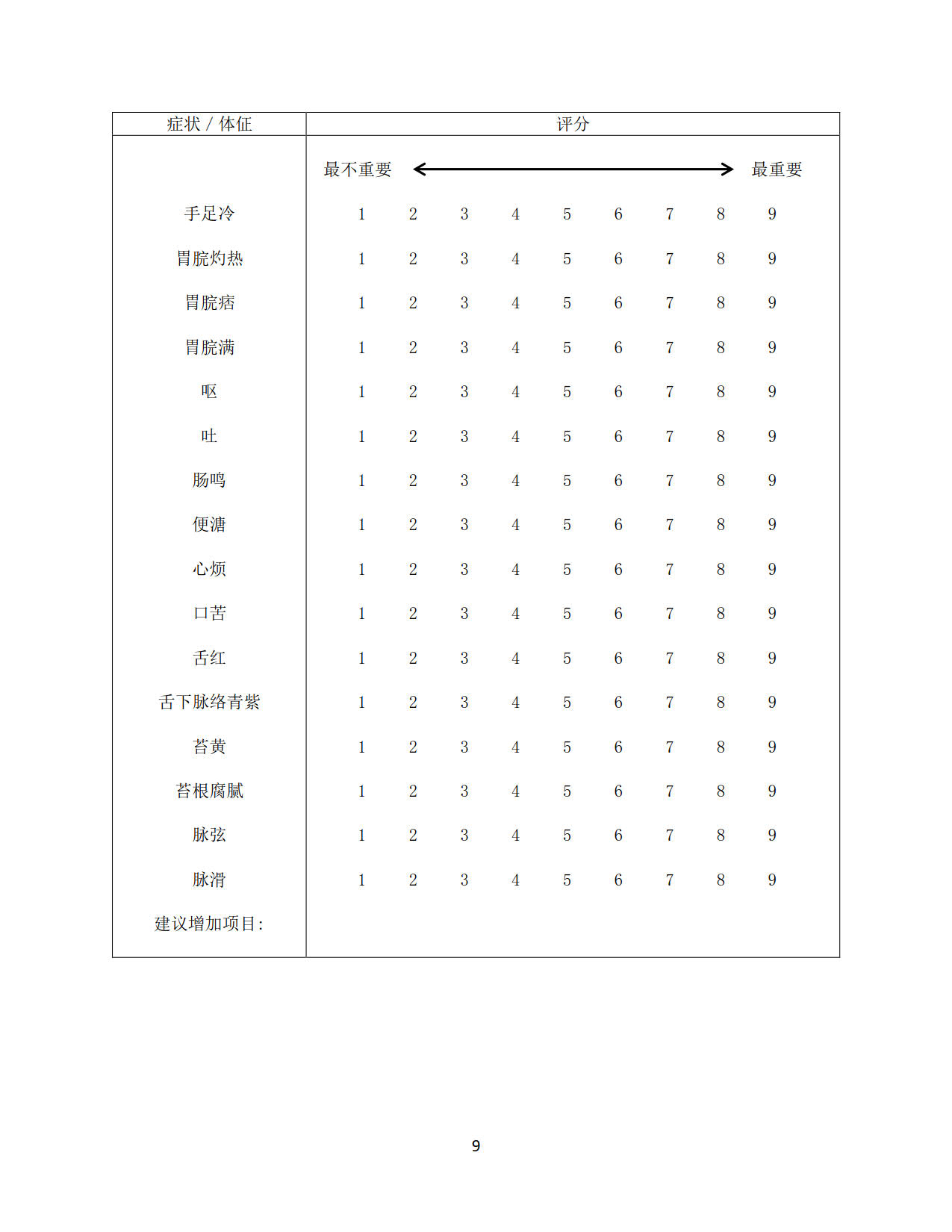

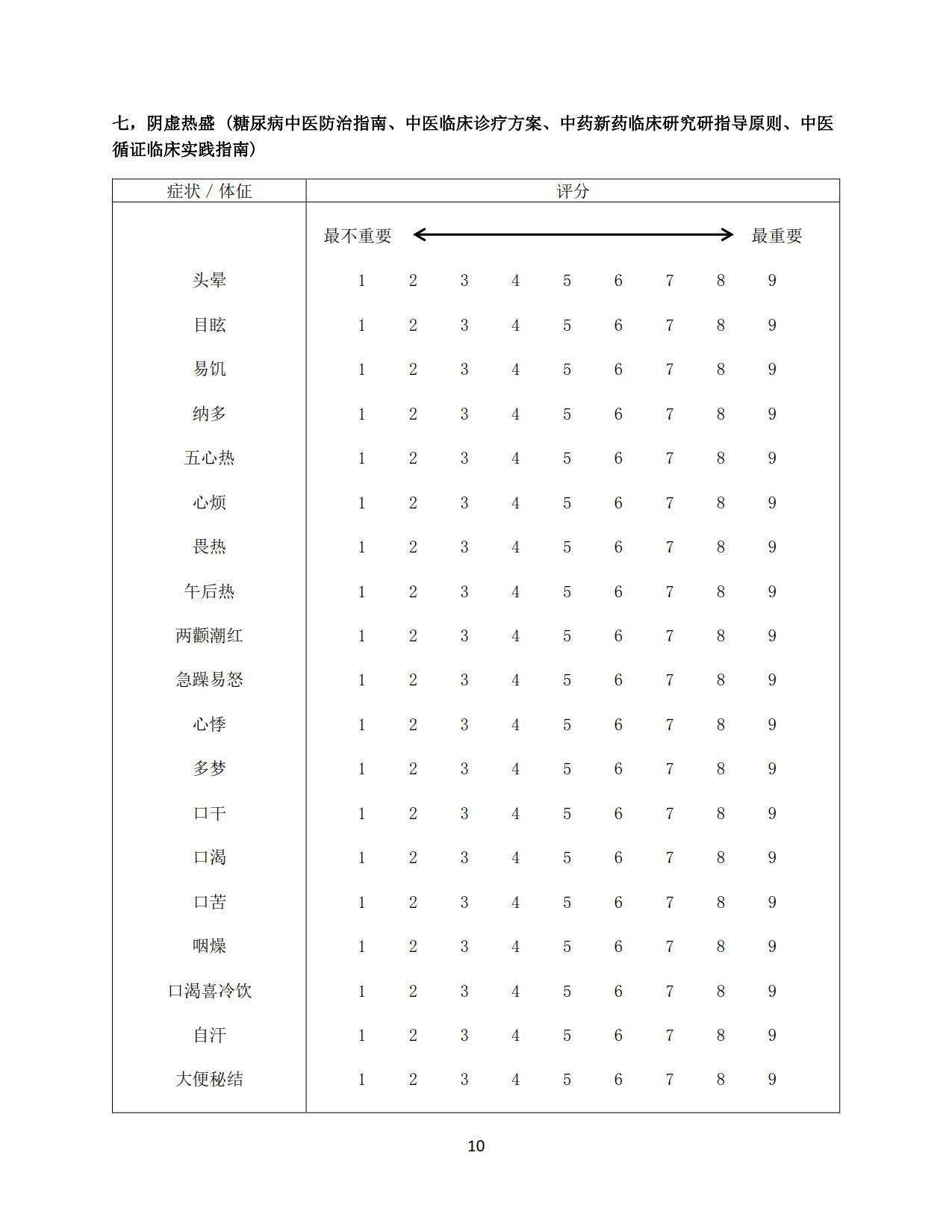

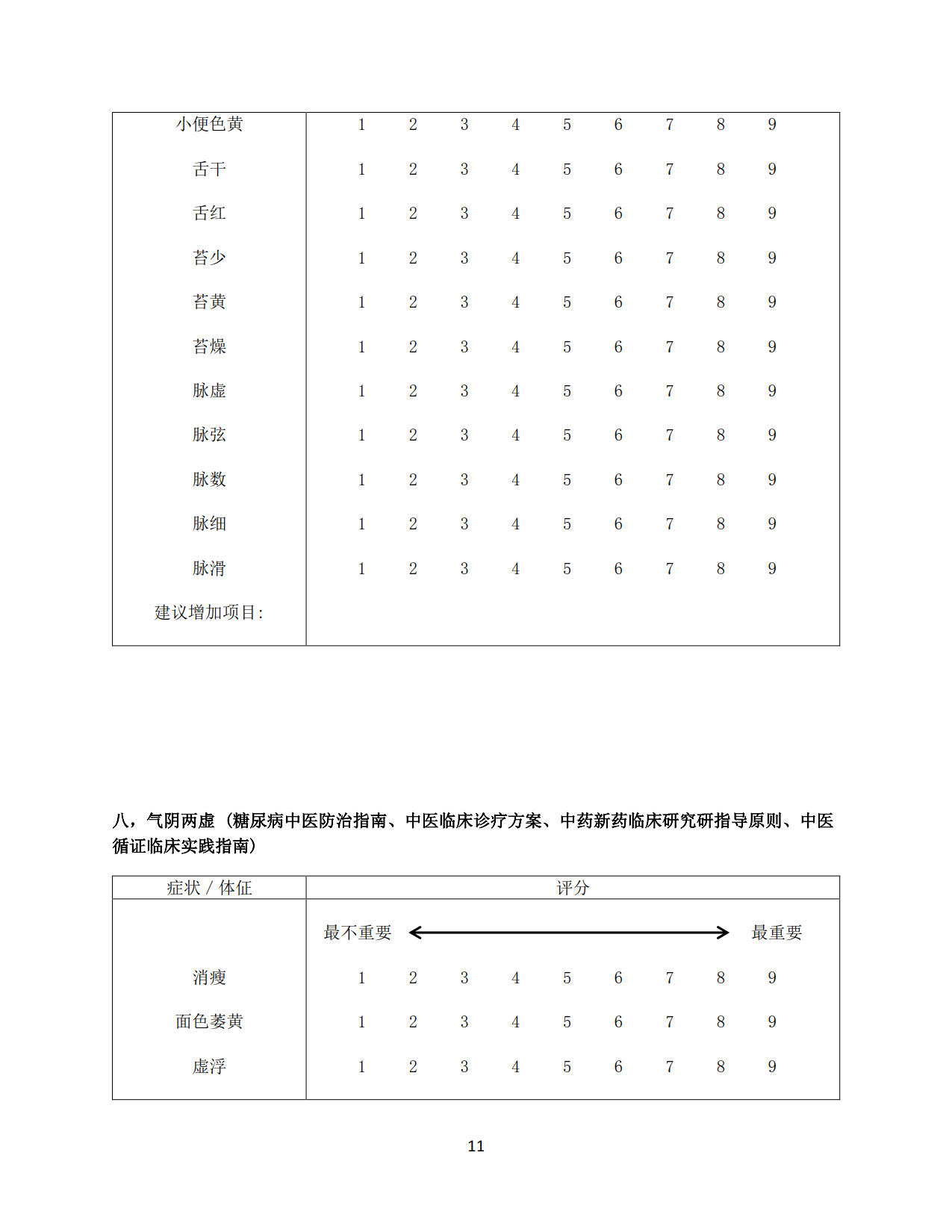

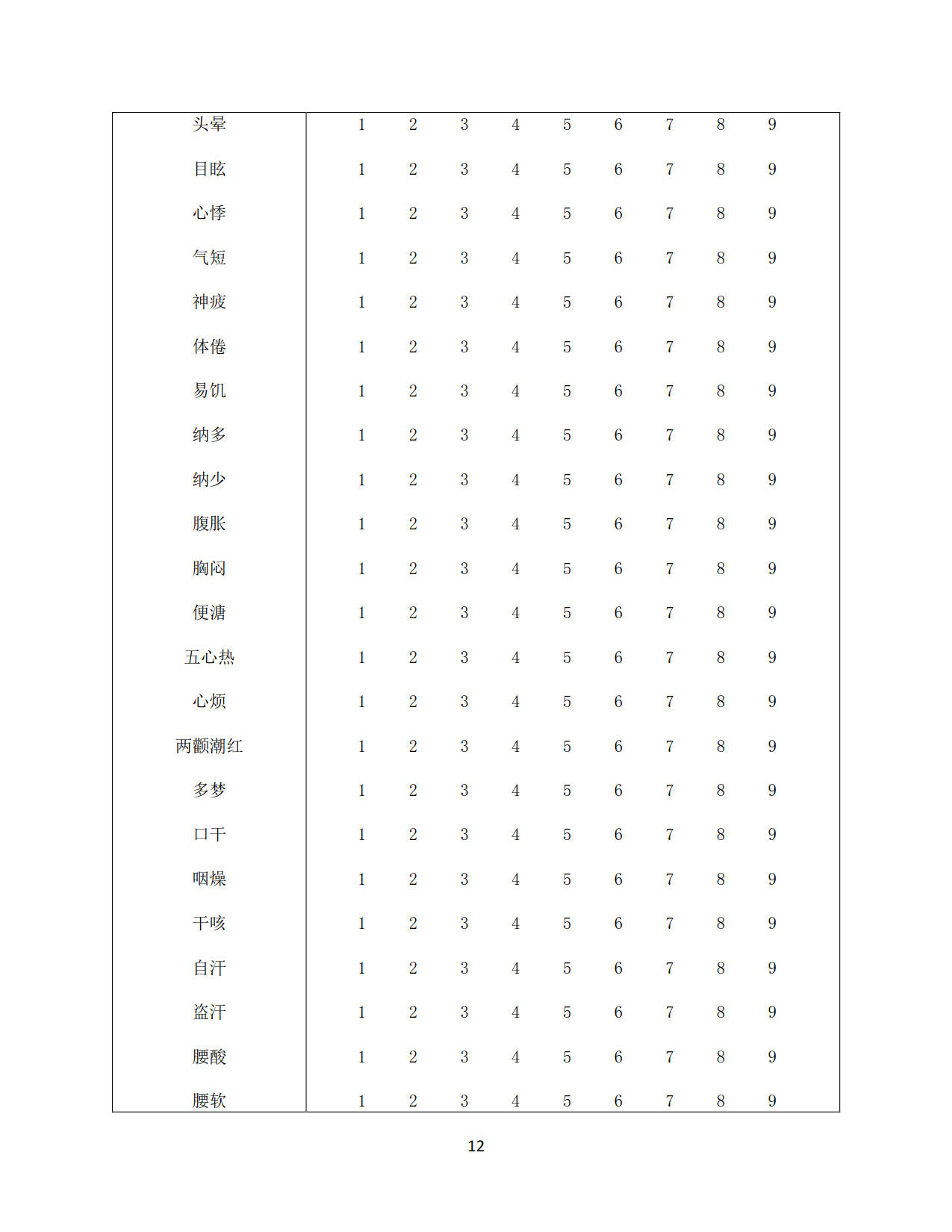

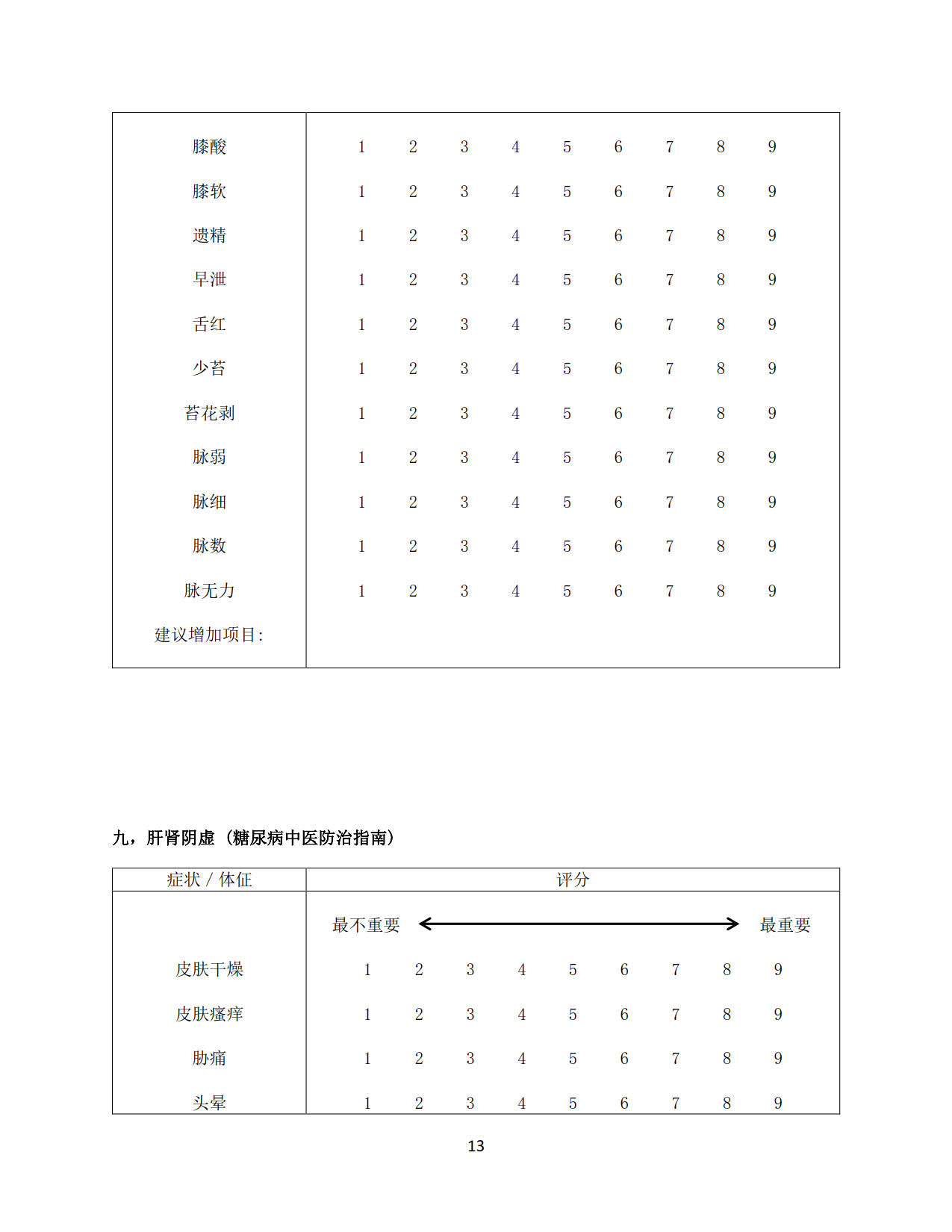

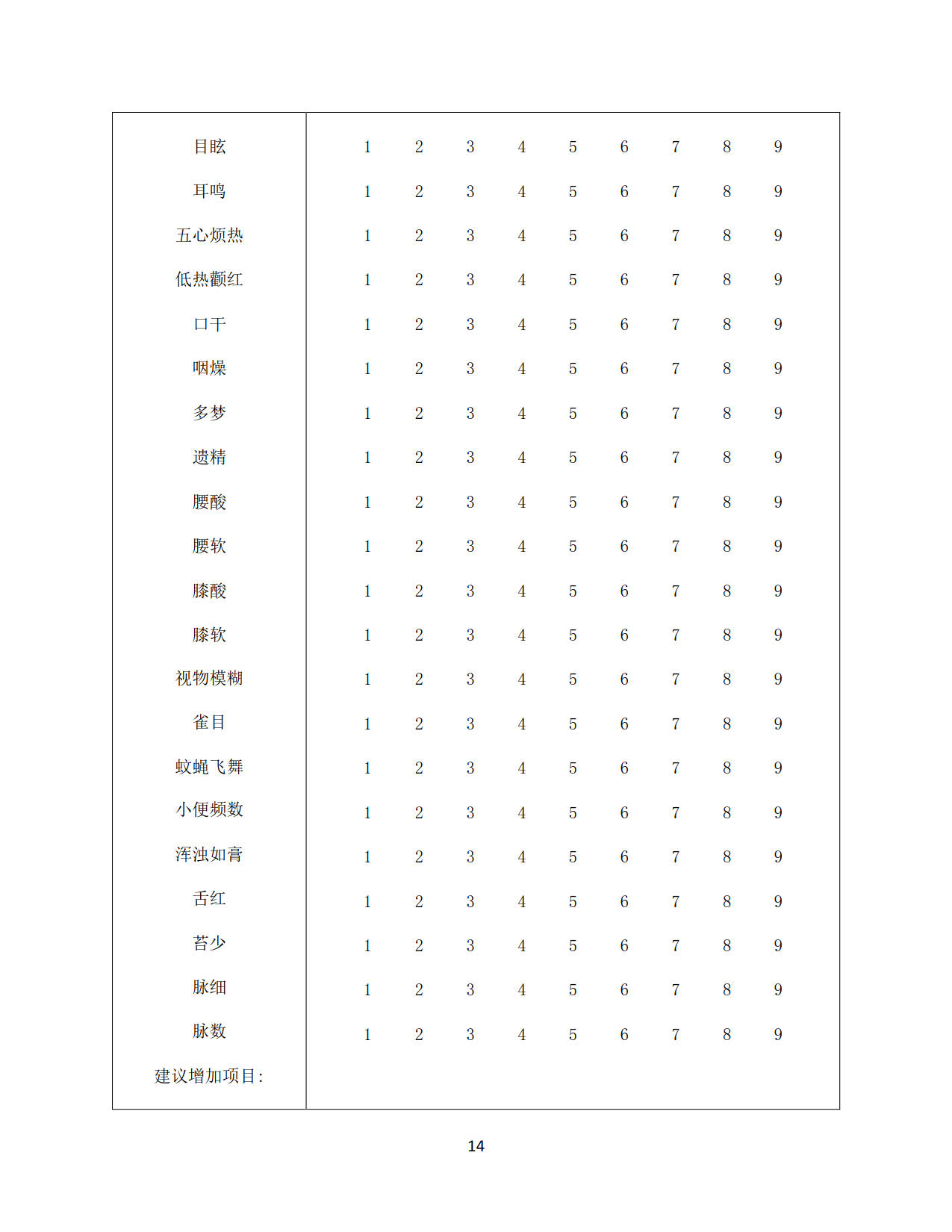

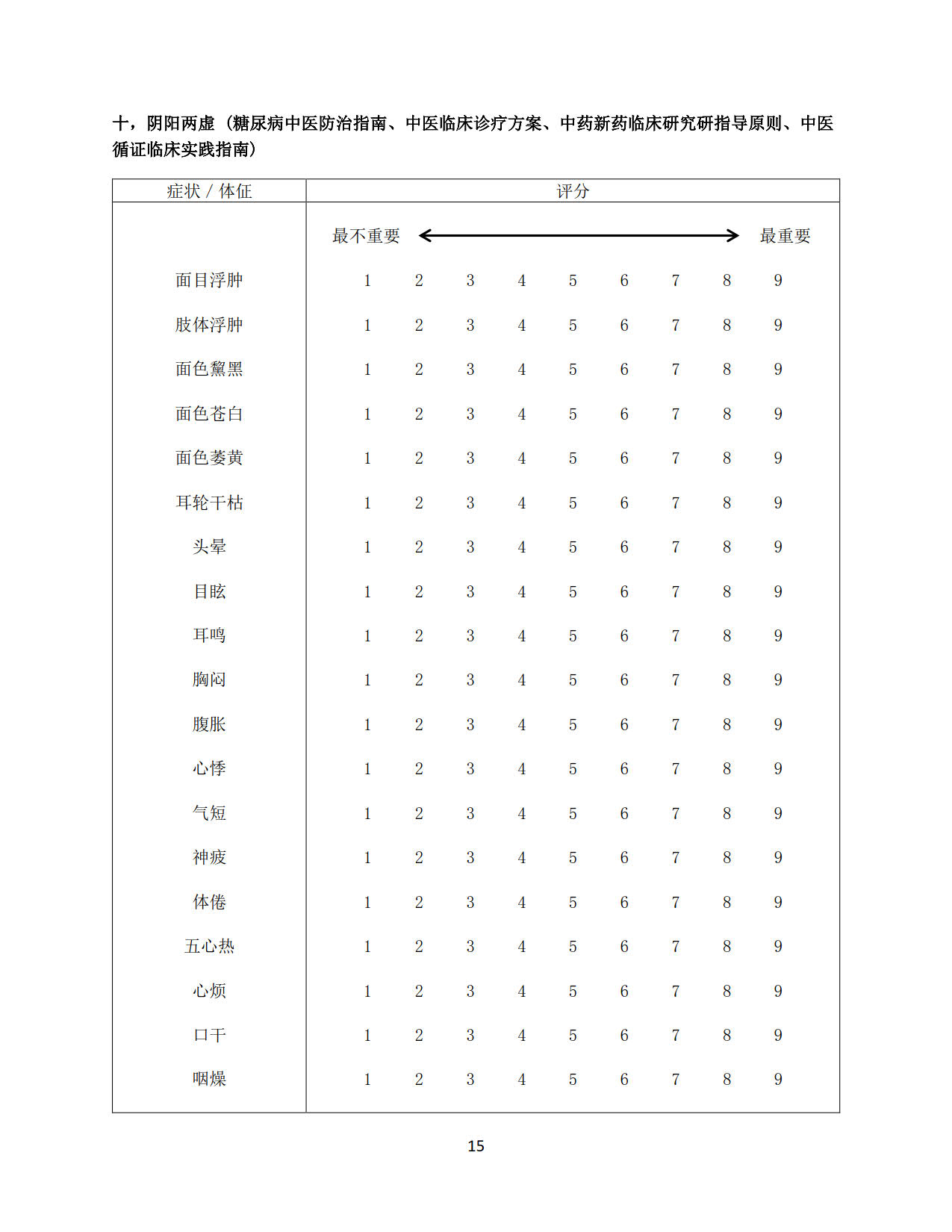

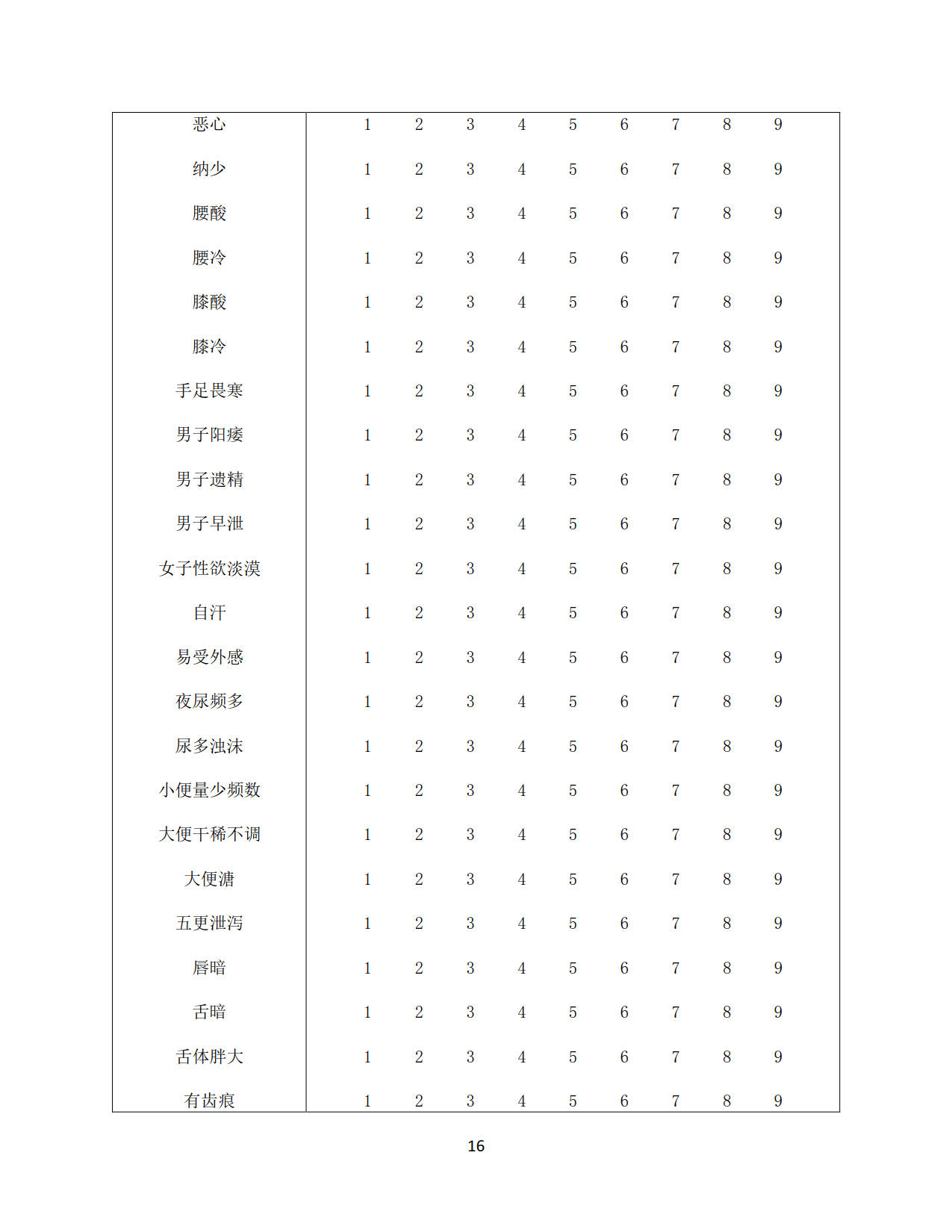

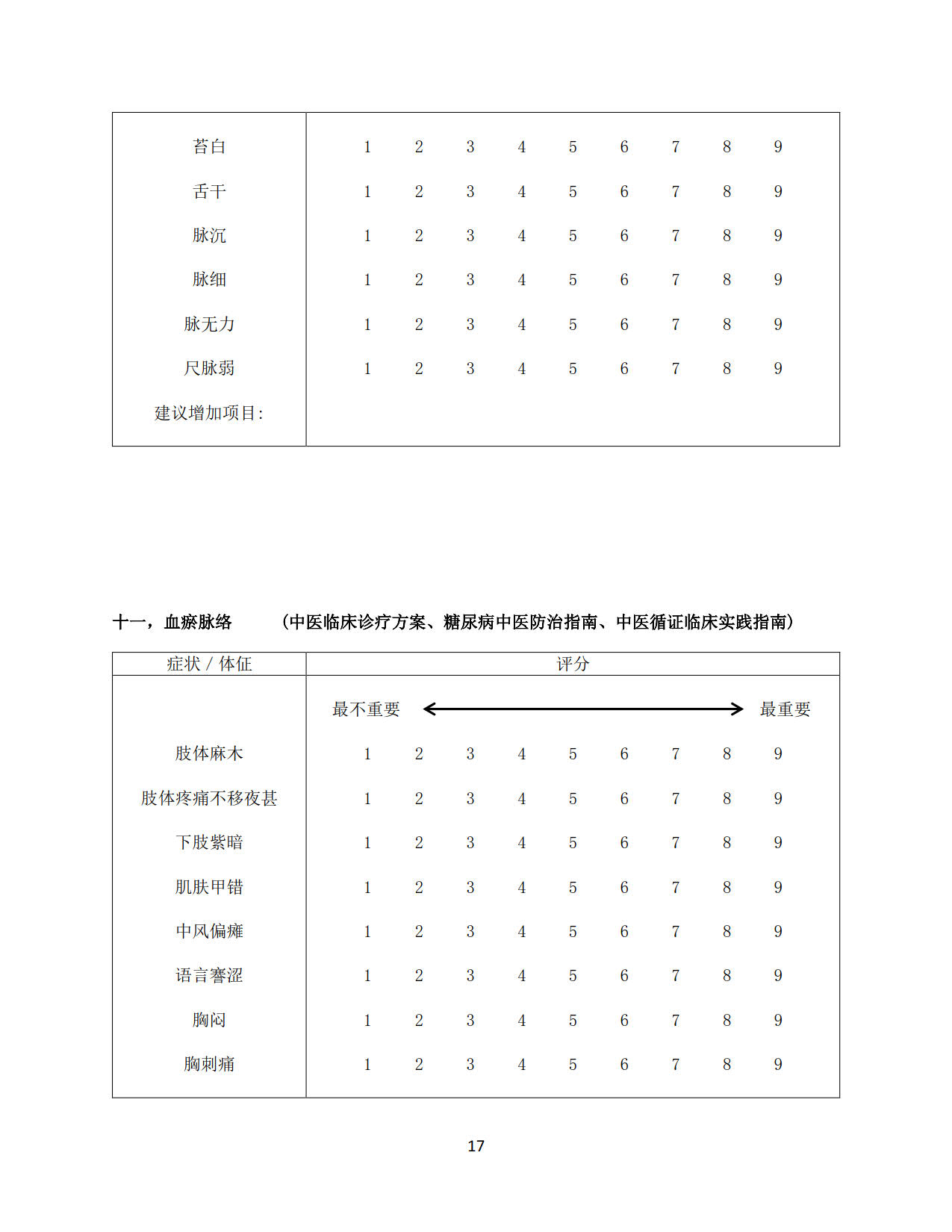

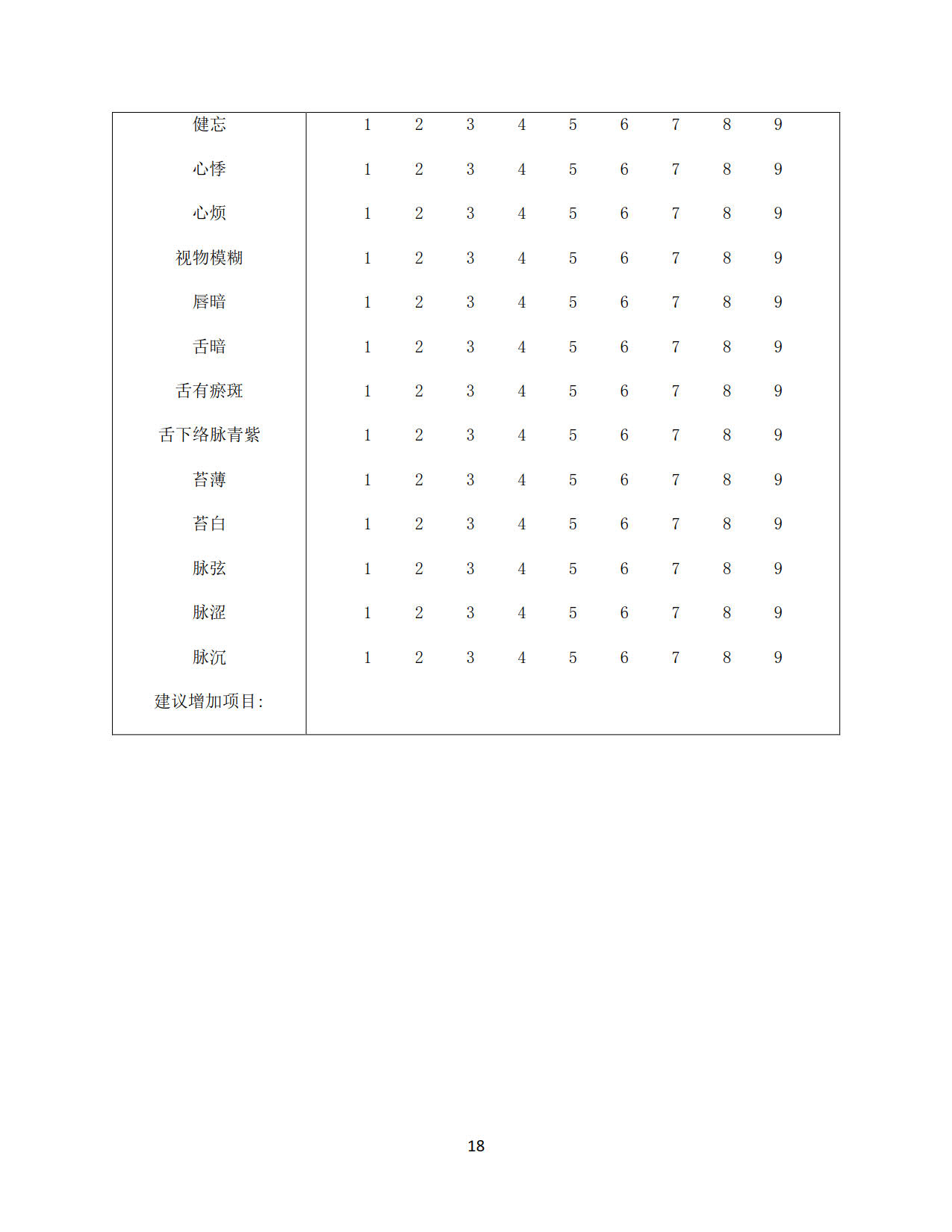


**Round 2**


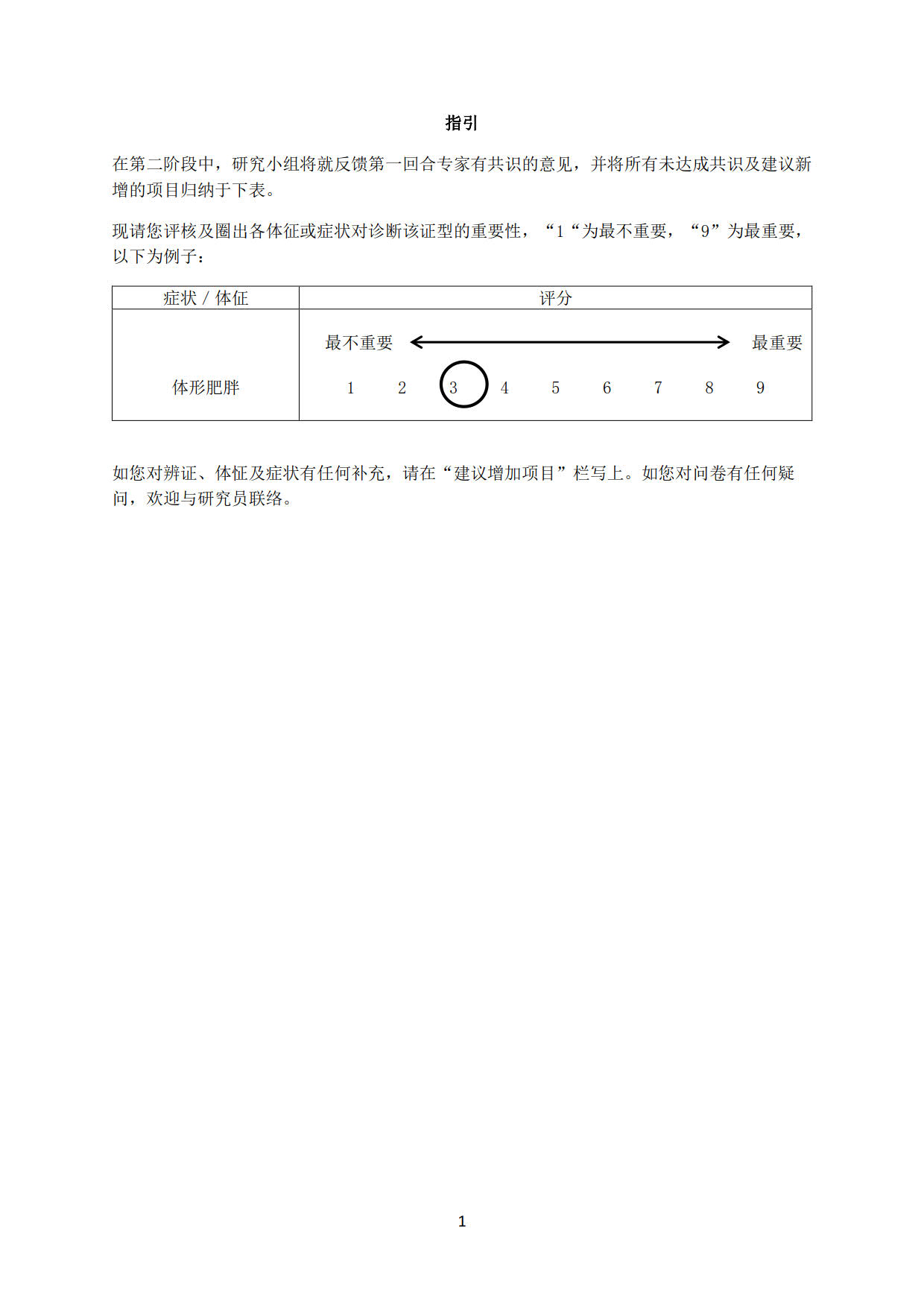

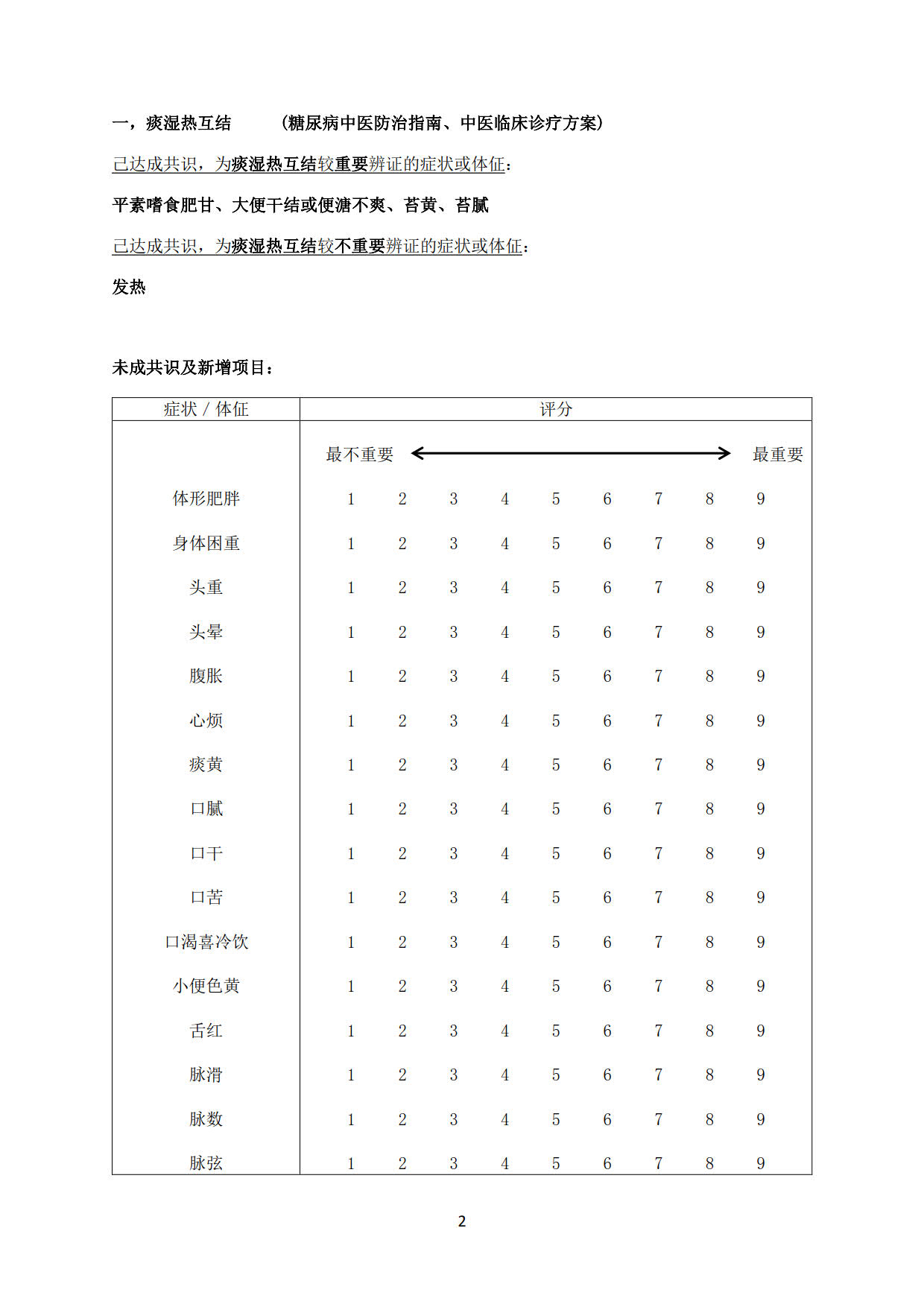

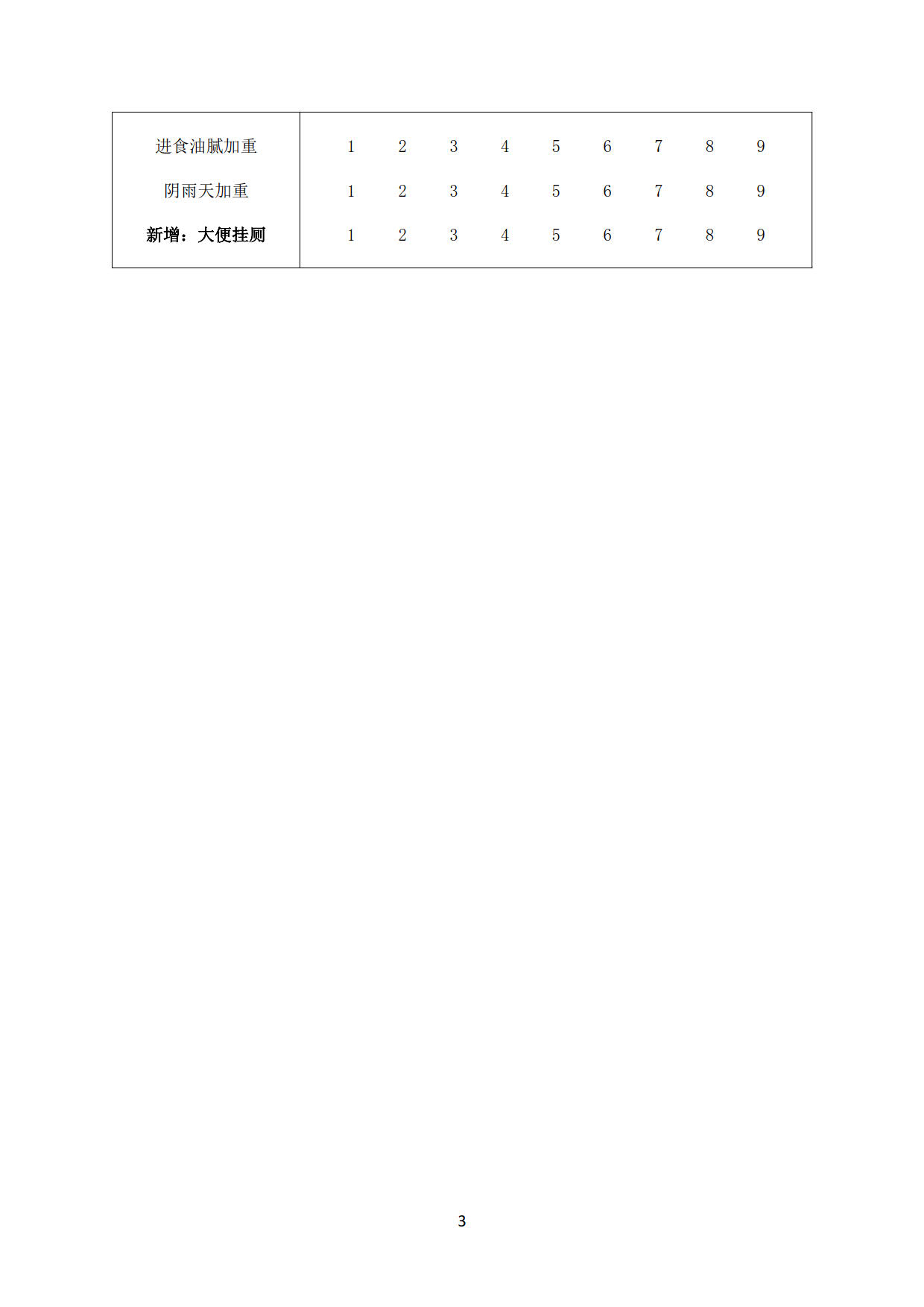

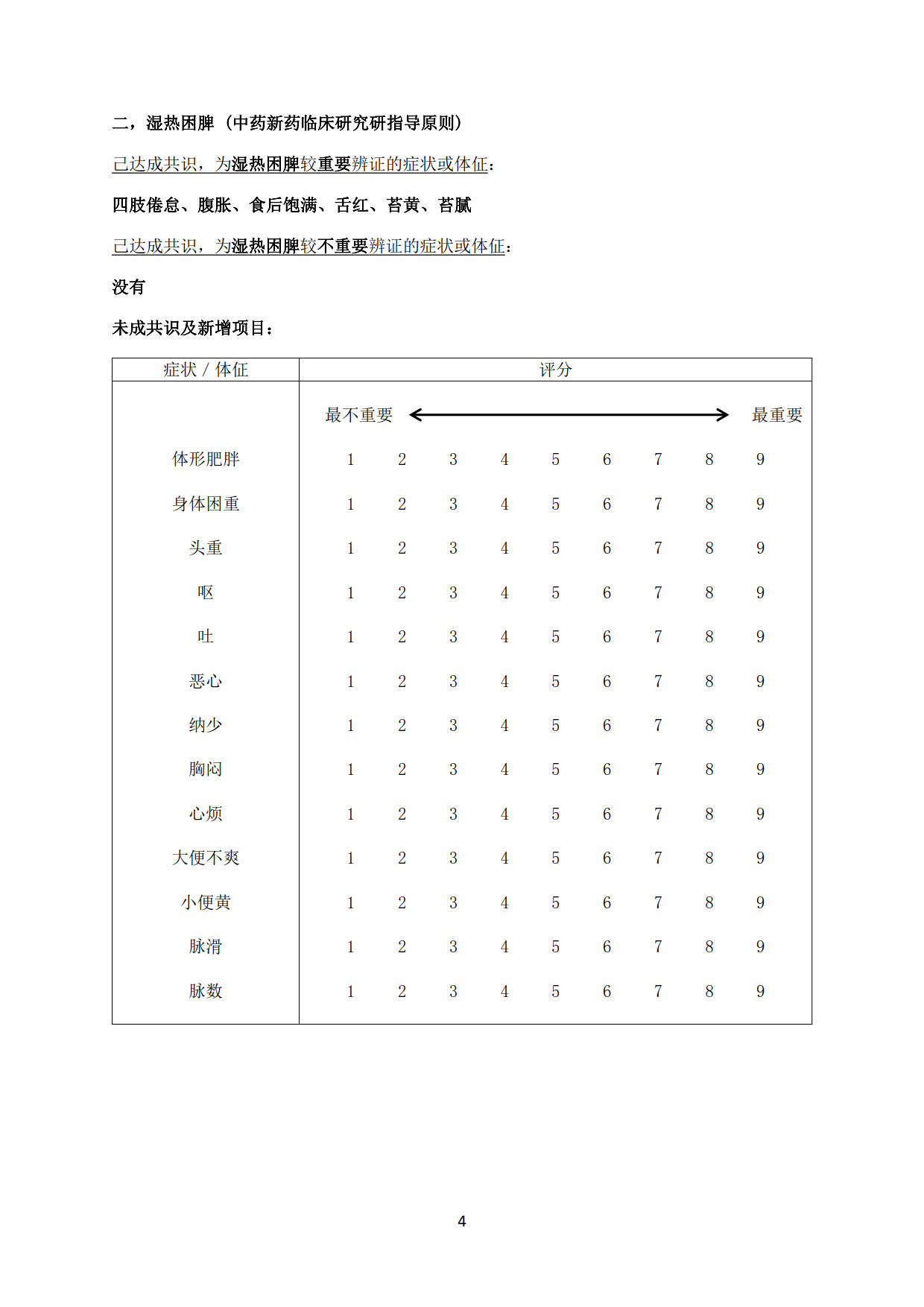

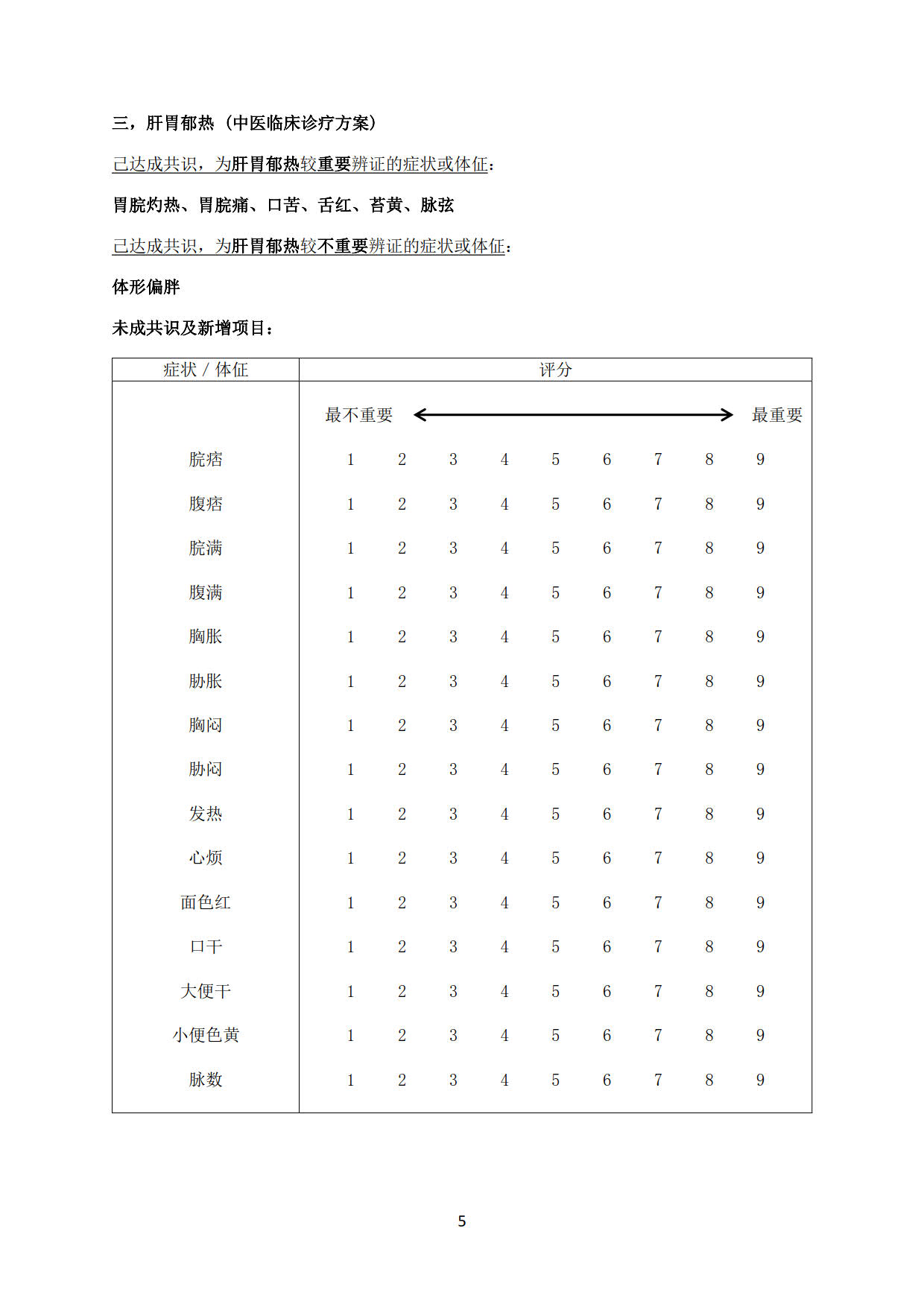

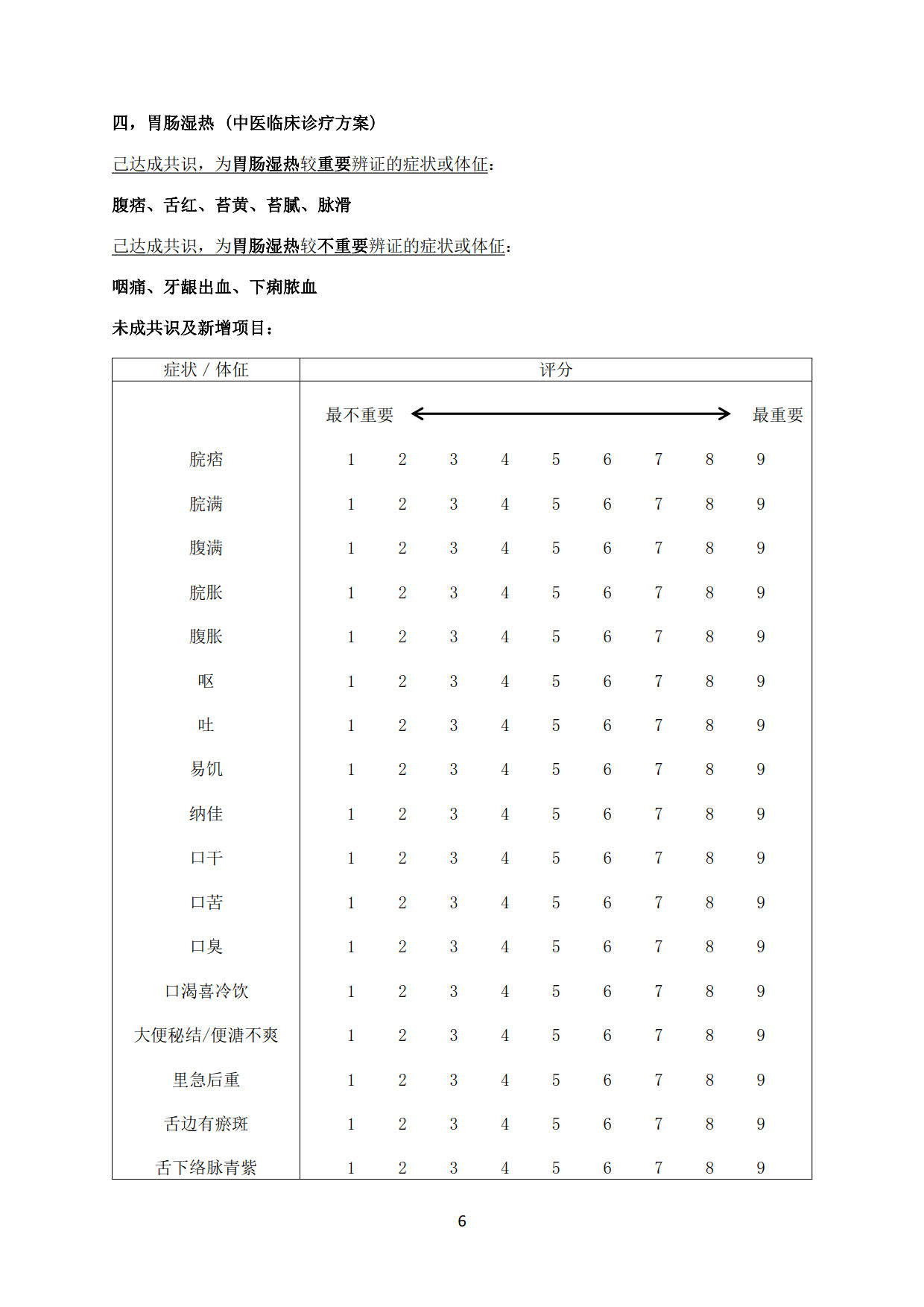

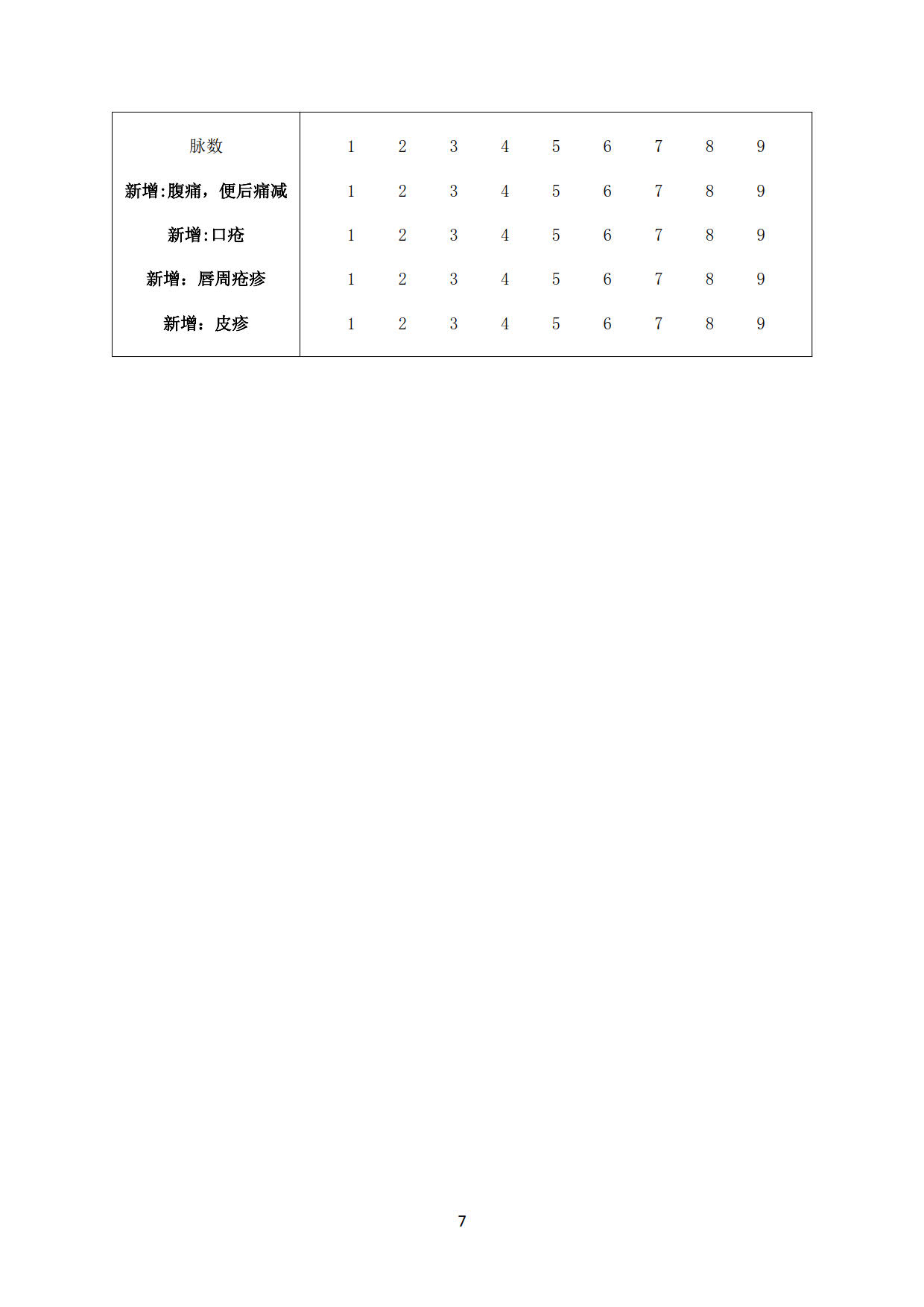

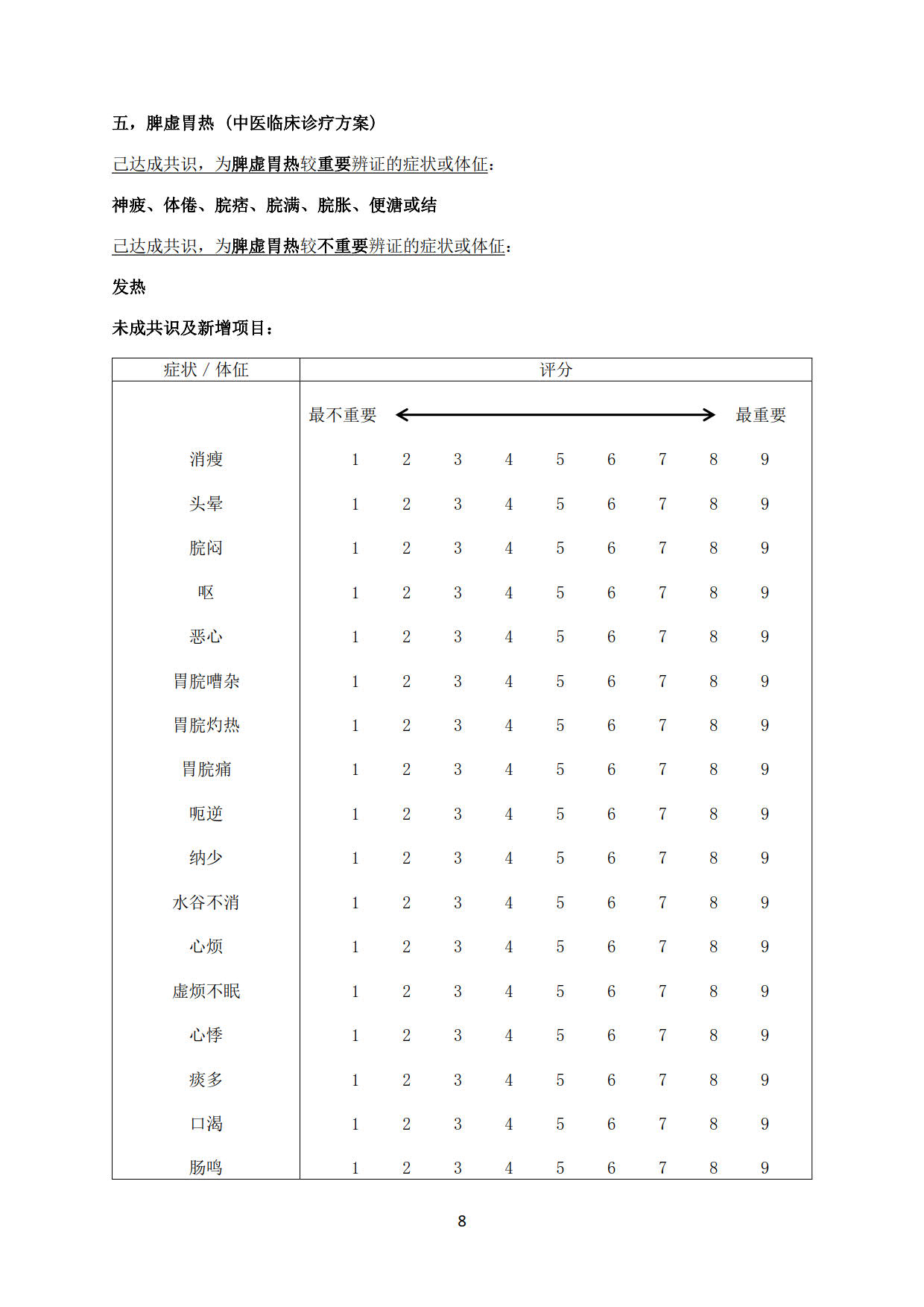

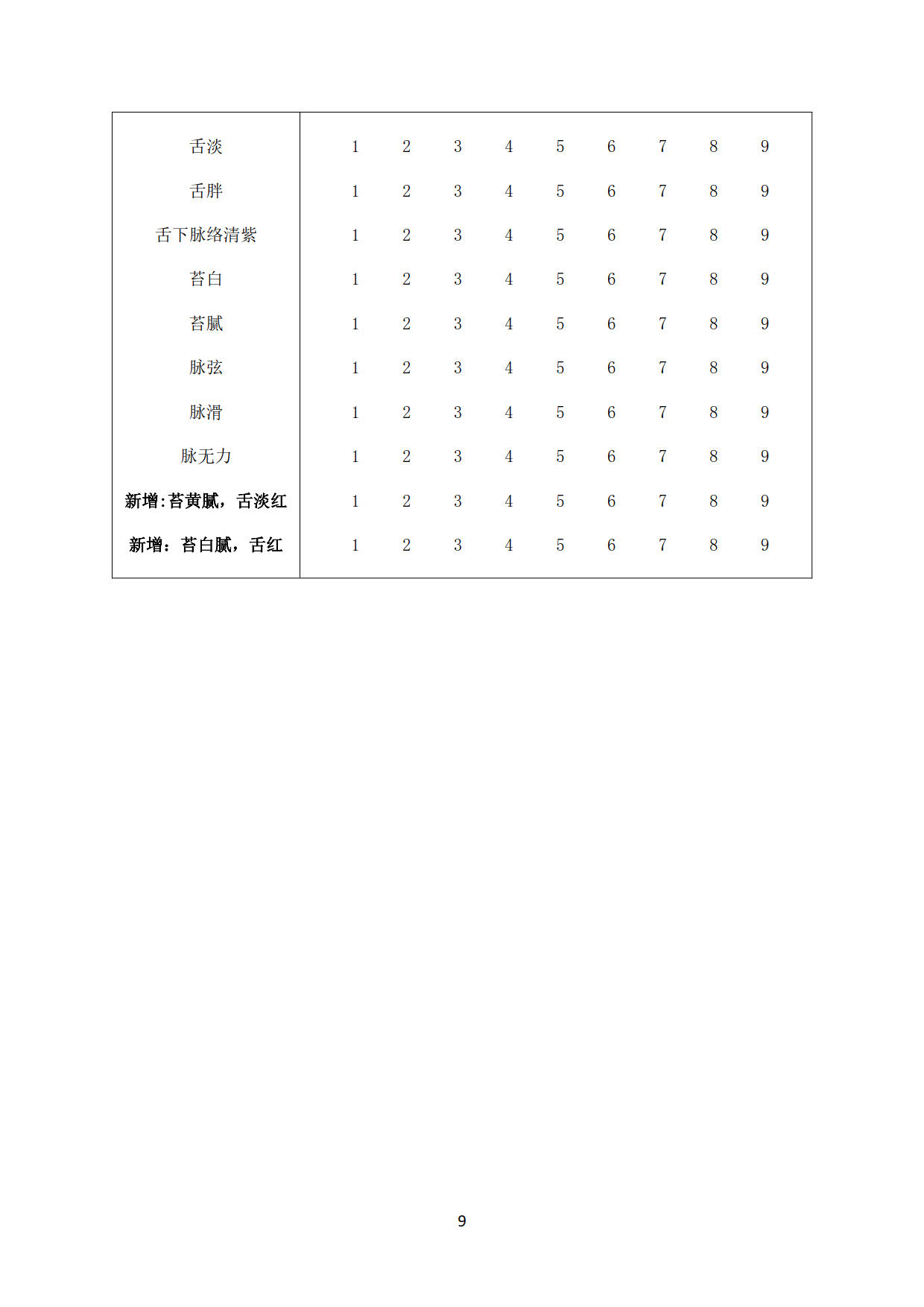

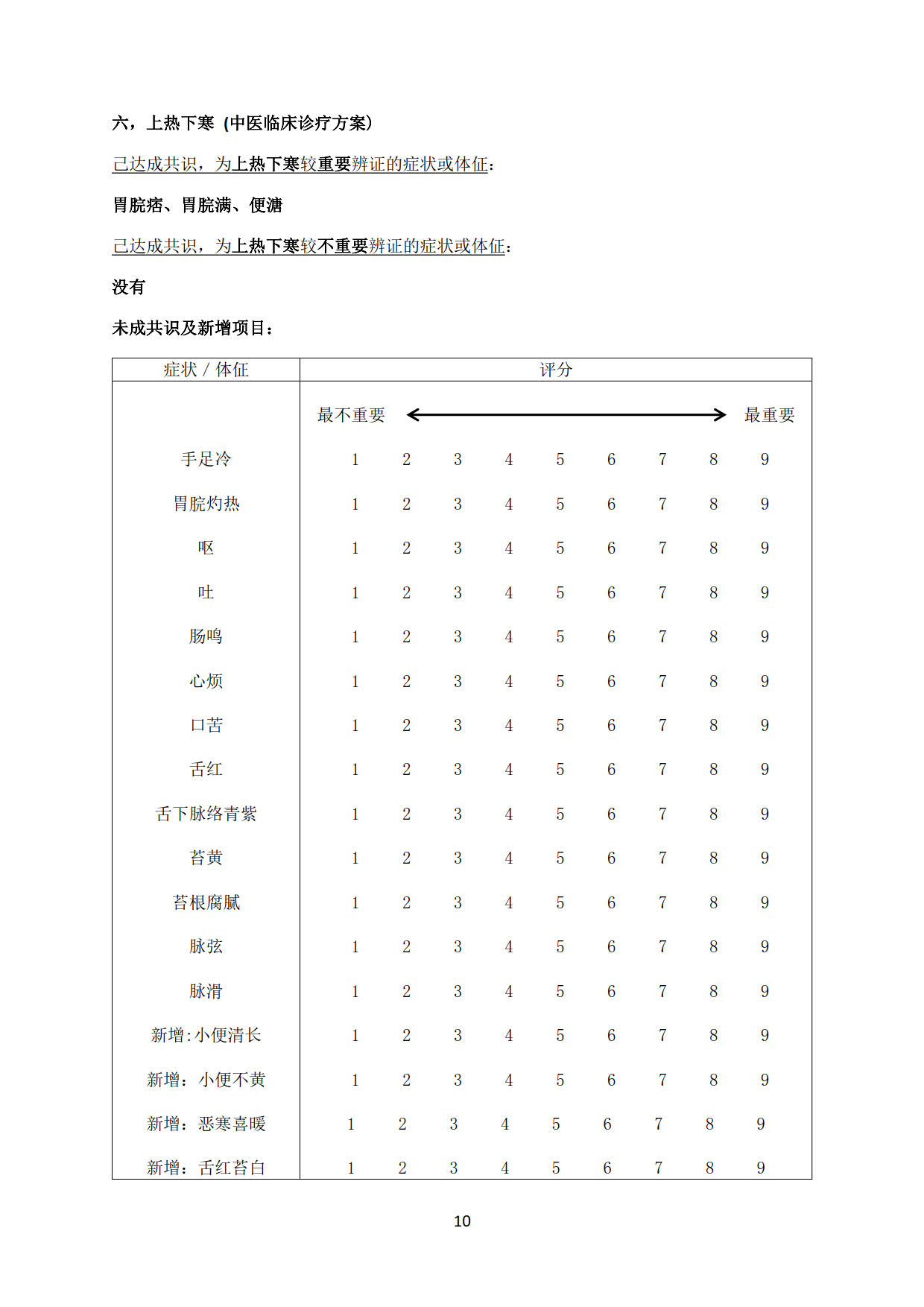

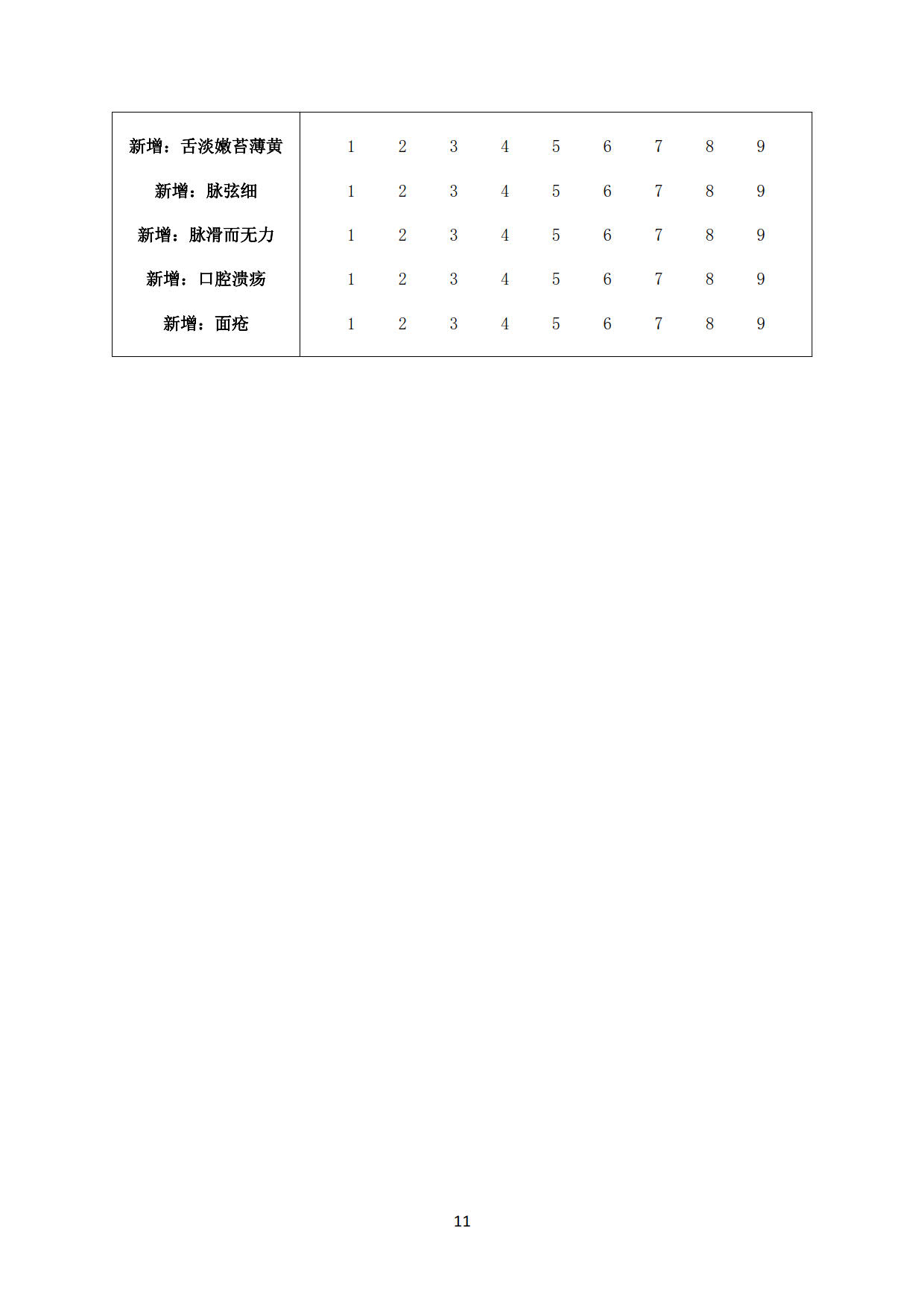

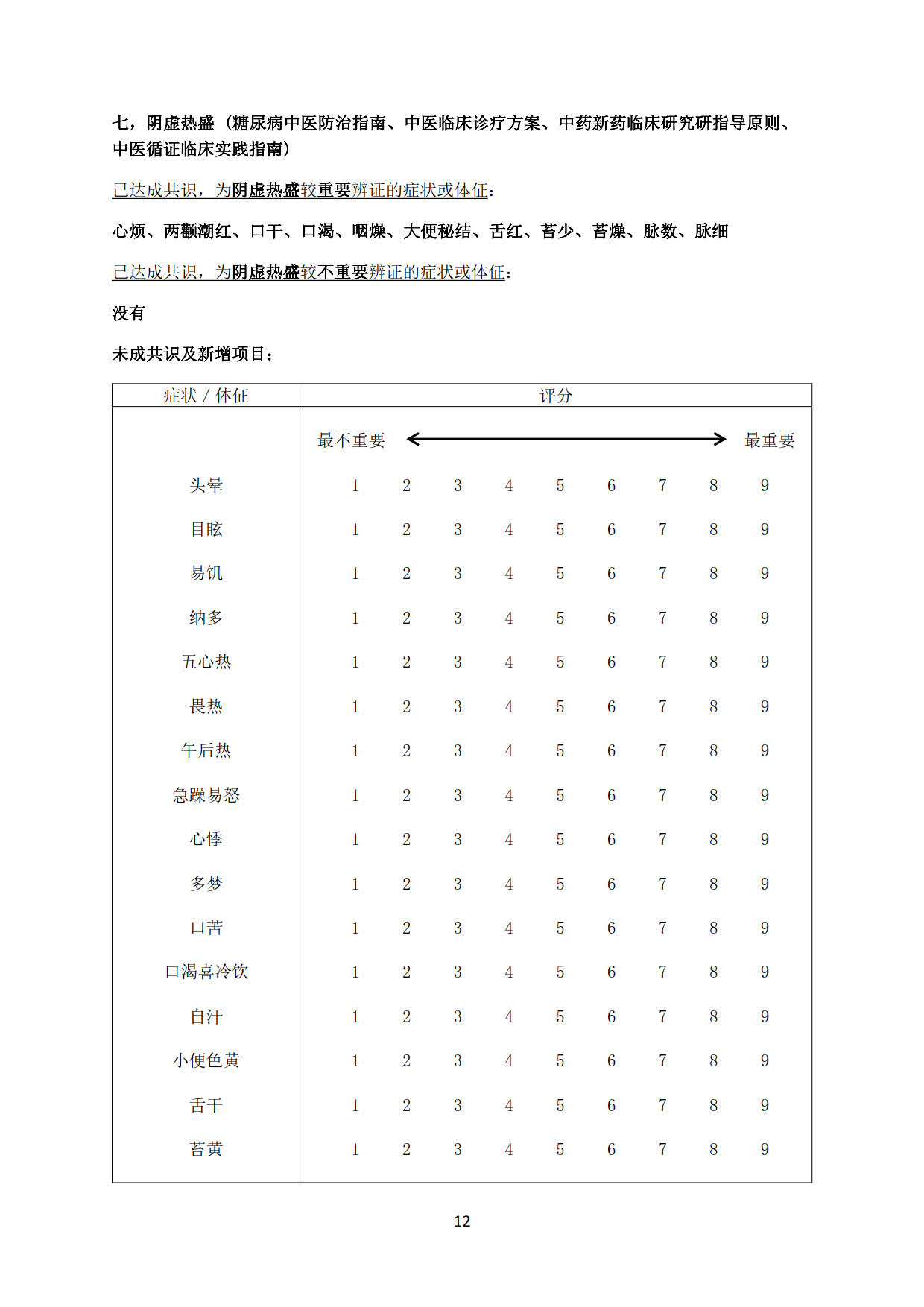

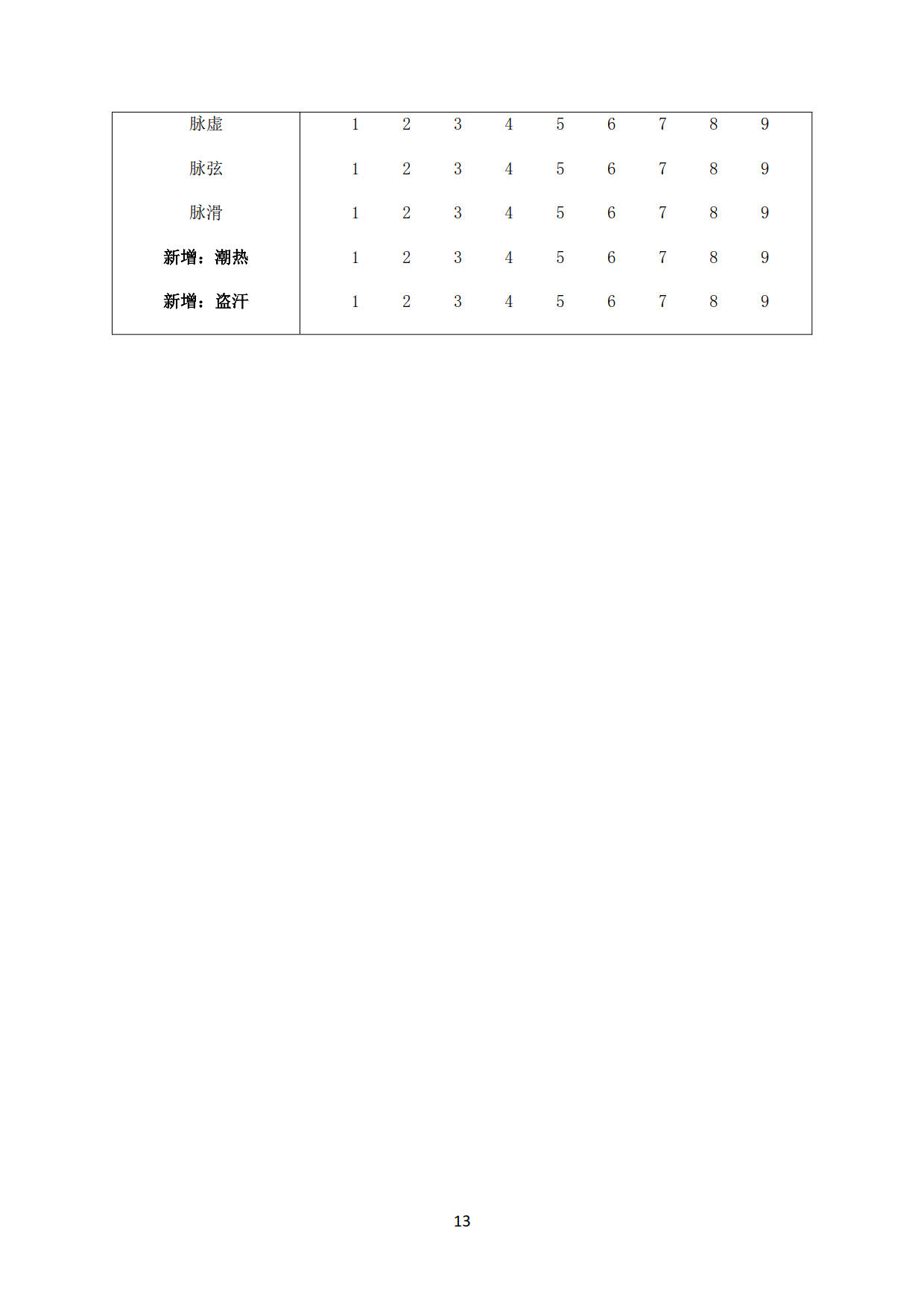

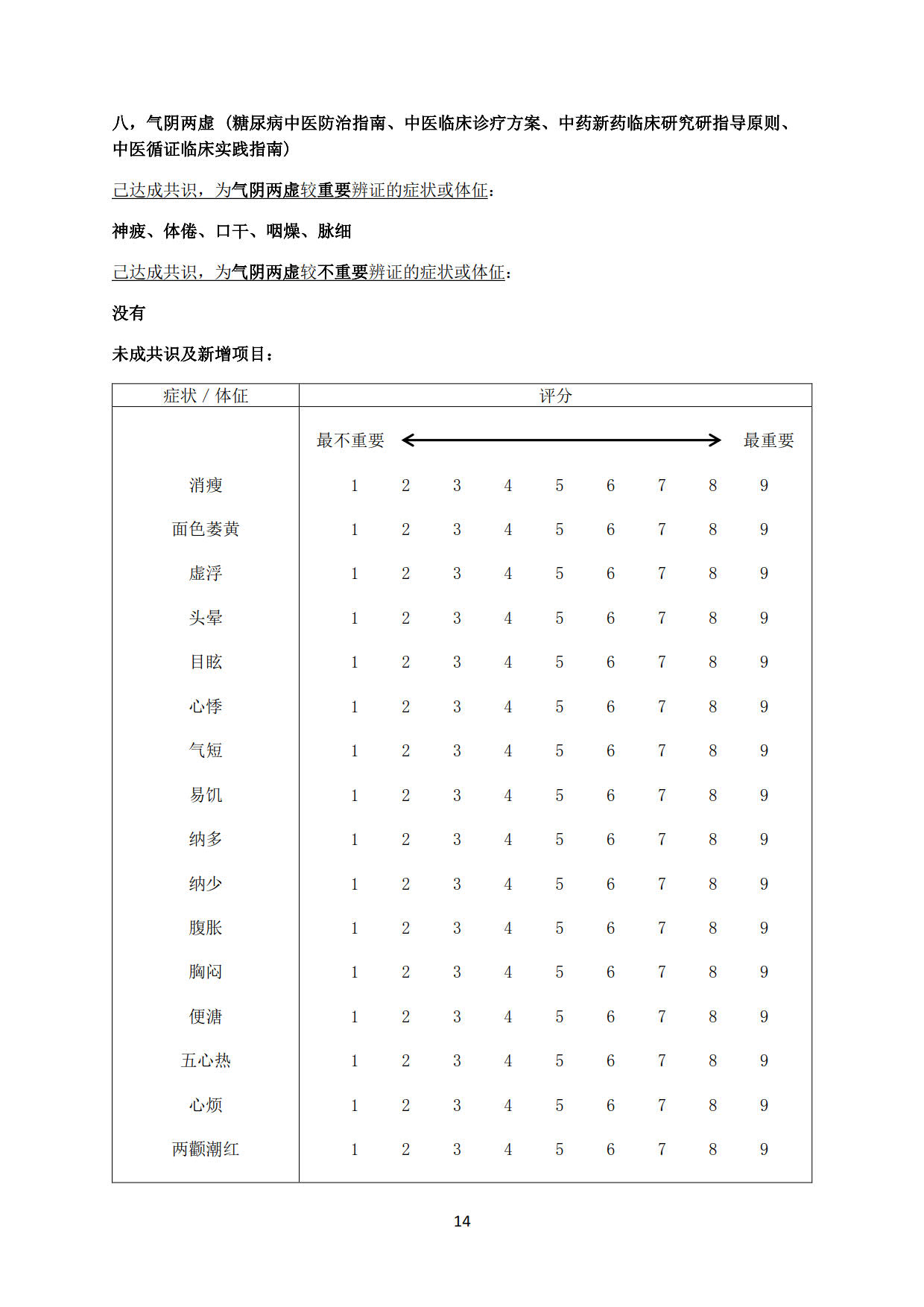

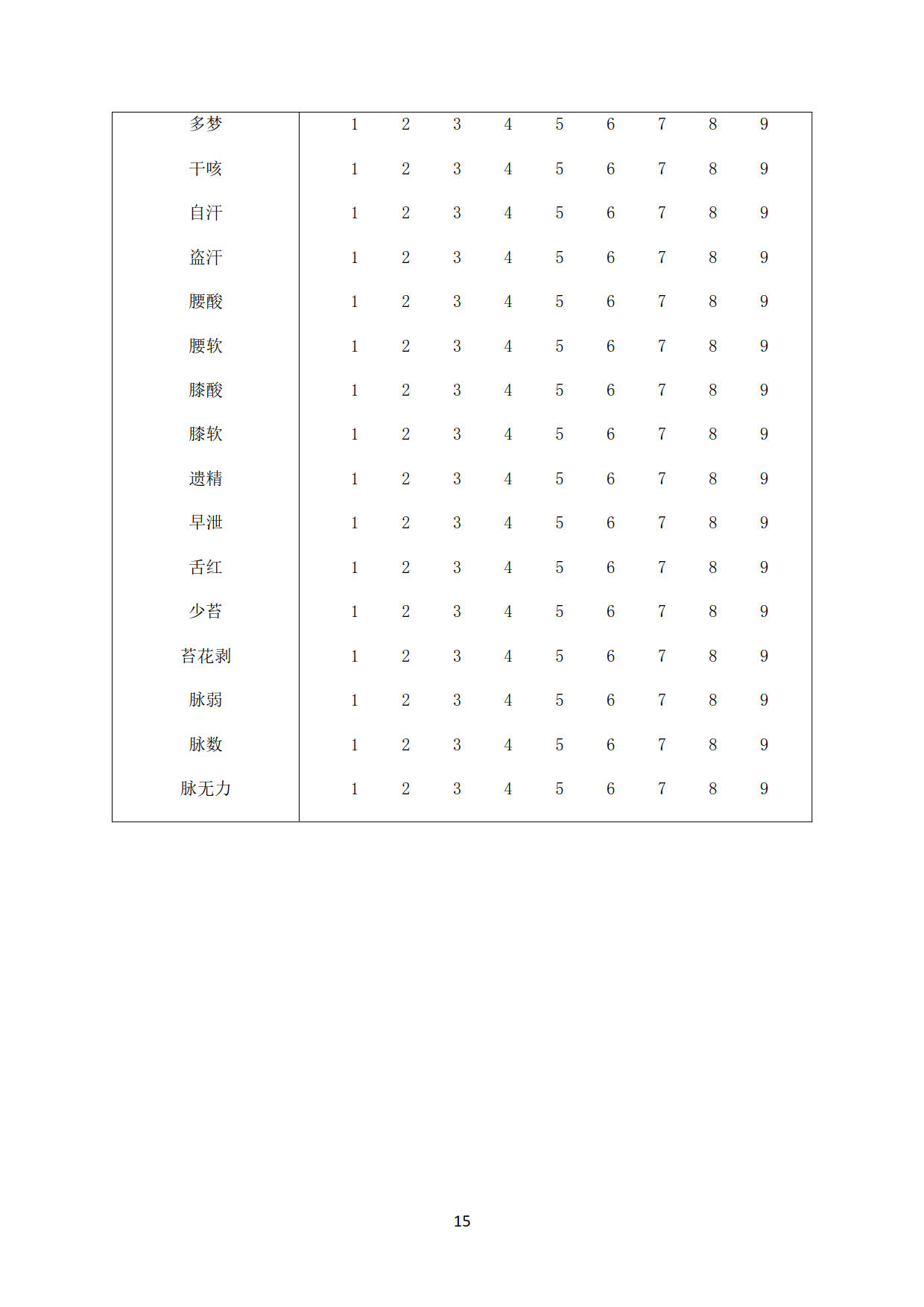

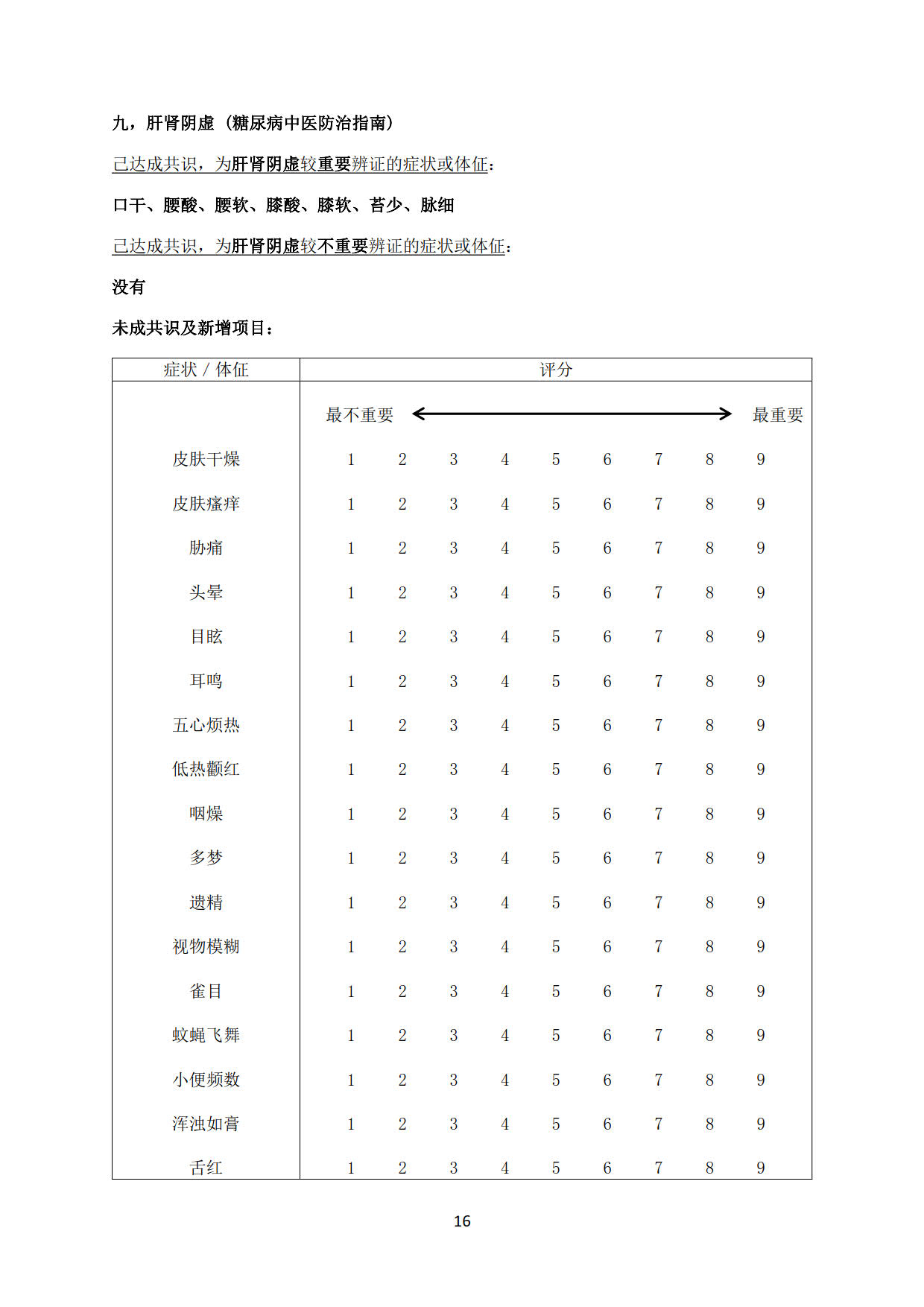

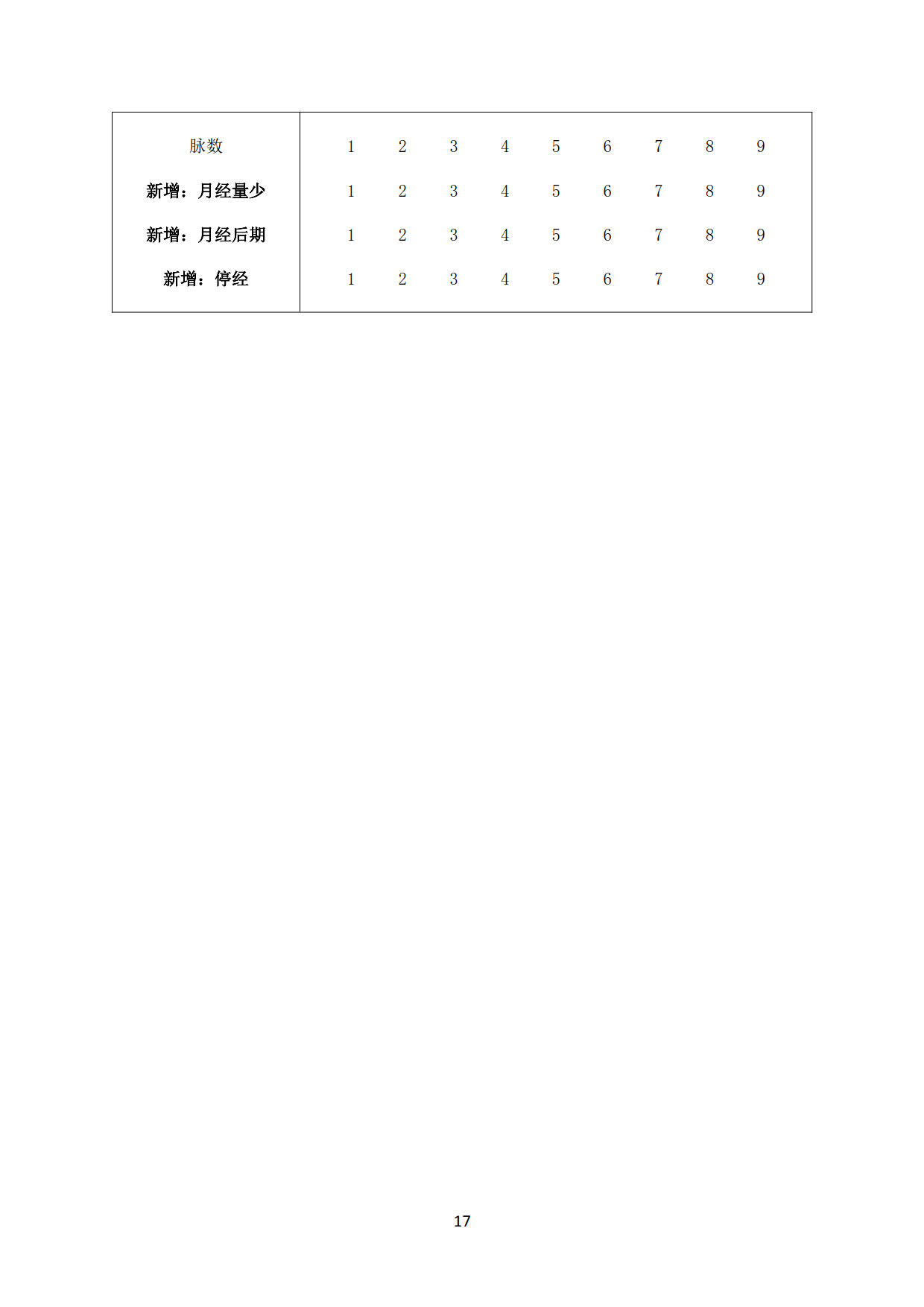

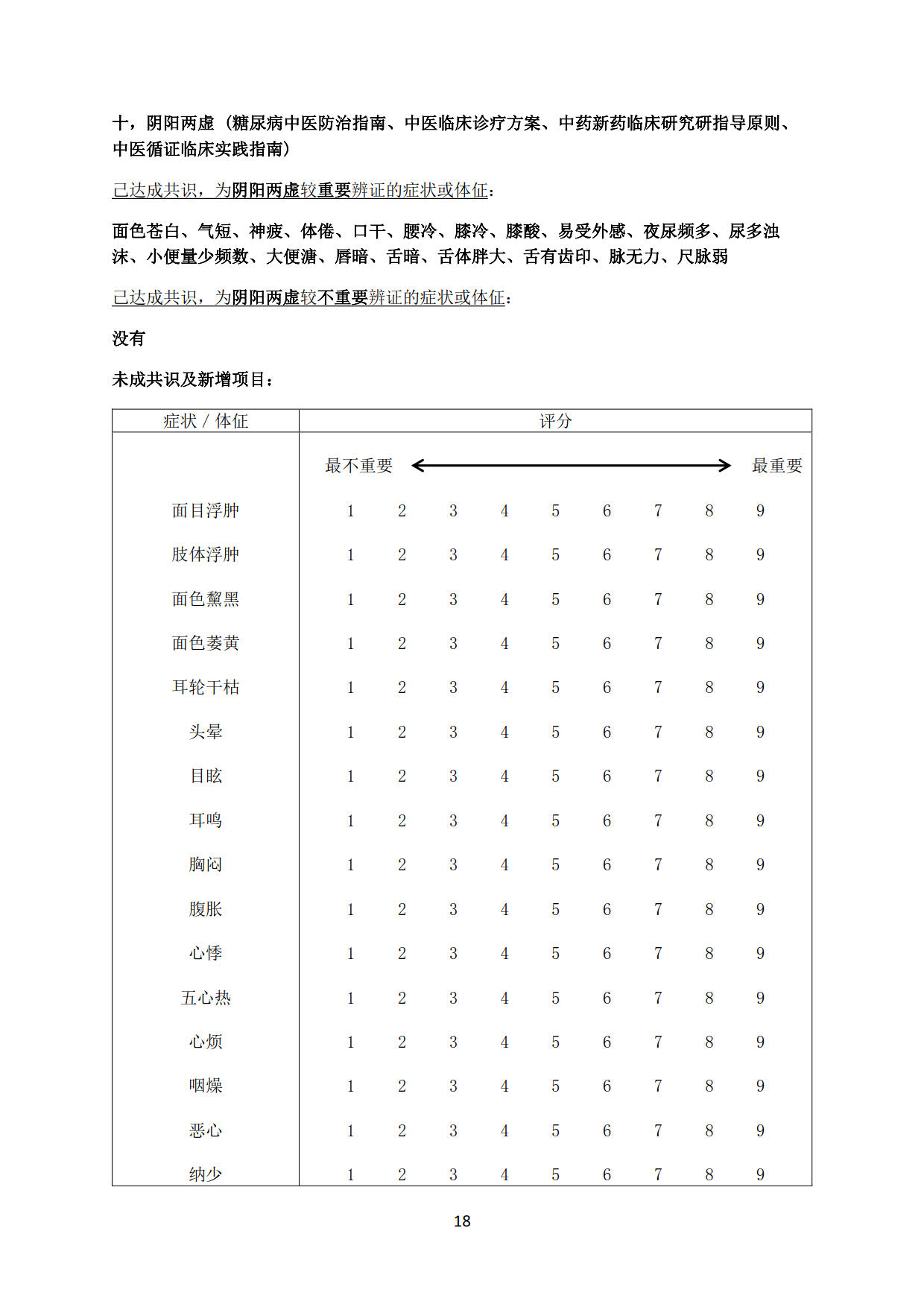

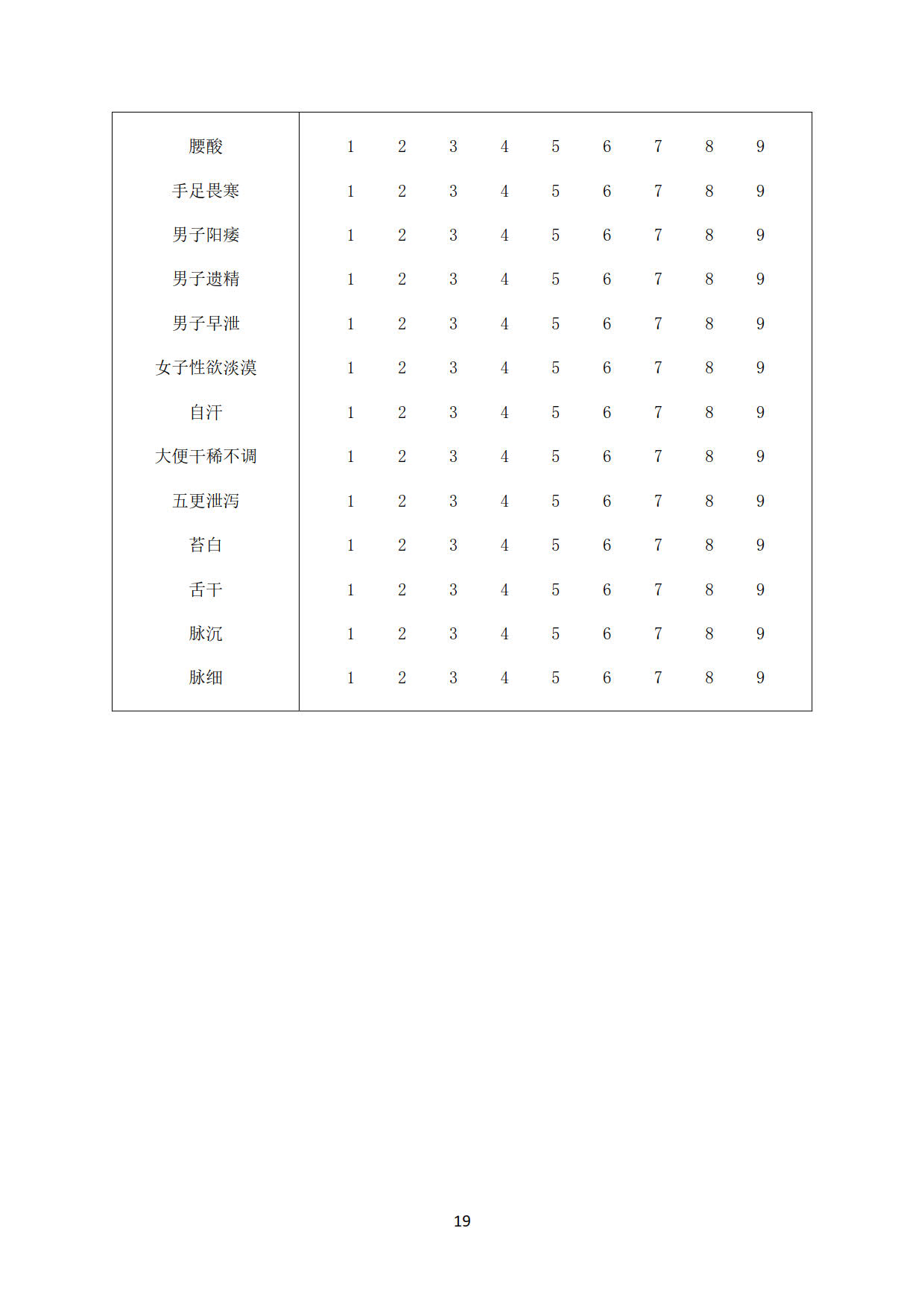

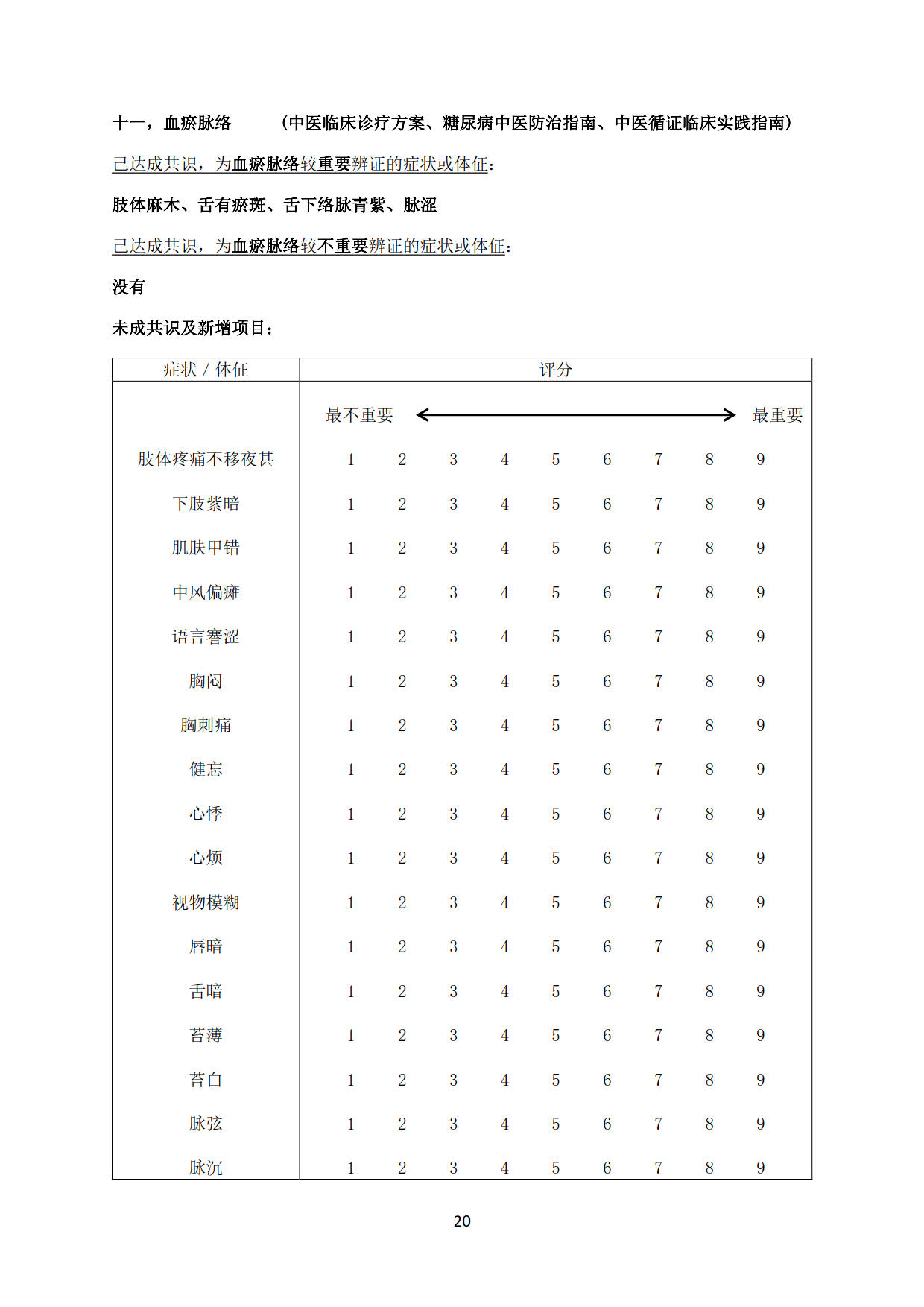

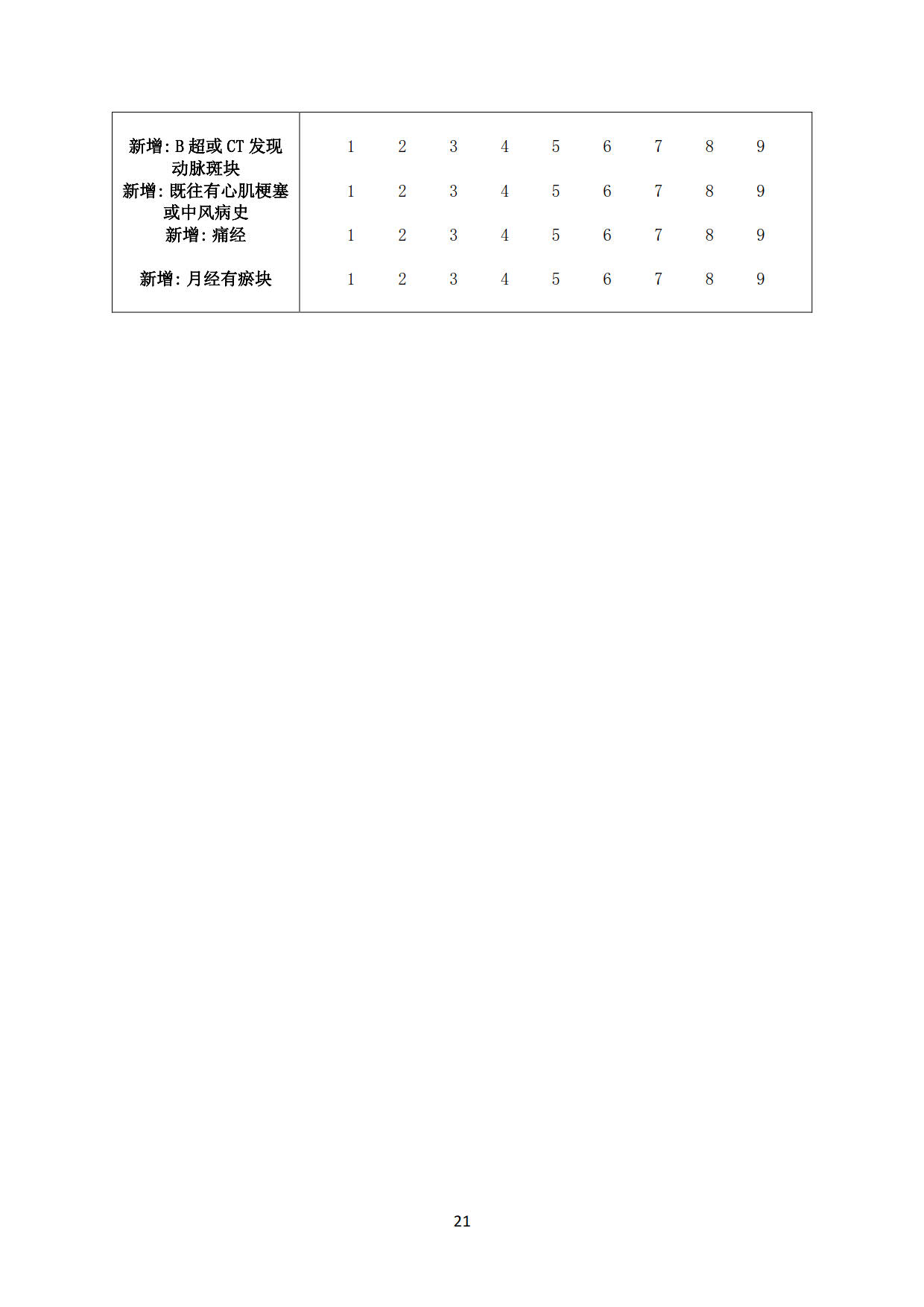

Supplement: Supplementary file 1 [file Data_Sheet_1.docx]
